# Advancing Yeast Identification Using High‐Throughput DNA Barcode Data From a Curated Culture Collection

**DOI:** 10.1111/1755-0998.70082

**Published:** 2025-11-26

**Authors:** Duong Vu, Michel de Vries, Bert Gerrits van den Ende, Jos Houbraken, R. Henrik Nilsson, Balázs Brankovics, Margarita Hernández‐Restrepo, Johannes Z. Groenewald, Pedro W. Crous, Ferry Hagen, Wieland Meyer, Gerard J. M. Verkley, Marizeth Groenewald

**Affiliations:** ^1^ Westerdijk Fungal Biodiversity Institute Utrecht the Netherlands; ^2^ Department of Biological & Environmental Sciences, Gothenburg Global Biodiversity Centre University of Gothenburg Göteborg Sweden

**Keywords:** DNA barcoding, eDNA metabarcoding, similarity cutoff, yeast identification

## Abstract

Yeast identification is essential in fields ranging from microbiology and biotechnology to food science and medicine. While DNA barcoding has become the standard for identifying cultured strains, environmental DNA (eDNA) metabarcoding has revolutionised microbial community profiling, providing deeper insights into yeast communities across diverse ecosystems. A major challenge in DNA (meta)barcoding remains the limited availability of high‐quality reference sequences, which are critical for accurate species identification and comprehensive taxonomic profiling of both environmental and clinical samples. To address this gap, the Westerdijk Fungal Biodiversity Institute (WI) launched a DNA barcoding initiative in 2006 to generate high‐quality, often type‐derived ITS and LSU barcodes for all ~100,000 fungal strains preserved in the CBS culture collection, including approximately 15,000 yeasts. Building on the yeast barcode dataset released in 2016, we now present an expanded set of 2856 ITS and 3815 LSU sequences, representing 911 and 1137 yeast species, respectively. Notably, 27%–29% of these sequences are derived from ex‐type cultures. Using both newly generated and previously published barcodes, we assess the taxonomic resolution of commonly used yeast metabarcoding markers (ITS, ITS1, ITS2 and LSU) and propose marker‐specific similarity cutoffs for different yeast taxonomic groups. These results provide actionable guidance for marker selection and improve the interpretation of metabarcoding data. We further demonstrate the impact of well‐curated reference databases with up‐to‐date taxonomy by reanalyzing Human Microbiome Project data, revealing how diet and environment shape the gut mycobiota.

## 1 Introduction

Yeast identification is crucial in microbiology, food science, brewing, medicine and biotechnology (Kurtzman et al. 2011). DNA barcoding has become the preferred method for identifying individual species from cultured isolates, surpassing traditional morphology‐based approaches due to its higher accuracy, reliability and reproducibility. DNA barcoding relies on sequencing short, standardised genomic regions that exhibit sufficient variation to distinguish species. The primary DNA barcode markers used for yeast identification are the D1/D2 domain of the large subunit (LSU) nuclear rRNA gene and the nuclear ribosomal internal transcribed spacer (ITS) region. Although the ITS region has been designated as the official fungal barcode (Schoch et al. 2012), LSU‐based sequencing has historically had the greatest impact on yeast systematics—particularly among ascomycetous yeasts—largely owing to the pioneering work of Kurtzman and Robnett (1997, 1998), whose high‐quality sequences and systematic framework continue to underpin modern yeast taxonomy.

DNA metabarcoding has further transformed the study of yeast diversity and ecology by enabling culture‐independent, large‐scale community profiling, further enhancing our understanding of yeast communities across diverse ecosystems. Applications of yeast metabarcoding can be found, for example, in: (1) studying yeast diversity in soil, water and extreme environments (Detheridge et al. 2018; Rosa, Pinto, et al. 2020; Rosa, da Silva, et al. 2020; Boekhout et al. 2022; Martin‐Sanchez et al. 2021); (2) monitoring yeast communities in fermentation processes (e.g., wine, beer and cheese) within the food and beverage industry (Sternes et al. 2017; Fernández‐Niño et al. 2021; Han et al. 2025); and (3) investigating yeast microbiomes in human and animal hosts, including gut and skin microbiota, in medical and clinical research (Dominguez‐Bello et al. 2010; LaTuga et al. 2011; Heisel et al. 2015; Strati et al. 2016; Nash et al. 2017; Lavrinienko et al. 2018; van Thiel et al. 2022).

The most widely used DNA barcode markers in fungal and yeast metabarcoding studies are the internal transcribed spacer (ITS) regions—ITS1, ITS2 and the full ITS—as well as the large subunit (LSU), particularly the D1/D2 domains. ITS is highly effective for metabarcoding due to its high variability and broad taxonomic coverage (Schoch et al. 2012), but it is less commonly used as the primary criterion for formal yeast species recognition. In yeasts, especially ascomycetous species, the LSU D1/D2 region remains the gold standard for species delineation, offering robust resolution and high‐quality reference sequences essential for accurate identification. Its more conserved sequences provide reliable genus‐level resolution and often complement ITS in distinguishing species that ITS alone cannot resolve. However, the lower variability of LSU can limit fine‐scale species differentiation, making it less sensitive than ITS for profiling highly diverse yeast communities, such as those found in fermented foods (Rué et al. 2023).

Although DNA (meta)barcoding is widely used, it faces several challenges. A major limitation is the lack of reference barcodes for many fungal species. Accurate identification depends on complete, reliable databases. While estimates suggest ~2.5 million fungal species (Niskanen et al. 2023) and ~20,000 yeast species (Boekhout et al. 2022), only ~160,000 fungal names are accepted, and just ~50,000 have publicly available barcodes—few of which are type‐derived (Abarenkov et al. 2024). Many existing barcodes are of low quality or lack full taxonomic annotation (Abarenkov et al. 2022). As Nash et al. (2017) observed in a metabarcoding study of the healthy human gut microbiome (The Human Microbiome Project Consortium 2012), fungal databases remain sparse and poorly curated compared to bacterial ones. This highlights the urgent need for curated, authenticated reference sequences like those from the NCBI RefSeq Targeted Loci Project (Schoch et al. 2014; Irinyi et al. 2015). Another challenge is the highly imbalanced taxonomic classification of filamentous fungi and yeasts, which complicates clear delineation between groups (Lücking et al. 2020; Vu et al. 2020).

Moreover, variability of ITS and LSU regions may exist within a single strain, as demonstrated in species such as *Clavispora lusitaniae* (Lachance et al. 2003), *Trichaptum abietinum* (Kauserud and Schumacher 2003), *Geotrichum candidum* (Alper et al. 2011; Groenewald et al. 2012), and the medically important species *C. glabrata* , *C. albicans* and *C. parapsilosis* (Colabella et al. 2021). This significant intra‐strain variation in these commonly used barcoding regions can result in artificial inflation of species richness, misidentification or the failure to match sequences to reference databases. Distinguishing true biological diversity from sequencing artefacts becomes increasingly difficult when this heterogeneity exceeds inter‐species divergence.

In addition to intra‐strain heterogeneity, inter‐strain variability also complicates accurate species delineation. In highly polymorphic taxa, such as *Metschnikowia pulcherrima*, pronounced sequence divergence among strains has led to over‐splitting and multiple synonyms, reflecting taxonomic inflation rather than true diversity (Sipiczki 2020). The recently described *Clavispora paralusitaniae* similarly demonstrates how inter‐strain divergence can obscure species boundaries (Chai et al. 2022). These cases underscore the need for curated, type‐derived reference sequences and marker‐specific similarity thresholds to distinguish genuine species diversity from artefactual variation.

Finally, the sequence variation and resolving power of different barcodes for yeast identification are not well studied. Vu et al. (2016, 2019) demonstrated that 17% and 18% of filamentous fungal species, and 6% and 9.5% of yeast species, are indistinguishable using ITS and/or LSU barcodes. Additionally, ITS1 appears to have higher sequence variation compared to ITS2, making it more useful for distinguishing closely related fungal species, but it fails to resolve some yeast species, such as in *Malassezia* (Hoffmann et al. 2013; Nash et al. 2017). Conversely, ITS2 is often preferred for studying mixed fungal communities, as it sometimes provides better taxonomic resolution in filamentous fungi (Vu et al. 2022), is less prone to PCR bias due to its more consistent length, primer availability, and is better covered in reference databases, such as UNITE (Kõljalg et al. 2005, 2013; Nilsson et al. 2019; Abarenkov et al. 2024).

A clear examination of sequence variation and the resolving power of different biomarkers for yeast identification would help researchers select the appropriate biomarkers and better interpret the results of their metabarcoding studies.

At the Westerdijk Fungal Biodiversity Institute (WI), we maintain the CBS culture collection, which includes over 100,000 fungal strains from more than 20,000 species—of which approximately 15,000 are yeasts, representing over 3000 species. As of July 2025, the collection contains around 15,000 ex‐type strains, including 3000 yeast ex‐types. To enhance reference barcodes for fungal identification, the WI‐DNA barcoding project aims to generate barcodes from two genetic markers, ITS and LSU, for all strains preserved in our collection (Summerbell et al. 2005; Tillier et al. 2006). In 2016, we publicly released a large yeast barcode dataset covering 80% of the ~2000 yeast species in the collection, enabling us to establish similarity cutoffs for species delimitation (Vu et al. 2016).

As the number of public DNA barcodes increased, it became clear that the field of metabarcoding loses significant resolution and scientific explanatory power by relying on a single sequence similarity cutoff for taxonomic assignment (Abarenkov et al. 2016; Vu et al. 2016, 2019, 2022). In our previous work, we showed that the resolving powers and similarity cutoffs differ between *Dothideomycetes*, *Eurotiomycetes* and *Sordariomycetes* for filamentous fungal identification (Vu et al. 2019) and between *Ascomycota* and *Basidiomycota* for yeast species and genus identification (Boekhout et al. 2021). In Vu et al. (2022), we introduced *dnabarcoder*, an open‐source tool that allows us to study the resolving power of different biomarkers and predict similarity cutoffs for various fungal taxonomic groups, maximising taxonomic resolution for fungal identification.

As the demand for reference data grows, driven by the popularity of metabarcoding and the increasing recognition of dark taxa, we now release a new yeast barcode dataset comprising 2856 ITS and 3815 LSU barcodes, representing 917 and 1137 yeast species, respectively. Notably, 27% of the ITS and 29% of the LSU barcodes are derived from the nomenclatural type.

We combine this dataset with that of Vu et al. (2016) to conduct an in‐depth analysis of the resolving power of the biomarkers—ITS (covering partial small subunit [SSU], complete ITS1‐5.8S‐ITS2 and partial LSU), ITS1, ITS2 and LSU—as well as their optimal similarity cutoffs for yeast identification across different taxonomic groups. Our combined dataset furthermore enables us to evaluate how recent taxonomic revisions have affected genus‐level resolution in yeast identification.

Finally, we emphasise the crucial role of curated reference databases with updated taxonomy and marker‐specific similarity cutoffs for accurate sequence identification in metabarcoding. By reanalyzing the human gut mycobiome dataset from Nash et al. (2017) using our curated barcode resources—the yeast barcode dataset presented in this study and the filamentous fungal dataset from Vu et al. (2019)—we demonstrate that database quality is more influential than database size. Despite containing fewer than 20,000 sequences combined, these curated datasets yielded robust and biologically meaningful results, revealing a strong influence of diet and living environment on gut mycobiota composition.

## 2 Materials and Methods

### 2.1 Datasets

#### 2.1.1 The CBS Yeast Barcode Dataset

The ITS and LSU barcodes of the CBS collection were generated under the WI‐DNA barcoding project (Vu et al. 2012) following protocols from Stielow et al. (2015). ITS sequences were amplified using primers ITS5 and ITS4 (White et al. 1990), covering partial SSU, complete ITS1‐5.8S‐ITS2 and partial LSU. The LSU sequences targeted the D1/D2 domain using primers LR0R (Vilgalys and Sun 1994) and LR5 (Vilgalys and Hester 1990).

For validation, sequences were first BLASTed (Altschul et al. 1997) against NCBI's GenBank nucleotide database (Sayers et al. 2025) for taxonomic assignment. Species names corresponding to the DNA barcode sequences generated in this study and their higher order classification were updated to reflect the name changes published by Groenewald et al. (2023), in which 11 new order names, viz. *Alaninales*, *Alloascoideales*, *Ascoideales*, *Dipodascales*, *Lipomycetales*, *Phaffomycetales*, *Pichiales*, *Saccharomycodales*, *Serinales*, *Sporopachydermiales* and *Trigonopsidales*, were introduced together with the existing *Saccharomycetales*. The taxonomic information of the sequences was downloaded from the CBS culture collection and MycoBank (Robert et al. 2013) in November 2024 and is provided in Appendix S1. In this file, sequence IDs beginning with ‘WI’ indicate newly generated barcodes, while all others correspond to GenBank accession numbers.

To support further validation, we inferred order‐level phylogenetic trees using both newly generated barcodes and those previously published in Vu et al. (2016) under BioProject PRJNA351778 (https://www.ncbi.nlm.nih.gov/bioproject/PRJNA351778) to remove mislabeled sequences that appeared phylogenetically distant from other members of their respective clades. The final ITS and LSU phylogenetic trees, covering 34 yeast orders, each with at least three sequences, are provided in Appendices S7 and S8.

In total, we obtained 2856 new ITS barcodes representing 2856 strains from 917 yeast species, and 3815 LSU barcodes representing 3815 strains from 1136 yeast species. These are referred to as the newITS and newLSU datasets in Table 1 and are made public through this study. Among these, 775 ITS (27%) and 1116 LSU (29%) barcodes were derived from type material.

**TABLE 1:** Sequence and taxonomic group counts across yeast and filamentous fungal datasets.

Dataset	Seq. no		Strain level	Species level	Genus level	Family level	Order level	Class level	Phylum level
newITS	2856	Seq. no.	2856	2856	2856	2687	2856	2856	2856
		Group no.	2856	917	168	55	38	17	2
newLSU	3815	Seq. no.	3815	3815	3815	3661	3809	3815	3815
		Group no.	3815	1137	197	58	37	17	2
yeastITS	7190	Seq. no.	7190	7190	7190	6914	7176	7190	7190
		Group no.	6862	1607	217	61	38	18	2
yeastITS1	7184	Seq. no.	7185	7185	7185	6909	7171	7185	7185
		Group no.	6857	1605	217	61	38	18	2
yeastITS2	7104	Seq. no.	7105	7105	7105	6829	7091	7105	7105
		Group no.	6777	1567	209	61	38	18	2
yeastLSU	7912	Seq. no.	7912	7912	7912	7615	7896	7912	7912
		Group no.	7374	1663	223	62	39	19	2
filfungalITS2	11,674	Seq. no.	11,674	11,651	11,657	11,237	11,312	11,491	11,646
		Group no.	11,494	6048	1679	454	145	38	9
CBSITS2	18,779	Seq. no.	18,779	18,756	18,762	18,066	18,403	18,596	18,571
		Group no.	18,272	7607	1874	502	167	46	9
UNITE	2,152,309	Seq. no.	n/a	680,423	1,772,204	1,840,653	2,002,482	2,032,433	2,084,826
		Group no.	n/a	47,753	6432	981	303	87	19
yeastUNITE	124,785	Seq. no.	n/a	56,395	114,057	101,098	122,225	124,785	124,785
		Group no.	n/a	2736	363	97	44	13	2

*Note:* This table summarises the number of sequences (Seq. no.) and corresponding taxonomic groups (Group no.) at multiple taxonomic levels—from strain to phylum—for each dataset, including CBS newITS, newLSU, yeastITS, yeastITS1, yeastITS2, yeastLSU, CBSITS2, UNITE and yeast UNITE.

To facilitate a comprehensive analysis of the resolving power and optimal similarity cutoffs of ITS, ITS1, ITS2 and LSU for yeast identification, we constructed the *yeastITS, yeastITS1, yeastITS2* and *yeastLSU* datasets by combining sequences from both newly generated barcodes with those from Vu et al. (2016). Table 1 summarises the number of sequences, strains, genera, families, orders, classes and phyla represented in each dataset. The resolving power and optimal similarity thresholds of each marker are assessed in detail in Section 3.

#### 2.1.2 The CBS Filamentous Fungal Dataset

To assess the gut microbiome of healthy humans (Nash et al. 2017), the CBS filamentous fungal ITS2 dataset (Vu et al. 2022), referred to as filfungalITS2 in Table 1, was used in addition to the expanded yeast barcode dataset presented in this study.

FilfungalITS2 was derived from the filamentous fungal ITS barcodes released by Vu et al. (2019) and updated with taxonomic information from the CBS culture collection database and MycoBank as of May 2025. The full taxonomic metadata is provided in Appendix S1.

Similarity cutoffs for different filamentous fungal taxonomic groups were recomputed using *dnabarcoder* (Vu et al. 2022) with updated taxon names and are available in Appendix S3.

Together with the herein expanded yeastITS2, we created a well‐curated CBSITS2 reference dataset, enriched with current taxonomic information down to the species level, for fungal identification. Table 1 summarizes its contents, including sequences, strains and taxonomic affiliation.

#### 2.1.3 The Human Microbiome Dataset

To highlight the importance of well‐curated reference databases and marker‐specific similarity cutoffs for accurate sequence identification in metabarcoding studies, we reanalyzed the human microbiome dataset published by Nash et al. (2017) using our reference barcode datasets filfungalITS2 and yeastITS2 annotated with the most up‐to‐date taxonomic names. The human microbiome dataset includes 317 stool samples from 147 healthy individuals, collected as part of the NIH Human Microbiome Project (The Human Microbiome Project Consortium 2012), which aimed to characterise the composition of the ‘healthy’ human microbiome.

In Section 3.7, we examine whether classifying the gut samples against *yeastITS2* and *filfungalITS2* using *dnabarcoder* enhances taxonomic identification. The classification method is described in detail in Section 2.2.

#### 2.1.4 The UNITE and yeastUNITE Datasets

To evaluate the completeness of our *yeastITS* dataset, we compared its taxonomic coverage with that of the UNITE reference dataset (Kõljalg et al. 2005), one of the largest ITS resources for fungal identification. We downloaded the latest ‘UNITE+INSD’ release (Abarenkov et al. 2024), referred to as UNITE in Table 1, and extracted ITS sequences from 18 yeast classes (*Agaricostilbomycetes*, *Cystobasidiomycetes*, *Dipodascomycetes*, *Exobasidiomycetes*, *Lipomycetes*, *Malasseziomycetes*, *Microbotryomycetes*, *Moniliellomycetes*, *Pichiomycetes*, *Saccharomycetes*, *Schizosaccharomycetes*, *Spiculogloeomycetes*, *Sporopachydermiomycetes*, *Taphrinomycetes*, *Tremellomycetes*, *Trigonopsidomycetes*, *Ustilaginomycetes* and *Xylonomycetes*) present in *yeastITS*. This yielded 124,756 sequences representing 2726 species, 316 genera, 96 families and 43 orders across 2 phyla (referred to as *yeastUNITE*, Table 1). A detailed comparison between the CBS and UNITE barcode datasets is provided in Section 3.1.

### 2.2 Methods

We used dnabarcoder (Vu et al. 2022, available at https://github.com/vuthuyduong/dnabarcoder) to: (Abarenkov et al. 2016) analyse the CBS yeast barcode datasets including their taxonomic classification as well as the intra‐specific variation within taxonomic groups (Abarenkov et al. 2022); predict global and local similarity cutoffs and resolving powers of the biomarkers ITS, ITS1, ITS2 and LSU for yeast identification; and (Abarenkov et al. 2024) reclassify the human microbiome dataset against the yeastITS2 and filfungalITS2 datasets using the similarity cutoffs predicted for yeast and filamentous fungal identification.

Dnabarcoder calculates the similarity score between two DNA sequences based on the BLAST percent identity (s), provided that the BLAST alignment length (l) exceeds a specified minimum length (m). If the alignment length is shorter than the minimum, the similarity score is adjusted using the formula: *s* × *l*/*m*. To ensure meaningful DNA comparisons when using *dnabarcoder*, for ITS and LSU regions, we set a minimum alignment length of 400 bases (using ‐ml 400) option; for ITS1 and ITS2, this minimum was 50 bases (‐ml 50). This threshold helps prevent short sequences from appearing artificially similar to all others, a known issue described by Vu et al. (2022).

Dnabarcoder predicts the resolving power of a barcode marker in a taxonomic group to delineate sequences at a given taxonomic level as the highest *F*‐measure obtained when clustering the sequences of that group at various similarity scores using dnabarcoder's predict command (Vu et al. 2022). A taxonomic group refers to a set of sequences that share the same taxonomic classification at a specific rank. The similarity cutoff is the similarity score associated with the highest F‐measure. Note that the F‐measure is a widely adopted metric in clustering, quantifying how well the clustering results match a known classification (ground truth) (Paccanaro et al. 2006).

The global similarity cutoff refers to the similarity cutoff predicted to delineate sequences across the entire dataset at a given taxonomic level, whereas the local similarity cutoff refers to the similarity cutoff predicted to delineate sequences within a taxonomic group of the dataset.

When the obtained resolving power is low—either due to sequencing artefacts or the need for reclassification of the taxonomic group—the predicted cutoff may be inaccurate. In such cases, dnabarcoder will predict the best cutoff using dnabarcoder's best command. This function determines the similarity cutoff as the one predicted for the higher‐level taxonomic group that achieves the highest *F*‐measure (Vu et al. 2022).

To infer the ITS and LSU phylogenetic trees, we used MAFFT v7.409 (Katoh and Standley 2013) for multiple sequence alignment and IQ‐TREE v.1.6.1 (Nguyen et al. 2015) for tree inferences. Additionally, we used ITSx v.1.1.1 (http://microbiology.se/software/itsx) to extract ITS1 and ITS2 sequences from the ITS sequences.

## 3 Results

We evaluated CBS yeast barcodes (yeastITS and yeastLSU) and their resolving power for yeast identification by: (Abarenkov et al. 2016) analysing their taxonomic classification, sequence distribution, and intra‐specific variation within different yeast taxonomic groups (Abarenkov et al. 2022); calculating the number of indistinguishable yeast species using ITS and LSU (Abarenkov et al. 2024); predicting global and local similarity cutoffs and the resolving power of different biomarkers (viz. ITS, ITS1, ITS2 and LSU) based on the CBS yeast barcode datasets; and (Alper et al. 2011) examining whether recent name changes in 2024 (Liu, Hu, Yurkov, et al. 2024; Liu, Hu, Zhao, et al. 2024; Zhu et al. 2024) improved the resolving power of ITS and LSU for yeast identification. We highlighted the importance of well‐curated databases with up‐to‐date taxonomy by reclassifying the human microbiome dataset using the yeastITS2 and filfungalITS2 datasets with predicted similarity cutoffs, and by comparing the results with those from Nash et al. (2017).

### 3.1 Analysing the CBS Yeast Barcodes for Yeast Identification

#### 3.1.1 Taxonomic Classification of the yeastITS and yeastLSU Datasets

**FIGURE 1:**
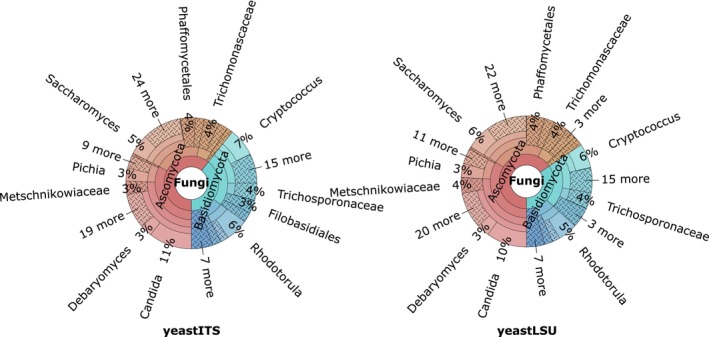
Taxonomic composition of the yeastITS and yeastLSU datasets. Krona charts illustrate the distribution of sequences across taxonomic levels for the yeastITS dataset (left) and the yeastLSU dataset (right). The charts were generated using dnabarcoder's distribution command with the ‐method krona option, highlighting differences in taxonomic coverage and diversity between the two barcode datasets. These Krona charts are also provided in Appendices S9 and S13, respectively.

The Krona charts in Figure 1 reveal a strong taxonomic imbalance in the yeastITS and yeastLSU datasets, where a few groups dominate with many subgroups and strains, resulting in disproportionately high sequence counts. The five largest groups at each taxonomic level in the yeastITS/LSU dataset are: at the species level, *Cryptococcus neoformans* , *Rhodotorula mucilaginosa* , *Saccharomyces cerevisiae* , *Debaryomyces hansenii* and *Saitozyma podzolica*, accounting for 11.2%/11.7% of the sequences; at the genus level, *Candida*, *Cryptococcus*, *Rhodotorula*, *Saccharomyces* and *Pichia* (31.4%/31.7%); at the family level, *Debaryomycetaceae*, *Saccharomycetaceae*, *Sporidiobolaceae*, *Cryptococcaceae* and *Pichiaceae* (60.2%/63.5%); at the order level, *Serinales*, *Saccharomycetales*, *Tremellales*, *Sporidiobolales* and *Pichiales* (68%/67.5%); and at the class level, *Pichiomycetes*, *Tremellomycetes*, *Saccharomycetes*, *Microbotryomycetes* and *Dipodascomycetes* (93%/93.1%).

A similar pattern is observed in the yeastUNITE database, where a small number of dominant taxa account for the majority of sequences. At the species level, the top five species— *Candida albicans* , *Candida parapsilosis* , *Saccharomyces cerevisiae* , *Candida tropicalis* and *Meyerozyma guilliermondii*—represent over 22.7% of all sequences. At the genus level, *Candida*, *Saccharomyces*, *Pichia*, *Rhodotorula* and *Cryptococcus* comprise more than 18.35%. This concentration increases at higher taxonomic levels, with the top five families (*Trimorphomycetaceae*, *Piskurozymaceae*, *Saccharomycetaceae*, *Debaryomycetaceae* and *Trichosporonaceae*) covering over 45.73%, the top five orders exceeding 68.9% (*Saccharomycetales*, *Tremellales*, *Filobasidiales*, *Trichosporonales* and *Sporidiobolales*), and the top five classes—*Tremellomycetes*, *Saccharomycetes*, *Microbotryomycetes*, *Malasseziomycetes* and *Ustilaginomycetes*—accounting for more than 93.5% of the sequences.

This imbalance can lead to biased classification models that favour dominant taxa, as shown in Vu et al. (2020). When some taxa are much better represented in the training dataset, certain classification models tend to assign sequences from underrepresented taxa—or those lacking close reference sequences—to these dominant groups. Consequently, this reduces prediction accuracy for minority taxa and leads to biased or incorrect taxonomic assignments in metabarcoding studies.

Compared to the yeastUNITE dataset, the yeastITS dataset contains only 5.8% of the total sequences in yeastUNITE but represents over 44.6% of species, 53% of genera, 58% of families and 59% of orders present in yeastUNITE. Our dataset added 387 new or updated species names to UNITE (see Appendix S1), encompassing 24 genera, namely *Arthroascus*, *Arxiozyma*, *Australozyma*, *Candidozyma*, *Cylindricascospora*, *Danielia*, *Farysizyma*, *Gabaldonia*, *Gaillardinia*, *Guehomyces*, *Henningerozyma*, *Isabelozyma*, *Jamesozyma*, *Helenozyma*, *Huiozyma*, *Maudiozyma, Monosporozyma*, *Osmozyma*, *Sungouiella*, *Sungouiozyma*, *Rhodosporidium*, *Tanozyma*, *Tardiomyces* and *Wilhelminamyces*; three families, viz. *Pachysolenaceae*, *Sporopachydermiaceae* and *Wickerhamomycetaceae*; 11 orders, viz. *Alaninales*, *Ascoideales*, *Curvibasidiales*, *Dipodascales*, *Lipomycetales*, *Phaffomycetales*, *Pichiales*, *Saccharomycodales*, *Serinales*, *Sporopachydermiales* and *Trigonopsidales*; and five classes, viz. *Dipodascomycetes*, *Lipomycetes*, *Pichiomycetes*, *Sporopachydermiomycetes* and *Trigonopsidomycetes*. These new families, orders and classes were absent from UNITE because they were only recently introduced in the taxonomic revisions by Groenewald et al. (2023).

#### 3.1.2 Distribution of CBS Yeast Barcodes Based on Sequence Similarity

**FIGURE 2:**
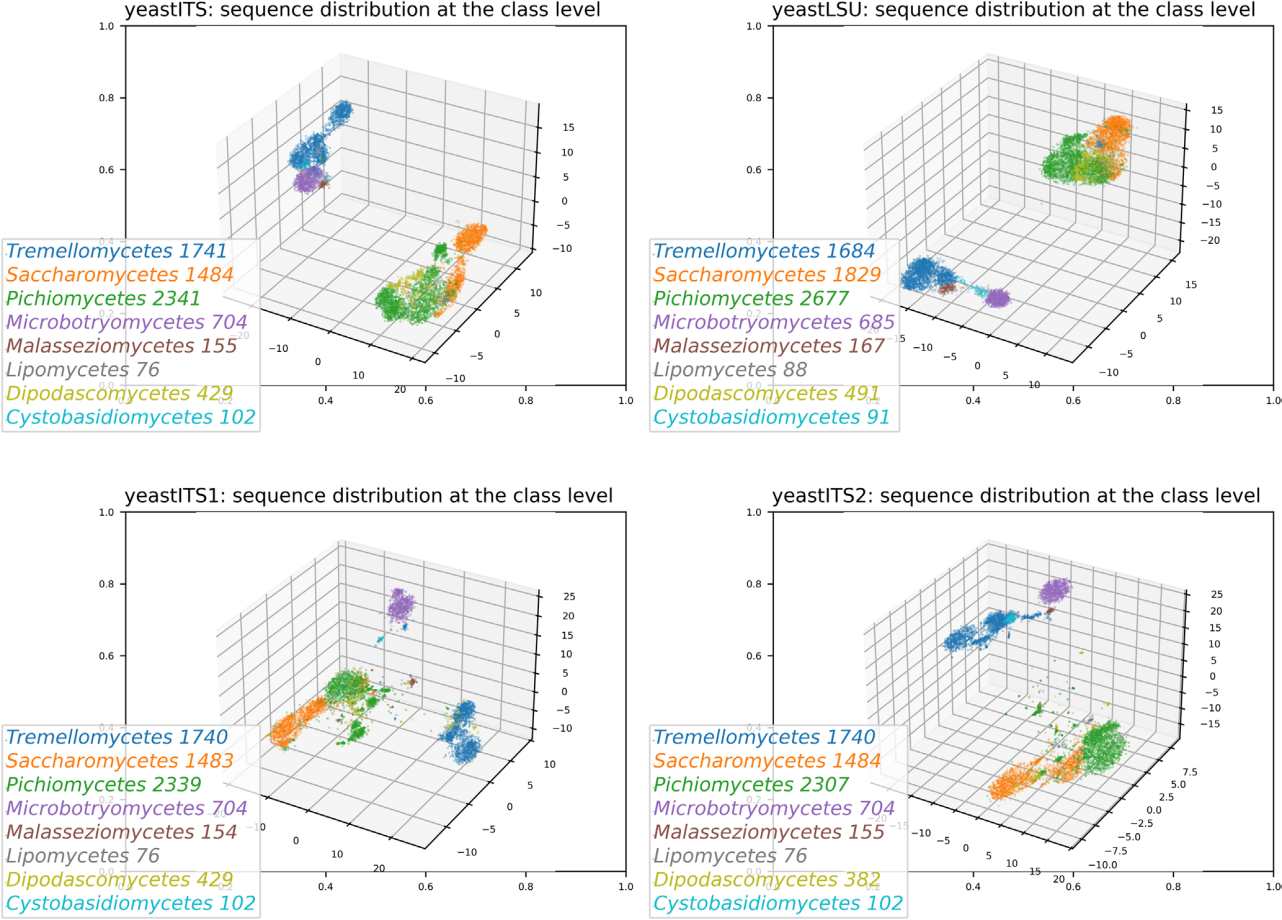
Sequence similarity and taxonomic distribution across yeast ITS and LSU datasets. This figure visualises the sequence distribution of the yeastITS, yeastLSU, yeastITS1 and yeastITS2 datasets. Sequence similarity matrices were computed using BLAST via dnabarcoder's sim command (‐ml 400 for long barcodes such as ITS and LSU; ‐ml 50 for short barcodes such as ITS1 and ITS2). Sequences were projected into three‐dimensional space using Matplotlib's mplot3d toolkit and coloured according to taxonomic class using dnabarcoder's visualize command, revealing clustering patterns and variation across both taxonomic groups and datasets.

Figure 2 shows sequence similarity distributions in the yeastITS, yeastITS1, yeastITS2 and yeastLSU datasets, revealing that some yeast classes cluster closely while others are more distant, suggesting that using group‐specific similarity cutoffs may improve yeast identification.

#### 3.1.3 The Intra‐Specific Variation of Yeast Barcodes Within Different Taxonomic Groups

To further investigate the similarity cutoffs for yeast identification, we computed the similarity values within yeast taxonomic groups using ITS, ITS1, ITS2 and LSU barcodes.

**FIGURE 3:**
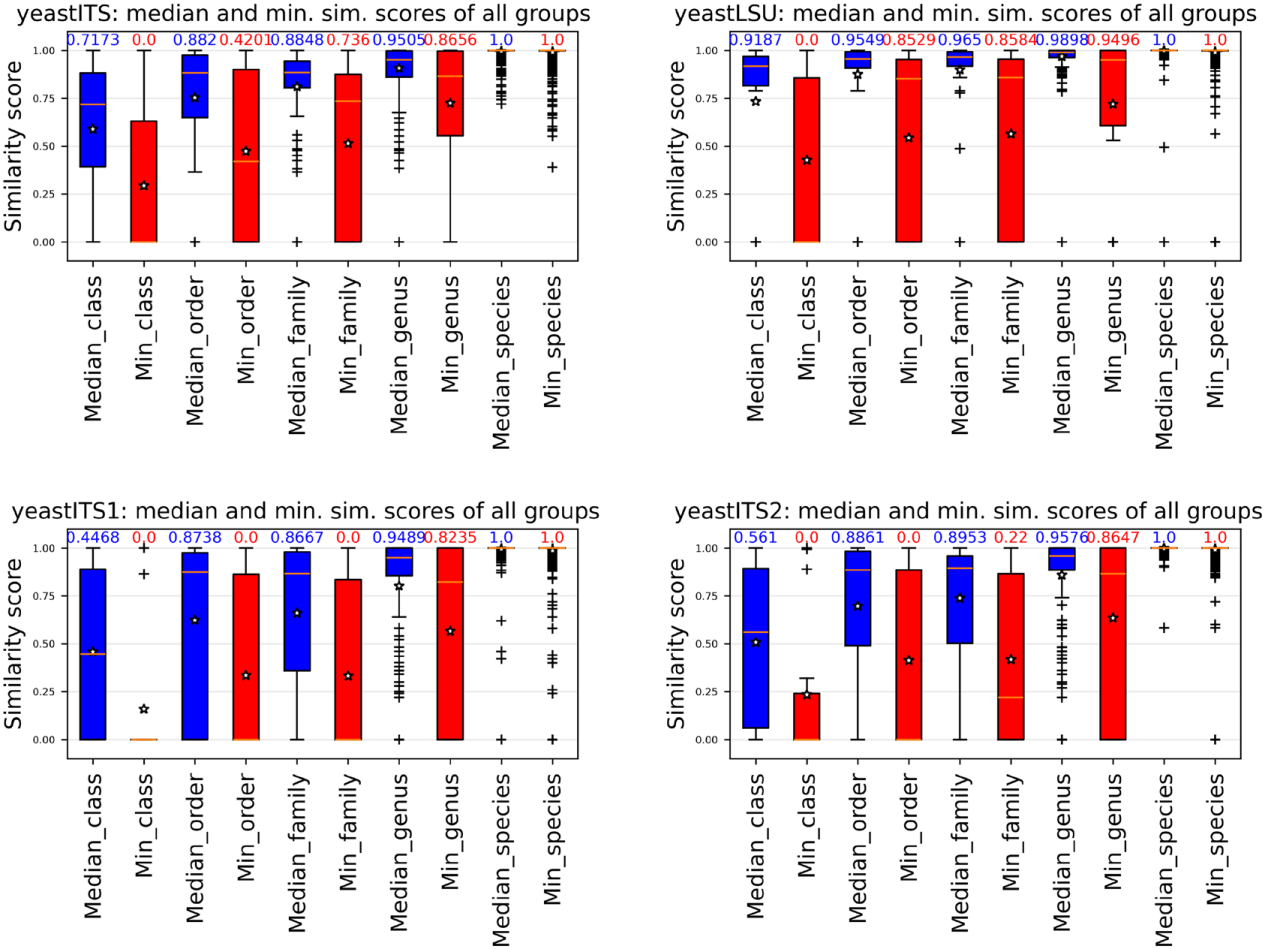
Variation in sequence similarity across yeast ITS and LSU datasets at different taxonomic levels. Median (blue) and minimum (red) similarity scores were computed for all groups in the yeastITS, yeastLSU, yeastITS1 and yeastITS2 datasets using dnabarcoder's variation command. The numbers above the bars indicate the median similarity values for each group. Stars represent the average similarity, while horizontal lines indicate the median similarity, highlighting differences in sequence variability across taxonomic levels.

Figure 3 displays the minimum (red) and median (blue) similarity values, which vary widely across all biomarkers. This variability confirms that a single cutoff threshold should not be applied uniformly to all yeast groups, as noted in previous studies (Vu et al. 2022). LSU showed the lowest within‐group variability and the highest median similarity, followed by ITS2, ITS and ITS1 (Table 2), suggesting that different biomarkers require distinct similarity cutoffs for yeast identification.

**TABLE 2:** Median and minimum sequence similarity within yeast taxonomic groups for ITS and LSU barcodes.

Level	Median of	yeast ITS	yeast ITS1	yeast ITS2	yeastLSU
Species	min. sim.	1	1	1	1
	median sim.	1	1	1	1
Genus	min. sim.	0.8656	0.8235	0.8647	0.9496
	median sim.	0.9505	0.9489	0.9576	0.9898
Family	min. sim.	0.7360	0	0.2200	0.8584
	median sim.	0.8848	0.8667	0.8953	0.9650
Order	min. sim.	0.4201	0	0	0.8529
	median sim.	0.8820	0.8738	0.8861	0.9549
Class	min. sim.	0	0	0	0
	median sim.	0.7173	0.4468	0.561	0.9187

*Note:* This table presents the median values of minimum (min. sim.) and median (median sim.) similarity scores for species, genus, family, order and class levels across the yeastITS, yeastITS1, yeastITS2 and yeastLSU datasets.

### 3.2 Indistinguishable Yeast Species by ITS, ITS1, ITS2 and LSU

As observed in previous studies (Abarenkov et al. 2016; Vu et al. 2016, 2019, 2022), certain yeast and filamentous fungal species cannot be distinguished using ITS and/or LSU. To approximately quantify these cases, we clustered the datasets using dnabarcoder's cluster command with a 100% similarity score. Species that merged into a group with another species were considered indistinguishable.

Table 3 shows the number and proportion of indistinguishable yeast species for each dataset, with separate values for *Ascomycota* and *Basidiomycota* across different barcoding regions: 119 (7.4%) with full‐length ITS, 301 (18.7%) with ITS1, 326 (20.8%) with ITS2 and 189 (11.4%) with LSU. Shorter barcodes, such as ITS1 and ITS2, resulted in substantially higher numbers of indistinguishable species compared to full‐length ITS and LSU. The full‐length ITS region provided the highest resolution, distinguishing the largest number of species. Across all datasets, basidiomycetes consistently exhibited a higher proportion of indistinguishable species than ascomycetes.

**TABLE 3:** Number and proportion of indistinguishable yeast species using ITS and LSU barcodes.

Dataset	Species no.	Indistinguishable species no.
yeastITS	1608	119 (7.4%)
yeastITS—*Ascomycota*	1022	60 (5.9%)
yeastITS—*Basidiomycota*	585	59 (10%)
yeastITS1	1606	301 (18.7%)
yeastITS1—*Ascomycota*	1020	160 (15.7%)
yeastITS1—*Basidiomycot*a	585	141 (24.1%)
yeastITS2	1568	326 (20.8%)
yeastITS2—*Ascomycota*	984	189 (19.2%)
yeastITS2—*Basidiomycota*	583	137 (23.5%)
yeastLSU	1663	189 (11.4%)
yeastLSU—*Ascomycota*	1065	104 (9.8%)
yeastLSU—*Basidiomycota*	573	85 (14.8%)

*Note:* This table shows the total number of species and the number (and percentage) of species that are indistinguishable for each barcode dataset (yeastITS, yeastITS1, yeastITS2 and yeastLSU), with separate values for *Ascomycota* and *Basidiomycota*.

### 3.3 Resolving Powers of ITS, ITS1, ITS2 and LSU for Yeast Species Identification

As discussed in Section 3.2, certain yeast species could not be distinguished using ITS, ITS1, ITS2, or LSU markers. This section compares the resolving powers of these markers for species identification across yeast genera and taxonomic groups of the yeastITS, yeastITS1, yeastITS2 and yeastLSU datasets. Resolving power was measured as the highest F‐measure for species delineation, calculated using dnabarcoder's predict command (Section 2.2). We assessed 38 yeast genera and 92 taxonomic groups where data for all four markers were available (see Appendix S5).

**FIGURE 4:**
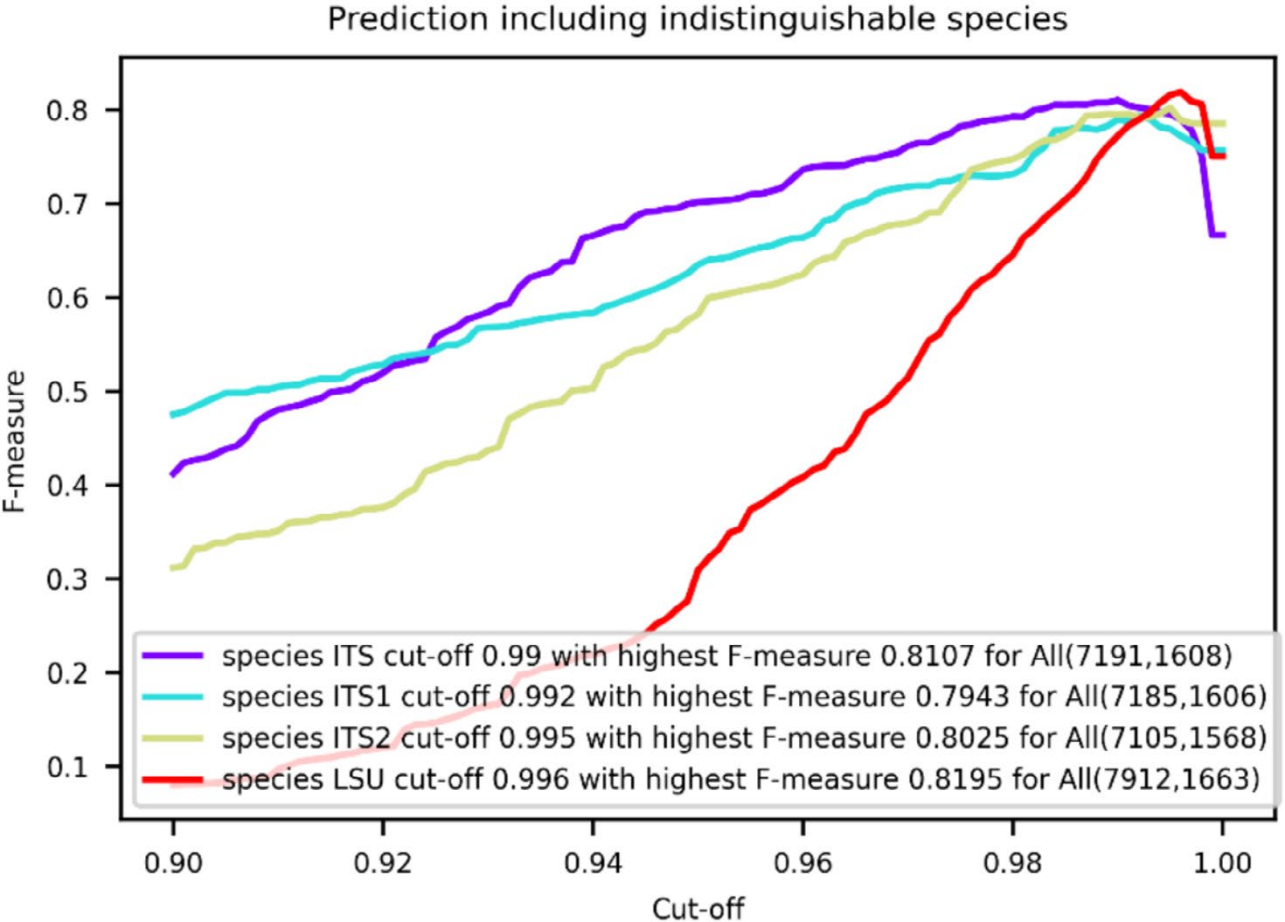
Predicted global similarity cutoffs and resolving powers for yeast species identification using ITS, ITS1, ITS2 and LSU barcodes. Prediction curves show global similarity cutoffs and resolving powers for the yeastITS, yeastITS1, yeastITS2 and yeastLSU datasets, including indistinguishable species. Numbers in parentheses indicate the total sequences and species within each dataset.

Figure 4 shows the prediction curves used to determine global similarity cutoffs and resolving powers for yeast species identification across the four yeast datasets, while Table 4 summarises the local cutoffs and resolving powers for each marker across yeast genera and the phyla *Ascomycota* and *Basidiomycota*. These results are also provided in Appendix S5.

**TABLE 4:** Predicted similarity cutoffs and resolving powers for yeast species identification in different genera using ITS, ITS1, ITS2 and LSU barcodes, including indistinguishable yeast species.

Clade	ITS_cutoff	ITS1_cutoff	ITS2_cutoff	LSU_cutoff	ITS_power	ITS1_power	ITS2_power	LSU_power
*Apiotrichum*	0.997	0.992	0.994	0.994	0.8736	0.7055	0.9186	0.9298
*Blastobotrys*	0.993	0.981	0.977	0.986	0.9195	0.9046	0.9046	0.8534
*Candida*	0.988	0.985	0.986	0.995	0.9468	0.9261	0.9224	0.9460
*Candidozyma*	0.919	0.843	0.985	0.978	0.8888	0.9705	0.9900	0.8995
*Cryptococcus*	0.999	0.992	0.996	0.999	0.7555	0.5001	0.6117	0.7078
*Cutaneotrichosporon*	0.989	0.992	0.989	0.995	0.8947	0.8578	0.8838	0.8113
*Cyberlindnera*	0.983	0.989	0.990	0.993	0.7998	0.8014	0.7921	0.8142
*Cystobasidium*	0.990	0.988	0.986	0.997	0.8953	0.8343	0.9012	0.8899
*Debaryomyces*	0.995	0.991	0.995	0.999	0.6737	0.6732	0.617	0.6738
*Dioszegia*	0.993	0.985	0.990	0.999	0.9824	0.9609	0.8838	0.8711
*Filobasidium*	0.997	0.991	0.996	0.998	0.9128	0.8245	0.9309	0.8302
*Hanseniaspora*	0.995	0.993	0.992	0.998	0.847	0.7871	0.9105	0.8709
*Kodamaea*	0.937	0.929	0.987	0.991	0.7906	0.9815	0.9804	1
*Lachancea*	0.986	0.992	0.977	0.992	0.9731	0.9221	0.9792	0.9743
*Lipomyces*	0.980	0.975	0.963	0.999	0.8912	0.7854	0.8887	0.8145
*Malassezia*	0.967	0.927	0.936	0.991	0.9918	0.9497	0.9896	0.9893
*Metschnikowia*	0.926	0.937	0.965	0.995	0.7767	0.8766	0.7585	0.8608
*Naganishia*	0.993	0.992	0.996	0.996	0.7711	0.7636	0.6953	0.8681
*Nakazawaea*	0.996	0.983	0.986	0.998	0.9107	0.803	0.9024	0.8439
*Ogataea*	0.987	0.984	0.985	0.997	0.9466	0.7961	0.9136	0.8282
*Papiliotrema*	0.982	0.971	0.977	0.991	0.9150	0.8483	0.915	0.8819
*Pichia*	0.966	0.965	0.964	0.990	0.9188	0.9294	0.881	0.9284
*Rhodosporidiobolus*	0.986	0.994	0.981	0.996	0.9854	0.9559	1	0.9329
*Rhodotorula*	0.990	0.994	0.994	0.996	0.9245	0.7076	0.8856	0.8881
*Saccharomyces*	0.993	0.995	0.995	0.999	0.5916	0.6100	0.4928	0.5812
*Saccharomycopsis*	0.974	0.953	0.988	0.992	0.9900	1	0.9544	0.9613
*Scheffersomyces*	0.997	0.995	0.995	0.996	0.8520	0.8419	0.7673	0.7583
*Schwanniomyces*	0.996	0.996	0.995	0.996	0.6731	0.7318	0.6823	0.6980
*Sporobolomyces*	0.988	0.987	0.991	0.994	0.8989	0.9312	0.9065	0.8669
*Starmerella*	0.970	0.965	0.987	0.986	0.9769	0.9780	0.9255	0.9927
*Sugiyamaella*	0.910	0.969	0.987	0.994	1	1	1	1
*Suhomyces*	0.957	0.942	0.966	0.993	1	1	1	0.9900
*Teunomyces*	0.972	0.952	0.982	0.997	0.9611	0.9864	0.9181	0.9899
*Trichosporon*	0.999	0.992	0.995	0.999	0.7764	0.7113	0.7119	0.8174
*Vishniacozyma*	0.996	0.986	0.994	0.993	0.8443	0.7418	0.8211	0.9234
*Wickerhamiella*	0.975	0.970	0.987	0.984	0.9355	0.9491	0.9227	0.9718
*Wickerhamomyces*	0.950	0.983	0.953	0.993	0.9887	0.8867	0.9681	0.9707
*Yamadazyma*	0.987	0.980	0.980	0.993	1	0.9825	0.9825	0.9837

*Note:* This table presents predicted similarity cutoffs and resolving powers for species identification in different genera of the yeastITS, yeastITS1, yeastITS2 and yeastLSU datasets. Only genera with more than 30 sequences and at least 5 species were included, and genera dominated by a single species (> 70% of the sequences) were excluded to improve prediction accuracy. Pink shading highlights local similarity cutoffs that are greater than or equal to the corresponding global cutoff, while green and orange shading indicate the highest and lowest resolving powers, respectively, among the biomarkers for species identification within each genus. The composition and membership of the genera used in this analysis are provided in Appendix S1.

Global resolving powers of ITS, ITS1, ITS2 and LSU were 0.811, 0.794, 0.803 and 0.820, respectively. Local resolving powers were generally higher than the corresponding global resolving powers for most taxonomic groups—76% for ITS, 71% for ITS1, 76% for ITS2 and 81% for LSU—indicating that the four biomarkers performed better locally.

Overall, local resolving powers varied across taxonomic groups and biomarkers. ITS was the most effective marker for yeast species identification, showing the highest resolving power in 42% of genera and 42% of all taxonomic groups, followed by LSU (29% and 29%), ITS2 (24% and 21%) and ITS1 (21% and 15%). ITS outperformed LSU in 55% of genera and 57% of all taxonomic groups, with particularly high resolving power in *Blastobotrys*, *Dioszegia*, *Lipomyces* and *Nakazawaea*, whereas LSU outperformed ITS in *Kodamaea*, *Metschnikowia*, *Naganishia* and *Vishniacozyma*. These indicate that while ITS generally offers greater resolution, the two markers are complementary for yeast species identification.

The shorter regions ITS1 and ITS2 consistently showed lower resolving power than the longer ITS and LSU regions. ITS1 had the lowest resolving power most frequently, followed by ITS2, LSU and ITS. ITS2 generally outperformed ITS1 (61% of all taxonomic groups), although ITS1 showed higher resolving power in certain genera, such as *Dioszegia*, *Metschnikowia*, *Pichia* and *Teunomyces*. Compared to the full‐length ITS and LSU regions, ITS1 and ITS2 showed inferior performance across most genera and taxonomic groups, with their resolving power frequently falling below 0.8, highlighting the superior discriminatory power of ITS and LSU.

Although ITS and LSU generally perform well for yeast species identification, some genera—such as *Cryptococcus*, *Debaryomyces*, *Saccharomyces* and *Schwanniomyces*—show consistently low resolving power across all markers (ITS, ITS1, ITS2, LSU). These genera contain species known to be indistinguishable by these barcode regions (Naumova et al. 2005; Groenewald et al. 2008; Meyer et al. 2009; Nguyen et al. 2009; Hagen et al. 2015; Nguyen and Boekhout 2017; Sugita et al. 2002). As a result, predicted cutoffs for these groups are often high (above the global threshold; see pink highlights in Table 4), reflecting attempts to artificially separate indistinguishable species. The use of these inflated cutoffs risks false positives and may misrepresent species boundaries. For more accurate thresholds, indistinguishable species should be excluded prior to analysis, as recommended by Vu et al. (2022).

### 3.4 Similarity Cutoffs of ITS, ITS1, ITS2 and LSU for Yeast Species Identification

This section presents predicted similarity cutoffs for the ITS, ITS1, ITS2 and LSU markers in yeast species identification based on the yeastITS, yeastITS1, yeastITS2 and yeastLSU datasets. Following Section 3.3, indistinguishable species were excluded by retaining one representative per complex using dnabarcoder's remove command. We assessed 38 yeast genera and 90 taxonomic groups where data for all four markers were available (see Appendix S5).

**FIGURE 5:**
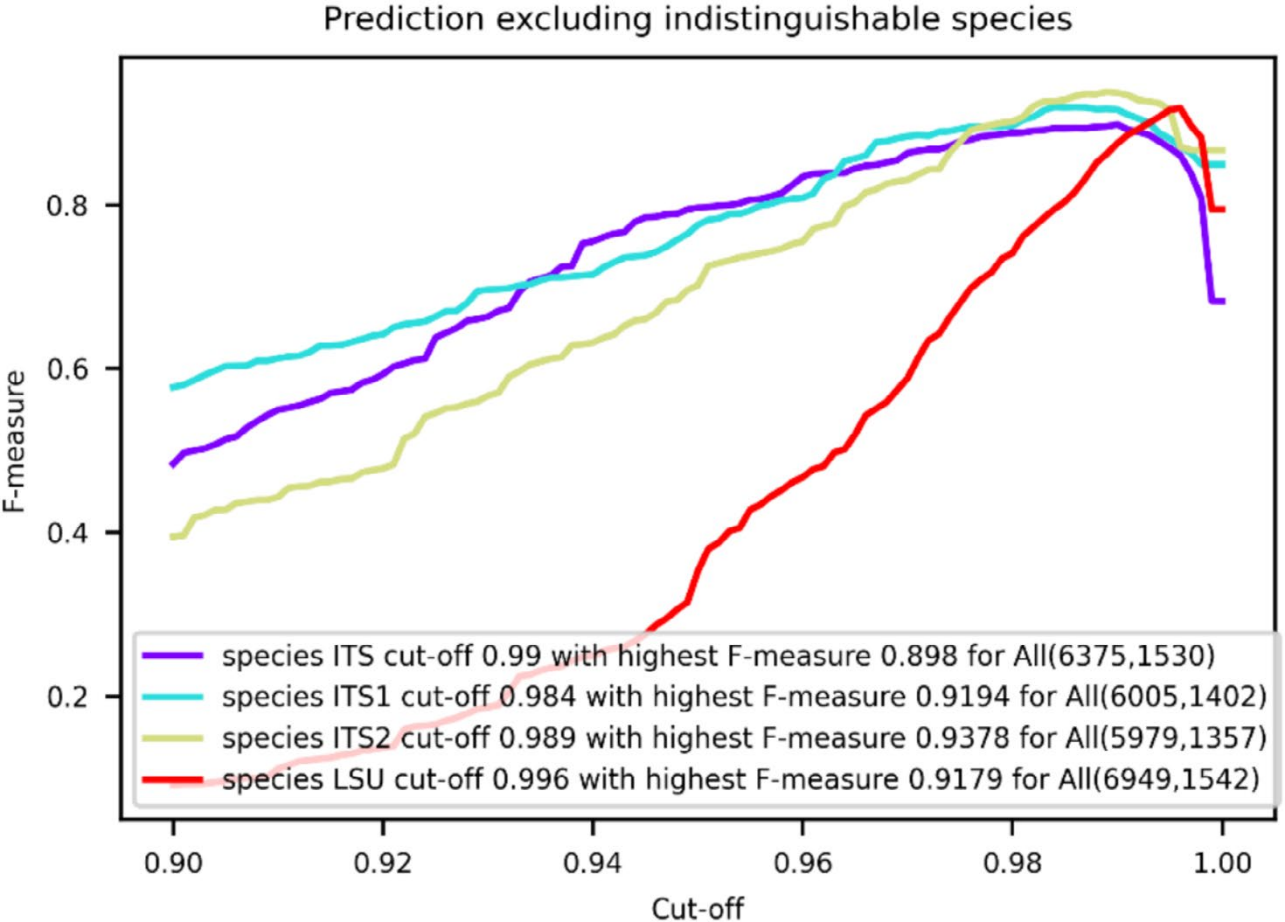
Predicted global similarity cutoffs and resolving powers for yeast species identification using ITS, ITS1, ITS2 and LSU barcodes, excluding indistinguishable species. Prediction curves show global similarity cutoffs and resolving powers for the yeastITS, yeastITS1, yeastITS2 and yeastLSU datasets, with indistinguishable species excluded. Numbers in parentheses indicate the total sequences and species within each dataset.

Figure 5 shows the prediction curves used to determine global cutoffs and resolving powers for each dataset, while Table 5 summarises local values across yeast genera and the phyla *Ascomycota* and *Basidiomycota*.

**TABLE 5:** Predicted similarity cutoffs and resolving powers for species identification in different yeast genera using ITS, ITS1, ITS2 and LSU barcodes, excluding indistinguishable species.

Genera	ITS_cutoff	ITS1_cutoff	ITS2_cutoff	LSU_cutoff	ITS_power	ITS1_power	ITS2_power	LSU_power
*Apiotrichum*	0.997	0.992	0.994	0.994	0.8756	0.9619	0.9825	0.9298
*Blastobotrys*	0.993	0.981	0.977	0.986	0.9722	1	0.9743	0.9254
*Candida*	0.988	0.985	0.991	0.995	0.9606	0.9468	0.9502	0.9549
*Candidozyma*	0.919	0.843	0.985	0.978	0.8888	0.9773	0.9900	1
*Cutaneotrichosporon*	0.989	0.990	0.989	0.995	0.9858	1	0.9756	0.9073
*Cyberlindnera*	0.983	0.989	0.990	0.993	0.8689	0.9003	0.9132	0.9151
*Cystobasidium*	0.990	0.988	0.986	0.991	0.9575	0.9288	0.9694	0.9553
*Cystofilobasidium*	0.972	0.981	0.981	0.992	1	0.9573	0.9838	1
*Filobasidium*	0.997	0.990	0.996	0.998	0.9128	1	1	0.9630
*Hanseniaspora*	0.995	0.993	0.992	0.998	0.8470	0.8307	0.9495	0.9120
*Hyphopichia*	0.914	0.887	0.889	0.989	1	1	0.9714	0.9875
*Kluyveromyces*	0.989	0.974	0.993	0.999	0.9826	0.9826	0.9750	0.9144
*Kodamaea*	0.937	0.929	0.987	0.991	0.7906	0.9815	0.9804	1
*Lachancea*	0.986	0.971	0.977	0.992	0.9731	0.9666	0.9945	0.9743
*Lipomyces*	0.980	0.975	0.963	0.999	0.9014	0.8494	0.9026	0.8118
*Malassezia*	0.967	0.927	0.936	0.991	0.9918	0.9678	0.9896	0.9893
*Metschnikowia*	0.926	0.937	0.965	0.995	0.7767	0.9183	0.9141	0.8608
*Naganishia*	0.993	0.992	0.987	0.996	0.9401	0.9817	0.9195	0.9200
*Ogataea*	0.987	0.982	0.985	0.997	0.9611	0.9587	0.9513	0.8282
*Papiliotrema*	0.982	0.961	0.977	0.991	0.9150	0.9780	0.9321	0.8819
*Pichia*	0.966	0.947	0.934	0.990	0.9247	0.9780	0.9768	0.9825
*Rhodosporidiobolus*	0.986	0.994	0.981	0.996	0.9854	0.9559	1	0.9217
*Rhodotorula*	0.990	0.993	0.982	0.996	0.9579	0.9597	0.9847	0.9801
*Saccharomycopsis*	0.974	0.953	0.988	0.992	0.9900	1	0.9544	0.9613
*Scheffersomyces*	0.997	0.995	0.995	0.996	0.8520	0.9023	0.8837	0.7966
*Sporobolomyces*	0.996	0.987	0.991	0.993	0.9366	0.9856	0.9681	0.9804
*Starmerella*	0.970	0.965	0.987	0.986	0.9769	0.9780	0.9728	0.9927
*Sugiyamaella*	0.910	0.969	0.987	0.994	1	1	1	1
*Suhomyces*	0.957	0.942	0.966	0.991	1	1	1	0.9940
*Teunomyces*	0.972	0.952	0.982	0.997	0.9611	0.9864	1	0.9899
*Torulaspora*	0.980	0.951	0.982	0.996	0.9037	0.9086	0.9715	0.9131
*Trichosporon*	0.998	0.992	0.995	0.997	0.9261	0.9510	0.9307	0.9278
*Vishniacozyma*	0.996	0.986	0.964	0.993	0.8443	0.9052	0.9847	0.9336
*Wickerhamiella*	0.975	0.970	0.987	0.984	0.9355	0.9491	0.9901	0.9718
*Wickerhamomyces*	0.950	0.976	0.953	0.993	0.9887	0.8947	0.9681	0.9707
*Yamadazyma*	0.987	0.969	0.980	0.993	1	0.9815	0.9825	0.9837
*Zygosaccharomyces*	0.923	0.914	0.960	0.982	1	1	1	0.9720
*Ascomycota*	0.984	0.984	0.986	0.996	0.8967	0.9168	0.937	0.9121
*Basidiomycota*	0.990	0.990	0.989	0.996	0.9108	0.9361	0.9468	0.9290
*All*	0.990	0.984	0.989	0.996	0.8980	0.9194	0.9378	0.9179

*Note:* This table presents predicted similarity cutoffs and resolving powers for yeast identification across different genera for the phyla *Ascomycota* and *Basidiomycota* and for the full yeastITS, yeastITS1, yeastITS2 and yeastLSU datasets, with indistinguishable species excluded. Only genera with more than 30 sequences and at least 5 species were included, and genera dominated by a single group (> 70%) were excluded to improve prediction accuracy. Pink and blue shading highlight the highest and lowest similarity cutoffs, respectively, while green shading indicates high resolving powers (> 0.9) among the markers for species identification within each genus. The composition and membership of the taxonomic groups used in this analysis are provided in Appendix S1.

Global similarity cutoffs for yeast species identification were 0.99 (ITS), 0.984 (ITS1), 0.989 (ITS2) and 0.996 (LSU) (Figure 5). Excluding indistinguishable species reduced global cutoffs for ITS1 and ITS2 (from 0.992 to 0.984 and 0.995 to 0.989, respectively) and decreased the proportion of all taxonomic groups with cutoffs above 0.99—from 38% to 16% (ITS), 47% to 25% (ITS1) and 42% to 15% (ITS2).

After exclusion, all four markers showed high resolving power: over 68%, 88%, 96% and 93% of yeast taxonomic groups exceeded 0.9 for ITS, ITS1, ITS2 and LSU, respectively (Table 5; Appendix S5). This confirms that, beyond the known indistinguishable species (Table 3), these markers strongly discriminate the remaining yeast species, which represent 92.6%, 81.3%, 79.2% and 88.6% of the yeastITS, yeastITS1, yeastITS2 and yeastLSU datasets, respectively.

Predicted cutoffs varied across taxonomic groups and markers, reflecting sequence divergence and taxonomic complexity. LSU had the highest cutoffs—above 0.99 in 87% of genera and 95% of higher taxa. ITS1 was the most variable, showing the lowest cutoffs in 58.5% of genera and 46.6% of higher taxa. Between ITS and ITS2, ITS was more variable, with lower cutoffs in about 40% of groups.

ITS1 displayed lower cutoffs than ITS2 in several genera, including *Candida*, *Candidozyma*, *Kodamaea*, *Malassezia*, *Metschnikowia* and *Saccharomycopsis*, but this did not consistently translate into higher resolving power. Only *Metschnikowia* showed better performance for ITS1 (0.8766 vs. 0.7585).

Additionally, ITS and ITS1 cutoffs were also generally lower in *Ascomycota* than in *Basidiomycota*. Overall, the results highlight the need for taxon‐specific, marker‐dependent thresholds to improve yeast species identification accuracy.

### 3.5 Similarity Cutoffs and Resolving Powers of ITS, ITS1, ITS2 and LSU for Yeast Identification at the Genus and Higher Taxonomic Levels

This section examines the resolving powers and similarity cutoffs of ITS, ITS1, ITS2 and LSU for yeast identification at the genus and higher taxonomic levels using the yeastITS, yeastITS1, yeastITS2 and yeastLSU datasets. A total of 42 taxonomic groups with data for all four markers were analysed (Table 6).

**TABLE 6:** Predicted similarity cutoffs and resolving powers for genus‐ and higher‐level sequence identification across yeast clades using ITS and LSU barcodes.

Rank	Taxonomic group	ITS_cutoff	ITS1_cutoff	ITS2_cutoff	LSU_cutoff	ITS_power	ITS1_power	ITS2_power	LSU_power
Genus	*All*	0.930	0.842	0.967	0.976	0.7026	0.6452	0.6664	0.6915
Genus	*Cystofilobasidiaceae*	0.878	0.902	0.910	0.953	1	1	1	1
Genus	*Debaryomycetaceae*	0.908	0.984	0.700	0.988	0.5417	0.4981	0.5368	0.5030
Genus	*Metschnikowiaceae*	0.861	0.700	0.700	0.891	0.5762	0.4715	0.7887	0.6727
Genus	*Pichiaceae*	0.848	0.852	0.701	0.921	0.9091	0.5490	0.6367	0.8798
Genus	*Saccharomycetaceae*	0.894	0.910	0.964	0.983	0.9323	0.9105	0.8608	0.9083
Genus	*Trichomonascaceae*	0.829	0.700	0.700	0.931	0.7198	0.6362	0.6802	0.6369
Genus	*Trichosporonaceae*	0.932	0.944	0.951	0.967	0.9208	0.9070	0.8475	0.9412
Genus	*Agaricostilbales*	0.845	0.700	0.920	0.915	0.8571	0.8896	0.7733	0.9297
Genus	*Cystofilobasidiales*	0.879	0.902	0.925	0.953	0.9905	0.9028	0.9797	0.9912
Genus	*Dipodascales*	0.858	0.700	0.700	0.932	0.7511	0.6590	0.7677	0.7512
Genus	*Filobasidiales*	0.901	0.921	0.962	0.972	0.9598	0.8665	0.8552	0.9534
Genus	*Pichiales*	0.848	0.852	0.701	0.921	0.9091	0.549	0.6367	0.8798
Genus	*Saccharomycetales*	0.894	0.910	0.964	0.983	0.9323	0.9107	0.8621	0.9081
Genus	*Serinales*	0.908	0.843	0.700	0.961	0.5303	0.4839	0.5694	0.4988
Genus	*Tremellales*	0.929	0.905	0.930	0.969	0.8924	0.9126	0.9044	0.9198
Genus	*Trichosporonales*	0.932	0.944	0.951	0.967	0.9218	0.9039	0.8452	0.9419
Genus	*Ustilaginales*	0.923	0.976	0.952	0.992	0.8065	0.7243	0.8018	0.7609
Genus	*Agaricostilbomycetes*	0.845	0.700	0.920	0.915	0.8571	0.8896	0.7733	0.9297
Genus	*Cystobasidiomycetes*	0.957	0.942	0.947	0.964	0.9127	0.9345	0.8334	0.9712
Genus	*Dipodascomycetes*	0.858	0.700	0.700	0.932	0.7511	0.6590	0.7677	0.7512
Genus	*Microbotryomycetes*	0.924	0.961	0.959	0.976	0.8933	0.8186	0.8853	0.7617
Genus	*Pichiomycetes*	0.905	0.852	0.700	0.971	0.5894	0.4757	0.5207	0.5290
Genus	*Saccharomycetes*	0.860	0.929	0.966	0.983	0.8671	0.8446	0.8050	0.8239
Genus	*Tremellomycetes*	0.930	0.921	0.946	0.969	0.8872	0.8646	0.8675	0.9247
Genus	*Ustilaginomycetes*	0.923	0.976	0.952	0.992	0.8125	0.7330	0.8080	0.7678
Genus	*Ascomycota*	0.905	0.841	0.975	0.979	0.6600	0.5894	0.5814	0.6191
Genus	*Basidiomycota*	0.930	0.951	0.958	0.973	0.8638	0.8121	0.8364	0.8476
Family	*All*	0.897	0.843	0.941	0.958	0.6850	0.6290	0.5928	0.7213
Family	*Agaricostilbales*	0.807	0.600	0.843	0.899	0.8708	0.7577	0.7070	0.9132
Family	*Tremellales*	0.915	0.905	0.929	0.963	0.8735	0.8872	0.8873	0.8956
Family	*Agaricostilbomycetes*	0.807	0.600	0.843	0.899	0.8708	0.7577	0.7070	0.9132
Family	*Tremellomycetes*	0.909	0.905	0.910	0.959	0.8786	0.8967	0.8442	0.8557
Family	*Ascomycota*	0.844	0.601	0.703	0.958	0.6937	0.6392	0.5119	0.6698
Family	*Basidiomycota*	0.921	0.912	0.912	0.959	0.8402	0.8929	0.8129	0.8247
Order	*All*	0.895	0.843	0.941	0.955	0.6813	0.5860	0.5499	0.6995
Order	*Tremellomycetes*	0.896	0.888	0.902	0.951	0.8091	0.7075	0.7492	0.8051
Order	*Ascomycota*	0.844	0.601	0.703	0.955	0.7225	0.6306	0.4876	0.6759
Order	*Basidiomycota*	0.896	0.842	0.907	0.951	0.8124	0.7696	0.7651	0.7546
Class	*All*	0.871	0.723	0.941	0.935	0.6756	0.6189	0.4766	0.6989
Class	*Ascomycota*	0.809	0.583	0.521	0.955	0.7706	0.6478	0.5807	0.6507
Class	*Basidiomycota*	0.847	0.685	0.782	0.926	0.9550	0.8525	0.7308	0.9757

*Note:* This table presents predicted similarity cutoffs and resolving powers for ITS, ITS1, ITS2 and LSU markers across yeast taxonomic groups at genus and higher taxonomic levels. Only taxa with more than 30 sequences and at least 5 groups were included, and taxa dominated by a single group (> 70%) were excluded to improve prediction accuracy. Pink and blue shading indicate the highest and lowest similarity cutoffs, respectively, while green and orange shading highlight the highest and lowest resolving powers observed among the markers for sequence identification within each taxonomic group. The composition and membership of these taxonomic groups used in the analysis are provided in Appendix S1.

#### 3.5.1 Resolving Powers of ITS, ITS1, ITS2 and LSU at Genus and Higher Taxonomic Levels

**FIGURE 6:**
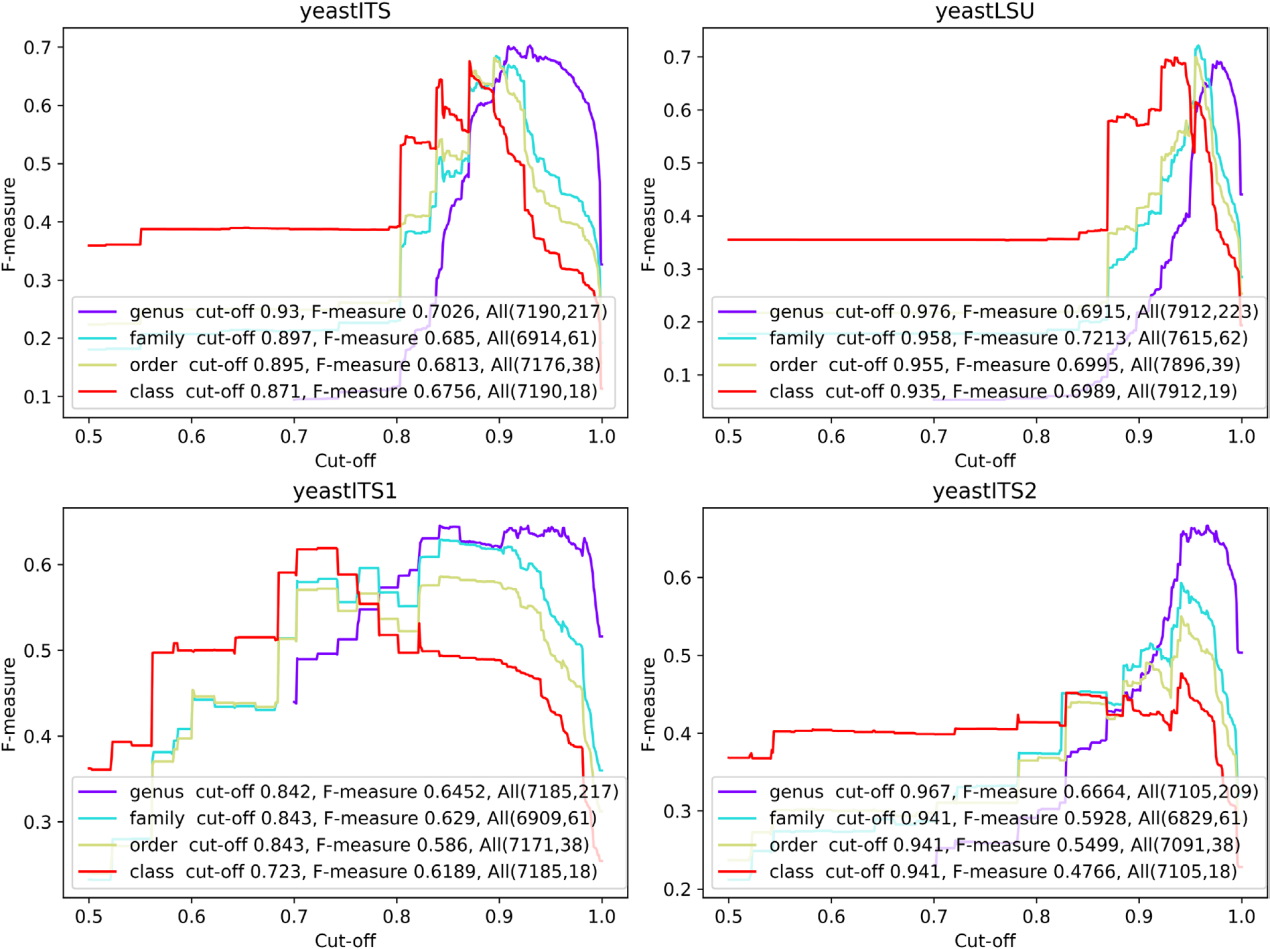
Predicted global similarity cutoffs for genus‐ and higher‐level sequence identification in yeast ITS and LSU datasets. This figure displays predicted global similarity cutoffs for genus, family, order and class levels for the yeastITS, yeastLSU, yeastITS1 and yeastITS2 datasets. Numbers in parentheses indicate the total number of sequences and groups at each taxonomic level.

Figure 6 shows prediction curves for global similarity cutoffs and resolving powers for yeast identification at genus and higher taxonomic levels. Multiple peaks were observed, likely reflecting distinct divergence patterns between *Ascomycota* and *Basidiomycota* (Figure 2), suggesting that variable similarity cutoffs are needed for accurate identification. Global resolving powers obtained by all biomarkers were generally low (< 0.73) and decreased with taxonomic rank, highlighting the limitation of a single static cutoff above species level.

Table 6 summarises local similarity cutoffs and resolving powers of the biomarkers, showing broad variation across taxonomic groups and ranks, particularly for ITS and LSU. All four markers showed higher resolving power in *Basidiomycota* than in *Ascomycota*. On average, local genus‐level resolving powers were 0.88 (ITS), 0.81 (ITS1), 0.81 (ITS2) and 0.87 (LSU). At higher ranks, ITS2 performed poorly in *Ascomycota* (< 0.59). Despite this, all four markers generally performed better locally (87% of all taxonomic groups for ITS, 79% for ITS1, 82% for ITS2 and 76.3% for LSU), reinforcing the need for taxon‐specific similarity cutoffs to ensure robust yeast taxonomic resolution.

Several taxa—*Debaryomycetaceae*, *Metschnikowiaceae*, *Serinales*, *Trichomonascaceae* and *Dipodascales*—showed low genus‐level resolution (< 0.76) across all markers, largely due to poorly resolved genera such as *Candida*, *Metschnikowia* and *Wickerhamiella*, suggesting a need for taxonomic revision of these lineages.

Full‐length ITS demonstrated the highest taxonomic resolution, outperforming other markers in 50% of groups, followed by LSU (38%), ITS2 (12%) and ITS1 (5%). Longer regions (ITS and LSU) surpassed shorter ones (ITS1 and ITS2) in over 75% of groups. For example, in *Pichiaceae*, ITS and LSU achieved resolving powers of 0.9091 and 0.8798, compared to 0.549 (ITS1) and 0.6367 (ITS2). ITS slightly outperformed LSU in 54% of groups, particularly in *Trichomonascaceae* and *Microbotryomycetes*, while LSU performed better in *Metschnikowiaceae* and *Agaricostilbales*. Across *Ascomycota*, ITS consistently surpassed LSU at all taxonomic ranks. Among shorter regions, ITS2 exceeded ITS1 in 57% of genera, whereas ITS1 performed better at higher ranks (86% of families, orders and classes).

#### 3.5.2 Similarity Cutoffs of ITS, ITS1, ITS2 and LSU at Genus and Higher Taxonomic Levels

As shown in Figure 6 and Table 6, LSU exhibited the highest global similarity cutoffs across taxonomic ranks, followed by ITS2, ITS and ITS1. Consistent with species‐level identification, predicted local cutoffs for yeast identification at genus and higher taxonomic levels varied widely across taxa and markers. *Ascomycota* required lower similarity cutoffs than *Basidiomycota* for ITS and ITS1. LSU and ITS1 were the most conserved and most variable regions, respectively, with the highest and lowest cutoffs observed in 39 and 24 of 42 taxonomic groups (pink and blue in Table 6). Between ITS and ITS2, full‐length ITS was more variable, showing lower cutoffs in 64% (27/42) of groups. These results again underscore the importance of marker‐ and taxon‐specific similarity cutoffs for optimising yeast taxonomic resolution.

### 3.6 Recent Name Changes Improve Yeast Taxonomic Classification

One of the main challenges in fungal and yeast identification lies in the continuously evolving taxonomy, which strives to balance consistency, accuracy and compliance with advances in scientific understanding. This leads to continuous taxonomic revisions and renaming to better reflect true evolutionary relationships. Over the past year, many new yeast genera have been introduced, and numerous species reclassified, particularly in the orders *Saccharomycetales* and *Serinales* (Liu, Hu, Yurkov, et al. 2024; Liu, Hu, Zhao, et al. 2024; Zhu et al. 2024). The number of genera increased from 26 to 41 in *Saccharomycetales* and from 27 to 40 in *Serinales*, with 41 and 55 species, respectively, reassigned as new combinations to newly described genera. One notable example is *Candida auris*, an emerging multidrug‐resistant pathogen, which has been reclassified into a different genus and renamed as *Candidozyma auris* (Liu, Hu, Yurkov, et al. 2024). The question is: how do these name changes improve the resolving power of ITS and LSU for yeast identification?

To address this question, we calculated the resolving power of ITS and LSU barcodes for genus‐level identification within the orders *Saccharomycetales* and *Serinales*, the classes *Saccharomycetes* and *Pichiomycetes*, and the phylum *Ascomycota*—the groups most affected by recent taxonomic changes. Analyses were based on classifications from 2023 (Groenewald et al. 2023) and 2024 (Liu, Hu, Zhao, et al. 2024). The results are summarised in Table 7.

**TABLE 7:** Resolving powers (confidence *F*‐measures) of ITS and LSU for genus identification in the orders *Saccharomycetales* and *Serinales* and their higher taxonomic levels in 2024, compared to 2023.

*F*‐measure	yeastITS (2023)	yeastITS (2024)	yeastLSU (2023)	yeastLSU (2024)
*Saccharomycetales*	0.9207	**0.9323**	0.8633	**0.9081**
*Serinales*	0.5110	**0.5303**	0.4719	**0.4988**
*Saccharomycetes*	0.8550	**0.8671**	0.7924	**0.8239**
*Pichiomycetes*	0.5691	**0.5894**	0.4961	**0.5290**
*Ascomycota*	0.6398	**0.6600**	0.5959	**0.6191**
All	0.6877	**0.7026**	0.6772	**0.6915**

*Note:* The higher confidence value for each biomarker between the 2 years is shown in bold.

The results show increases in the resolving power of ITS and LSU for genus identification following recent taxonomic updates: ~1% (ITS) and 3% (LSU) in *Saccharomycetales*/*Saccharomycetes*; 2% (ITS) and 3% (LSU) in *Serinales*/*Pichiomycetes*; 2% (ITS) and 2% (LSU) in *Ascomycota*; and 1.5% (ITS) and 1.4% (LSU) across the full dataset.

**FIGURE 7:**
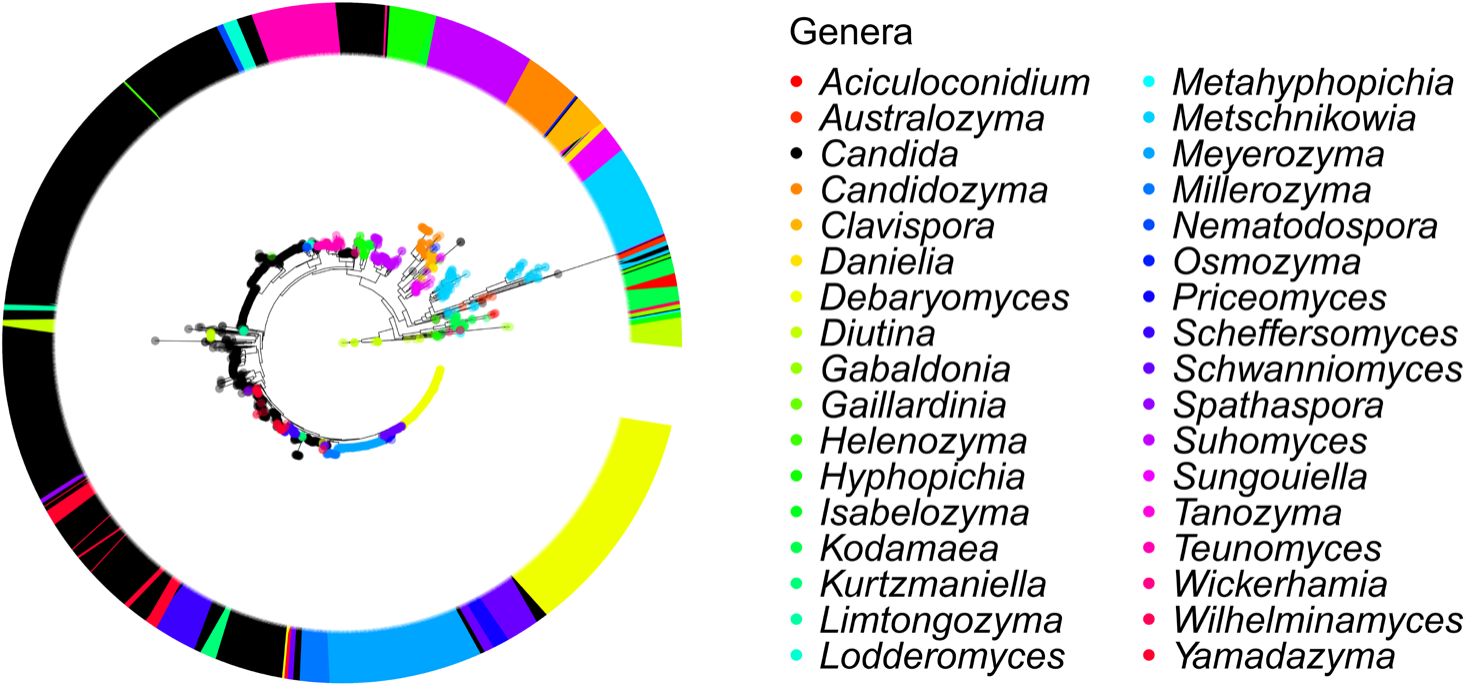
Phylogenetic tree of the *Serinales* based on 1835 yeast ITS barcodes. This tree illustrates the evolutionary relationships among *Serinales* genera, highlighting the dominance of the genus *Candida* (shown in black), whose ITS sequences are broadly distributed across the phylogeny, highlighting the continuing need for genus‐level reclassification within the order. Detailed ITS and LSU phylogenetic trees for this order are provided in Appendices S7 and S8.

These findings indicate that recent genus‐level taxonomic revisions have improved the concordance between taxonomy and sequence‐based groupings, as reflected by the *F*‐measure. Overall resolving power remains limited, particularly within the order *Serinales*, highlighting a continuing need for reclassification in this order. This limitation is evident in the *Serinales* phylogenetic tree (Figure 7), constructed from 1835 yeast ITS barcodes, where the genus *Candida* (in black) remains dominant and its ITS sequences are broadly distributed across the tree. Note that sequence similarity is only one aspect of taxonomic validity; metrics such as the *F*‐measure complement—but do not replace—other biological criteria, including morphological, ecological and physiological data, which remain essential for fully assessing the validity and biological coherence of newly circumscribed taxa.

### 3.7 Improving Identification of the Human Microbiome Dataset Using CBS Barcodes

To demonstrate the value of curated reference databases with updated taxonomy and tailored similarity thresholds, we reanalyzed 317 human gut microbiome samples from 147 healthy individuals (Nash et al. 2017). In that study, the authors used a similarity‐based operational taxonomic units (OTUs; Blaxter et al. 2005) approach for data analysis. Specifically, after merging sequencing pairs, sequences were clustered into OTUs using a 97% similarity cutoff. Following the removal of chimeras, 1954 representative OTU sequences remained. These sequences were aligned against the UNITE database (Kõljalg et al. 2005), and unmapped OTUs were manually analysed using BLAST (Altschul et al. 1997). In total, 701 fungal sequences belonging to 247 genera were detected.

Instead of traditional OTU‐based methods, which may reduce taxonomic resolution due to fixed similarity cutoffs (Vu et al. 2022), we applied DADA2 (Callahan et al. 2016, 2017) to generate a dataset containing all unique ITS2 sequences. As reference datasets, we used filfungalITS2 and yeastITS2, comprising a total of 18,778 ITS2 barcodes, instead of UNITE. Table 8 summarises the bioinformatics methods, reference datasets, unique ITS2 sequences obtained from gut samples, and genus‐ and species‐level identifications from both studies.

**TABLE 8:** Comparison of bioinformatics approaches, reference datasets and sequence classification outcomes between Nash et al. (2017) and this study.

	Nash et al. (2017)	This study
Method to obtain representative sequences	The OTU approach	The ASV approach (DADA2)
Reference dataset	UNITE database	filfungalITS2 and yeastITS2 (18,779 ITS2 barcodes)
Classification method	BLASTn + manual check	dnabarcoder
Number of representative sequences	1954	2004
Number of fungal sequences identified at the genus level	704	795
Number of genera	234 (however many named genera contain insufficient information such as ‘Fungi sp.’, ‘Ascomycota sp.’, etc.)	192
Number of fungal sequences identified at the species level	N/A	654
Number of species	N/A	317

*Note:* This table summarises the methods used to generate representative ITS2 sequences, the reference databases employed, and the resulting numbers of sequences classified at the genus and species levels. Key differences include the OTU‐based approach used by Nash et al. (2017) versus the ASV‐based approach (DADA2) employed in this study; the use of the UNITE database versus the filfungalITS2 and yeastITS2 datasets (a total of 18,778 ITS2 barcodes); and the classification methods (BLASTn with manual curation versus dnabarcoder). The table also reports the number of representative sequences, sequences identified at genus and species levels, and the total number of genera and species detected by each approach.

DADA2 detected 2004 ITS2 sequences—slightly more than the 1954 OTU representatives from the original study. These sequences were classified using *dnabarcoder* against the yeastITS2 and filfungalITS2 datasets with taxon‐specific similarity cutoffs, and the results were merged. Alternatively, classification against CBSITS2, consisting of filfungalITS2 and yeastITS2, in a single run is possible but may identify fewer yeast species due to its stricter species‐level threshold for *Ascomycota*, reflecting the higher resolution needed for filamentous fungi.

**FIGURE 8:**
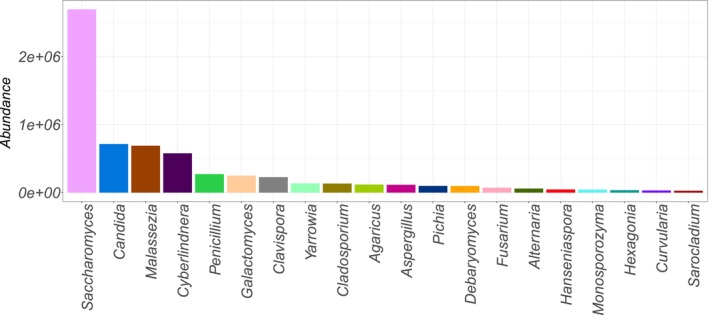
Most abundant genera detected in human gut microbiome samples using our approach. This figure highlights the yeast genera with the highest relative abundance identified from human gut microbiome samples, illustrating the composition and dominance patterns of the mycobiota revealed by our dnabarcoder‐based workflow.

Of the 2004 sequences, 974 were assigned to 19 fungal taxonomic classes. Although we detected fewer genera than in the previous study (192 vs. 234) due to the lack of reference sequences, we identified more sequences at the genus level (795 vs. 704). Among the identified genera, the 20 most abundant were *Saccharomyces*, *Candida*, *Malassezia*, *Cyberlindnera*, *Penicillium*, *Galactomyces*, *Clavispora*, *Cladosporium*, *Agaricus*, *Aspergillus*, *Pichia*, *Debaryomyces*, *Fusarium*, *Yarrowia*, *Alternaria*, *Hanseniaspora*, *Monosporozyma*, *Hexagonia*, *Curvularia* and *Sarocladium* (see Figure 8). These include all 14 genera (in bold) that were also identified as the most abundant in the previous study.

Notably, we detected 317 species or closely related species from 654 sequences that were not reported in the previous study, of which 35 species names were not present in the UNITE database (see Appendix S4 for details). These filamentous fungi and yeasts are associated with food (both fermented and spoilage), plants, soil, skin, air and indoor environments.

Some are known to colonise humans shortly after birth, such as *Candida* species (Stewart et al. 2013), while others can enter the body through food (e.g., *Agaricus bisporus, Alternaria alternata, Aspergillus candidus, Aspergillus carbonarius, Aspergillus intermedius, Aspergillus niger, Aspergillus penicillioides, Aspergillus restrictus, Aureobasidium melanogenum, Bipolaris sorokiniana, Bipolaris zeicola, Botrytis aclada*, *Debaryomyces fabryi, Bisifusarium domesticum (= Fusarium domesticum), Fusarium graminearum, Galactomyces candidus, Penicillium commune, Penicillium digitatum, Penicillium freii, Penicillium palitans, Penicillium polonicum, Pichia kluyveri, Rhizopus arrhizus, Rhizopus oryzae and Xeromyces bisporus*) or via indoor air (e.g., *Aspergillus ustus*, *Aspergillus versicolor*, *Aureobasidium pullulans*, *Cladosporium halotolerans*, *Parengyodontium album* and *Scopulariopsis asperula*) (Samson et al. 2019).

Within the human body, some fungi are true gut residents that can be cultured and are associated with serious diseases (e.g., *Candida* spp., van Thiel et al. 2022). In contrast, other species, such as *Malassezia*, may be present but are dead, dormant or simply difficult to culture.

**FIGURE 9:**
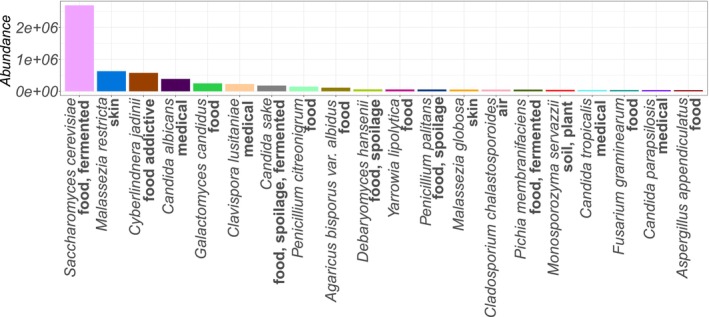
Top 20 most abundant filamentous fungal and yeast species in human gut microbiome samples, with associated living environments indicated in bold. This figure highlights the species with the highest relative abundances, providing insight into their ecological origins and potential environmental sources within the gut mycobiota.

Figure 9 shows the 20 most abundant fungal/yeast species and their associated living environments found in healthy human gut samples. Among them, *Saccharomyces cerevisiae, Malassezia restricta, Candida albicans, Candida sake, Cyberlindnera jadinii, Galactomyces candidus* (= *Geotrichum candidum*), *Malassezia globosa* and *Agaricus bisporus* were the eight species also included in the list of the 10 most abundant fungal/yeast species identified in the previous study (Nash et al. 2017).

The 20 most frequently detected species or closely related species in healthy human gut samples were: *Saccharomyces cerevisiae* , *Malassezia restricta*, *Malassezia globosa*, *Cladosporium chalastosporoides*, *Cyberlindnera jadinii*, *Alternaria alternata* , *Aspergillus sydowii*, *Fusarium graminearum*, *Aspergillus appendiculatus*, *Candida sake* , *Agaricus bisporus* var. *albidus*, *Cladosporium herbarum* , *Candida tropicalis* , *Fusarium napiforme*, *Debaryomyces hansenii* , *Debaryomyces fabryi*, *Cladosporium halotolerans*, *Penicillium palitans* and *Penicillium corylophilum*. In addition to fungal species commonly associated with food and food spoilage, there were also fungal species frequently found in air and indoor environments, such as *Cladosporium chalastosporoides* and *Cladosporium halotolerans*. This highlights the significant impact of both diet and the living environment on the composition of the gut mycobiota.

In summary, despite our reference barcode dataset being considerably smaller than UNITE, we successfully detected 317 fungal species, including all abundant genera and species previously identified using UNITE, underscoring the substantial influence of diet and living environment on human health.

## 4 Discussion

As yeast taxonomy advances, there is a growing recognition of the limitations of single‐locus DNA barcoding—particularly the ITS and LSU regions—which often lack sufficient resolution to discriminate between closely related or otherwise cryptic species. To overcome these limitations, researchers are increasingly shifting toward multigene and whole‐genome‐based phylogenies for more robust yeast species delineation and evolutionary inference (Kurtzman et al. 2008; Wang et al. 2015; Shen et al. 2018; Meyer et al. 2019). Multigene approaches, incorporating markers such as RPB2, TEF1 and others, provide higher phylogenetic resolution and help resolve complex relationships that single markers cannot (Kurtzman and Robnett 2013; Sakpuntoon et al. 2020; Heidler von Heilborn et al. 2023). Meanwhile, whole‐genome sequencing offers an unprecedented level of detail, enabling comprehensive comparisons of genome‐wide divergence, gene content and synteny (Groenewald et al. 2023; Liu, Hu, Yurkov, et al. 2024; Liu, Hu, Zhao, et al. 2024). This genomic perspective not only facilitates more accurate species identification and delimitation but also offers insights into functional and ecological traits.

Fungal metagenomics builds on the foundations of fungal genomics by enabling culture‐independent, comprehensive analysis of fungal communities across ecosystems (Sharpton 2014; Forbes et al. 2017). Unlike targeted amplicon methods, it captures entire genomes or large genome fragments from environmental samples, allowing simultaneous taxonomic and functional insights. This approach is especially valuable for complex or poorly understood communities—such as soil, plant microbiomes, indoor environments and the human microbiome—where fungi, including yeasts, play key but often underappreciated roles (Lindahl and Kuske 2013; Nash et al. 2017; Voidaleski et al. 2023).

Despite their great potential, fungal genomics and metagenomics still face several key challenges. Although the cost of whole‐genome sequencing has decreased substantially—making it a realistic alternative to multilocus Sanger sequencing—the main bottlenecks now lie in data processing and analysis rather than in sequencing itself. Generating and interpreting genomic data require advanced computational skills, yet user‐friendly, taxonomist‐oriented software remains scarce, as most existing tools demand considerable bioinformatics expertise. There is a pressing need for tools that can efficiently handle genome assemblies to produce multi‐gene phylogenies, calculate average nucleotide identity (ANI) values, and extract other biologically meaningful metrics in an accessible manner. Furthermore, the effectiveness of genomic and metagenomic approaches depends on the availability of high‐quality reference genomes, which remain limited for many yeast groups, restricting taxonomic resolution. In metagenomic studies, fungal DNA typically constitutes only a small fraction of environmental samples, making it challenging to detect low‐abundance species without deep sequencing. Even when sufficient sequencing data are available, the relatively large genome sizes and higher intron densities of certain yeast species, such as *Cryptococcus* spp., can complicate genome assembly and annotation (Janbon et al. 2014). The overall technical complexity of reconstructing eukaryotic genomes from mixed samples—combined with the scarcity of comprehensive reference data—continues to hinder accurate classification (Renzi et al. 2024).

Long‐read sequencing from Pacific Biosciences (PacBio) and Oxford Nanopore Technologies (ONT) offers promising advances for ITS‐based metabarcoding, particularly in fungal and yeast research (Reuter et al. 2015; Tedersoo and Anslan 2019; Hoang et al. 2021; Eshghi Sahraei et al. 2022; Graetz et al. 2025). The ability to sequence the full ITS region (ITS1, 5.8S and ITS2) in a single read enhances taxonomic resolution, especially for closely related species—an advantage over short‐read platforms that capture only partial regions. However, long read sequencing's relatively high error rate can compromise accuracy without proper error correction. Continued improvements in basecalling, polishing tools, and well‐curated full‐length ITS reference databases are essential to increase the reliability of long read sequencing based metabarcoding (Tedersoo et al. 2018).

Until these advanced methods are widely adopted, ITS and LSU remain essential first‐line markers for identifying filamentous fungi and yeasts, and for preliminary screening in large‐scale biodiversity studies due to their high amplification success, broad taxonomic coverage and extensive public database representation.

The curated CBS yeast barcode dataset presented in this study marks a significant advance in filling persistent gaps in the public reference databases that have long hindered accurate identification of yeasts in DNA barcoding and metabarcoding. By incorporating over 7000 ITS and nearly 8000 LSU barcodes comprising 2856 ITS and 3815 LSU new barcodes—with a significant proportion derived from type strains—this resource not only enhances taxonomic coverage but also reflects the most recent nomenclatural revisions, thereby aligning barcode data with current fungal taxonomy.

During the generation of the CBS yeast barcode dataset, we encountered several challenges commonly associated with ribosomal DNA sequencing, including variable amplification success, unreadable chromatograms, and intragenomic ITS heterogeneity. These issues were particularly evident in hybrid taxa such as *Saccharomyces* and *Cryptococcus* spp. Of the 3088 ITS and 4032 LSU sequences initially attempted, 2856 ITS (93%) and 3815 LSU (95%) sequences were successfully validated and retained after rigorous curation, with problematic or ambiguous sequences excluded. Our experience aligns with earlier reports (Xu 2016), which emphasise the need for optimised or taxon‐specific primers, the incorporation of supplementary markers (Stielow et al. 2015), and continued community‐based curation to overcome the technical and biological limitations of ITS‐based barcoding.

A considerable number of taxa in our study could not be reliably distinguished using ITS and/or LSU and were therefore excluded from the cutoff‐based analyses, including *Cryptococcus*, *Debaryomyces*, *Saccharomyces* and *Trichosporon*. In *Cryptococcus*, multilocus sequence data (e.g., Meyer et al. 2009; Hagen et al. 2015) are necessary to resolve species boundaries, while secondary biomarkers such as ACT1 for *Debaryomyces* (Groenewald et al. 2008) and IGS1 for *Trichosporon* (Sugita et al. 2002) have proved particularly informative. These markers frequently exhibit divergence patterns consistent with, or exceeding, those observed for ITS and LSU, supporting the original taxonomic assignments. In *Saccharomyces*, whole‐genome data provide a robust alternative for accurately distinguishing closely related species, including hybrids, clarifying relationships where traditional markers fail (Liti et al. 2009). Multilocus analyses or genome‐based phylogenies have shown that some taxa are truly distinct, while others are very similar and may need to be merged (Samson et al. 2014). Combining these approaches and developing reliable molecular cutoffs will be essential for accurately identifying species in these challenging groups.

One of the key outcomes of this study is the enhanced identification accuracy achieved using our updated and curated barcode dataset. Compared to the UNITE version we used, our dataset improves species‐level resolution, especially for recently reclassified taxa. We observed an increase in genus‐level resolution in the *Saccharomycetales* and *Serinales* following the reassignment of several *Candida* species into new genera (Liu, Hu, Yurkov, et al. 2024; Liu, Hu, Zhao, et al. 2024; Zhu et al. 2024). However, the low resolving power in certain taxonomic groups—especially within *Ascomycota* families such as *Debaryomycetaceae* and *Metschnikowiaceae*, and within poorly resolved orders such as *Serinales*—indicates that taxonomic clarity in these groups remains incomplete, underscoring the need for multigene and genome‐based phylogenies to resolve polyphyletic genera and ensure stable classifications.

Additionally, the significant taxonomic imbalance in both CBS and UNITE datasets reflects a bias toward well‐studied taxa, limiting accurate classification of diverse fungal communities and emphasising the need to generate barcodes for rare and understudied yeast species from environmental and clinical sources. Future improvements in classification accuracy may be driven by AI‐based solutions such as *MycoAI* (Romeijn et al. 2024), which offer a promising avenue for managing the complexity of large, imbalanced barcode datasets.

Although the CBS collection has made extensive efforts to include type strains for all described yeast species, a fraction remains unrepresented. Our yeast barcode dataset covers ca. 1700 species out of ca. 3500 described yeast names over time (Boekhout et al. 2022), including synonyms of the roughly 2000–2200 currently accepted species in TheYeasts.org. This highlights the broad coverage of the CBS collection while also illustrating the challenges of incomplete deposition, such as missing type strains and administrative or regulatory barriers, which continue to limit full representation of yeast diversity.

Our comparison of barcoding markers highlights the critical role of marker selection in yeast identification. ITS remains the most effective marker across most taxonomic levels. ITS1, despite exhibiting greater sequence variation, does not always outperform ITS2 in species discrimination. In fact, ITS2 performed well at the species and genus levels, whereas ITS1 was more effective at broader taxonomic ranks. These observations suggest that marker selection should be tailored to the taxonomic level and group of interest. Notably, longer nrDNA regions such as ITS and LSU consistently outperformed the shorter ITS1 and ITS2 regions, highlighting the utility of full‐length markers in both taxonomic and ecological studies.

In parallel, our results underscore the impracticality of universal similarity cutoffs and highlight the importance of establishing marker‐ and taxon‐specific thresholds for yeast classification, a pattern also observed in our previous study (Vu et al. 2022) on filamentous fungi.

Reclassifying the human gut microbiome dataset from Nash et al. (2017) using our updated barcodes demonstrates the value of taxonomic curation. Despite containing fewer than 20,000 sequences—far less than UNITE—our CBS dataset revealed 317 species, including all dominant fungal genera and species reported in the original study. While some identifications may reflect closely related taxa due to the limited resolution of ITS2, the results emphasise the significant impact of diet and environment on human health. This also suggests that large databases may not be essential for low‐diversity samples like the human microbiome; rather, well‐curated, up‐to‐date reference datasets with comprehensive ecological metadata are key.

The success of high‐throughput biodiversity and metabarcoding studies depends critically on well‐maintained and trustworthy reference systems. However, developing and sustaining user‐friendly platforms for curated reference data remains a significant challenge, as these efforts require traditional taxonomic expertise and long‐term institutional commitment rather than short‐term project funding. Continued and stable investment in such infrastructure is essential to ensure that the scientific community can access accurate, curated data for reliable species identification and meaningful comparative analyses.

Ultimately, this work provides a robust methodological framework and an expanded, expertly curated reference dataset for yeast and filamentous fungal (meta)barcoding, offering a valuable resource for researchers, taxonomists, ecologists and clinical scientists engaged in DNA‐based identification and biodiversity studies. Future efforts should focus on integrating genomic data, targeting underrepresented taxa, and developing advanced classification tools, while maintaining and enhancing reliable reference systems. Collectively, these steps will enable researchers across disciplines to achieve a more comprehensive and precise understanding of fungal diversity in natural and applied settings.

## Disclosure

Data Accessibility and Benefit‐Sharing: The new yeast barcodes have been submitted to GenBank under submission numbers SUB15285597 (ITS) and SUB15290020 (LSU), as part of project PRJNA351778. The corresponding WI and GenBank IDs are listed in Appendix S1. These barcodes are also accessible via our newly launched website (https://wi.knaw.nl/barcode), along with other validated DNA barcodes for filamentous fungi, and have been incorporated into UNITE and BOLD (Ratnasingham et al. 2024). Taxon‐specific similarity cutoffs for ITS, ITS1, ITS2 and LSU—determined in this study—are provided in Appendices S2, S3, [Link], [Link], [Link] and can be used for yeast sequence classification in DNA (meta)barcoding studies via BLAST or the *dnabarcoder* tool. All steps for calculating cutoffs and classifying the gut microbiome samples are detailed on our GitHub page http://github.com/vuthuyduong/yeastBarcoder to promote reproducibility.

## Conflicts of Interest

The authors declare no conflicts of interest.

## Supporting information


**Appendix S1:** men70082‐sup‐0001‐AppendixS1.xlsx.

**Appendix S2:** men70082‐sup‐0002‐AppendixS2.json.

**Appendix S3:** men70082‐sup‐0003‐AppendixS3.json.

**Appendix S4:** men70082‐sup‐0004‐AppendixS4.xlsx.

**Appendix S5:** men70082‐sup‐0005‐AppendixS5.xlsx.

**Appendix S6:** men70082‐sup‐0006‐AppendixS6.xlsx.

**Appendix S7:** men70082‐sup‐0007‐AppendixS7.pdf.

**Appendix S8:** men70082‐sup‐0008‐AppendixS8.pdf.

**Appendix S9:** men70082‐sup‐0009‐AppendixS9.html.

**Appendix S10:** men70082‐sup‐0010‐AppendixS10.json.

**Appendix S11:** men70082‐sup‐0011‐AppendixS11.json.

**Appendix S12:** men70082‐sup‐0012‐AppendixS12.json.

**Appendix S13:** men70082‐sup‐0013‐AppendixS13.html.

**Appendix S14:** men70082‐sup‐0014‐AppendixS14.json.

## Data Availability

The data that support the findings of this study are openly available in NCBI‐GenBank at https://www.ncbi.nlm.nih.gov/, reference number KIXT01000001.1–KIXT01002856.1, KIXS01000001.1–KIXS01003815.1.
