# Correction to “Advancing Yeast Identification Using High‐Throughput DNA Barcode Data From a Curated Culture Collection”

**DOI:** 10.1111/1755-0998.70106

**Published:** 2026-02-01

Vu, D., M. de Vries, B. G. van den Ende, et al. 2026. “Advancing Yeast Identification Using High‐Throughput DNA Barcode Data From a Curated Culture Collection.” *Molecular Ecology Resources* 26, no. 1: e70082. https://doi.org/10.1111/1755‐0998.70082.

In the published article, one author's name was incorrectly presented. The name ‘Magarita Hernández‐Restrepo’ should be corrected to ‘Margarita Hernández‐Restrepo’.

In addition, errors occurred in the numbering of items in Sections 2.2 (‘Methods’) and 3 (‘Results’), where citation references were mistakenly used instead of numerical list indicators.

Section 2.2 Methods

Incorrect text:

We used dnabarcoder (Vu et al. 2022, available at https://github.com/vuthuyduong/dnabarcoder) to: (Abarenkov et al. 2016) analyse the CBS yeast barcode datasets including their taxonomic classification as well as the intra‐specific variation within taxonomic groups (Abarenkov et al. 2022); predict global and local similarity cutoffs and resolving powers of the biomarkers ITS, ITS1, ITS2 and LSU for yeast identification; and (Abarenkov et al. 2024) reclassify the human microbiome dataset against the yeastITS2 and filfungalITS2 datasets using the similarity cutoffs predicted for yeast and filamentous fungal identification.

Correct text:

We used dnabarcoder (Vu et al. 2022, available at https://github.com/vuthuyduong/dnabarcoder) to: (1) analyse the CBS yeast barcode datasets including their taxonomic classification as well as the intra‐specific variation within taxonomic groups; (2) predict global and local similarity cutoffs and resolving powers of the biomarkers ITS, ITS1, ITS2 and LSU for yeast identification; and (3) reclassify the human microbiome dataset against the yeastITS2 and filfungalITS2 datasets using the similarity cutoffs predicted for yeast and filamentous fungal identification.

Section 3 Results

Incorrect text:

We evaluated CBS yeast barcodes (yeastITS and yeastLSU) and their resolving power for yeast identification by: (Abarenkov et al. 2016) analysing their taxonomic classification, sequence distribution, and intra‐specific variation within different yeast taxonomic groups (Abarenkov et al. 2022); calculating the number of indistinguishable yeast species using ITS and LSU (Abarenkov et al. 2024); predicting global and local similarity cutoffs and the resolving power of different biomarkers (viz. ITS, ITS1, ITS2 and LSU) based on the CBS yeast barcode datasets; and (Alper et al. 2011) examining whether recent name changes in 2024 (Liu, Hu, Yurkov, et al. 2024; Liu, Hu, Zhao, et al. 2024; Zhu et al. 2024) improved the resolving power of ITS and LSU for yeast identification. We highlighted the importance of well‐curated databases with up‐to‐date taxonomy by reclassifying the human microbiome dataset using the yeastITS2 and filfungalITS2 datasets with predicted similarity cutoffs, and by comparing the results with those from Nash et al. (2017).

Correct text:

We evaluated CBS yeast barcodes (yeastITS and yeastLSU) and their resolving power for yeast identification by: (1) analysing their taxonomic classification, sequence distribution, and intra‐specific variation within different yeast taxonomic groups; (2) calculating the number of indistinguishable yeast species using ITS and LSU; (3) predicting global and local similarity cutoffs and the resolving power of different biomarkers (viz. ITS, ITS1, ITS2 and LSU) based on the CBS yeast barcode datasets; and (4) examining whether recent name changes in 2024 (Liu, Hu, Yurkov, et al. 2024; Liu, Hu, Zhao, et al. 2024; Zhu et al. 2024) improved the resolving power of ITS and LSU for yeast identification. We highlighted the importance of well‐curated databases with up‐to‐date taxonomy by reclassifying the human microbiome dataset using the yeastITS2 and filfungalITS2 datasets with predicted similarity cutoffs, and by comparing the results with those from Nash et al. (2017).

We apologize for these errors.

