# Supplementary material for: Advancing Yeast Identification Using High‐Throughput DNA Barcode Data From a Curated Culture Collection
Source: Mol Ecol Resour. 2025 Nov 26;26(1):e70082. doi: 10.1111/1755-0998.70082 (PMC12649295; doi:10.1111/1755-0998.70082)
Supplement: Supplementary file 9 — Appendix S9: men70082‐sup‐0009‐AppendixS9.html. [file MEN-26-e70082-s002.html]

Javascript must be enabled to view this page.

magnitude
magnitudeUnassigned

yeastITS.classification.krona

7190

7190

2824

1741

235

92

16

1

8

2

1

4

76

1

48

10

2

15

143

81

16

5

8

1

4

1

5

2

4

3

1

27

1

3

49

1

6

1

1

1

18

6

2

7

4

2

10

1

1

4

1

2

1

3

1

2

1022

14

1

1

13

1

8

2

2

136

1

1

2

2

131

125

2

1

2

1

2

1

1

7

1

1

6

6

61

30

1

2

1

5

3

3

1

12

1

1

31

1

1

1

6

1

1

1

1

1

1

1

1

2

1

1

1

1

1

1

1

5

28

1

1

1

1

1

1

1

1

1

1

1

1

2

2

1

2

2

1

1

1

1

1

1

1

16

1

2

3

2

1

1

1

1

4

5

1

1

4

1

1

1

1

1

1

1

48

48

5

1

11

2

7

2

1

3

1

4

2

1

1

2

1

4

131

21

1

1

1

8

1

1

1

1

2

1

1

2

81

1

1

2

16

3

1

25

4

2

1

22

1

1

1

28

1

7

8

1

1

1

2

2

2

1

1

1

1

1

11

11

1

2

3

1

1

3

4

2

2

2

2

33

7

1

1

3

2

26

1

3

1

1

1

2

1

1

2

1

1

1

1

1

1

2

1

1

1

2

503

499

1

2

15

1

1

66

2

46

104

1

3

5

1

3

7

1

1

17

211

1

1

2

1

5

1

4

1

1

1

1

2

2

22

14

1

1

1

11

2

1

1

5

1

1

1

2

1

1

310

306

78

1

13

14

8

1

1

1

17

2

4

1

1

2

6

6

104

1

1

16

1

3

1

7

3

1

13

1

15

7

26

1

1

2

3

1

78

3

1

1

3

1

1

8

2

29

1

2

1

3

5

1

10

1

5

1

1

7

7

38

1

1

33

1

1

1

4

1

1

2

22

15

8

3

2

2

7

7

6

1

152

20

7

11

1

1

95

1

1

66

26

1

4

16

8

1

9

1

1

1

20

2

3

1

9

1

3

1

7

7

37

3

1

2

34

34

102

5

5

5

1

1

1

2

9

1

1

8

5

2

2

1

3

1

1

1

20

20

20

14

2

1

1

2

68

67

10

1

2

2

5

57

1

4

2

1

7

1

1

5

1

1

1

5

13

2

1

11

1

1

3

3

3

3

2

1

32

1

1

1

1

31

19

2

2

4

1

1

1

1

1

1

3

1

1

1

8

1

1

1

3

1

1

1

1

5

4

1

7

7

1

4

2

9

9

9

1

1

8

1

2

1

1

1

1

1

704

26

8

2

1

1

1

1

4

1

1

2

1

1

18

17

4

8

1

4

1

1

13

13

4

9

2

1

1

1

1

1

101

19

5

5

14

5

4

2

3

82

77

1

1

1

1

1

14

14

3

1

3

1

1

1

1

1

2

543

543

38

1

1

5

6

3

1

1

2

2

1

2

12

1

45

19

26

50

1

1

1

1

1

4

2

1

3

1

2

1

8

2

3

1

6

1

1

9

400

1

58

5

3

5

8

49

200

12

13

1

2

7

19

1

7

8

1

10

1

6

2

1

2

2

2

1

1

3

3

3

1

1

1

155

155

155

155

1

2

3

2

2

10

2

2

3

69

33

2

10

11

3

65

65

5

5

1

1

1

1

1

2

2

2

17

2

1

1

6

4

2

3

1

1

1

6

2

1

1

2

24

4

1

2

1

20

2

1

1

1

1

1

3

1

1

1

2

1

1

1

1

1

17

13

5

1

1

6

4

2

2

13

10

3

1

1

1

7

1

1

6

1

1

2

2

3

3

3

2

1

4366

3

3

3

3

1

2

13

13

13

13

2

3

8

1

1

1

1

1

7

7

7

7

1

1

5

12

12

12

3

3

2

1

1

7

3

1

1

2

1484

267

91

91

1

1

1

1

1

1

1

1

8

3

1

1

44

1

1

3

1

2

3

6

1

1

1

4

1

1

176

29

1

4

1

3

15

1

1

3

135

2

4

2

1

3

16

3

6

2

1

1

2

1

9

4

2

3

2

2

4

5

3

1

16

5

9

3

17

6

12

3

7

2

91

91

6

6

83

26

3

1

11

1

2

1

6

8

3

6

5

3

5

1

1

2

1

1

67

67

67

14

1

1

1

6

1

2

1

19

8

1

2

1

2

3

2

1

1

1059

1

1

1

1

1

1

1

1

1049

93

6

28

4

1

2

9

1

22

1

18

1

110

1

1

104

2

2

1

1

15

2

1

4

4

3

1

26

1

1

1

2

1

3

5

1

1

10

28

1

5

2

3

4

2

5

6

61

1

2

4

2

39

3

10

2

2

11

9

2

10

2

8

17

13

2

1

1

4

4

2

2

11

3

3

3

2

10

1

1

1

3

2

1

1

10

1

1

1

2

1

1

3

18

1

1

1

1

1

10

3

72

42

1

2

1

11

2

8

5

1

1

360

2

4

1

2

1

6

17

4

4

116

4

28

17

10

6

137

1

2

2

10

2

7

1

165

17

6

2

29

74

5

25

4

3

9

5

1

1

1

1

1

1

2

2

5

1

1

4

1

3

76

76

76

1

1

1

1

13

2

2

1

1

2

2

1

2

56

1

20

2

1

2

1

1

2

6

4

1

1

2

1

1

1

1

2

2

1

1

2

5

3

1

1

429

429

63

4

2

2

4

1

2

2

33

1

3

1

1

7

19

11

4

2

2

82

44

6

7

1

27

3

19

17

1

1

2

1

1

12

3

1

1

2

1

1

1

1

1

5

2

1

1

1

265

1

1

18

6

8

1

1

2

38

1

2

1

1

1

1

2

3

1

1

1

1

1

6

1

1

1

5

1

1

1

2

1

1

8

1

2

4

1

32

5

1

2

1

1

1

2

1

1

2

5

1

1

2

6

17

6

5

3

1

2

7

2

3

2

84

2

1

1

2

14

4

1

3

1

1

1

1

2

1

1

4

1

1

1

1

1

1

8

1

1

1

1

1

1

1

2

10

1

1

1

8

60

3

1

1

1

2

5

2

17

1

1

2

1

1

1

4

1

1

1

1

10

2

1

2341

42

42

8

5

3

2

2

32

11

1

1

1

4

1

2

1

1

2

5

2

464

464

2

1

1

84

1

1

3

1

1

2

1

1

1

2

1

2

2

2

1

2

2

1

2

1

2

1

1

5

1

1

2

8

2

1

1

6

1

1

1

1

1

3

6

9

26

3

1

2

1

1

3

1

3

1

1

4

1

1

1

1

1

19

1

1

5

12

27

1

1

10

1

2

12

15

2

2

11

232

2

4

6

1

1

1

1

15

10

44

1

1

3

2

1

5

4

5

2

40

3

1

10

4

3

1

1

18

1

1

31

1

1

1

6

26

3

1

1

1

4

1

2

7

1

1

1

1

1

1

6

1

5

27

8

19

1835

234

2

2

1

1

22

14

1

4

1

2

2

2

29

1

1

1

1

4

1

1

2

1

1

1

11

1

1

1

2

1

1

7

5

2

93

2

1

2

2

1

2

5

1

1

1

2

1

6

1

1

1

2

1

2

1

1

3

2

2

2

1

3

3

1

2

4

1

1

2

1

2

2

1

1

1

1

3

1

1

2

3

3

2

1

2

2

52

1

4

20

1

1

6

12

4

1

2

11

1

5

1

4

3

1

2

5

1

2

2

5

1

1

1

1

1

1601

210

4

39

1

8

2

1

1

2

1

1

2

133

7

3

3

1

1

38

2

1

2

1

1

1

1

1

1

1

1

1

1

14

5

2

1

1

53

5

9

6

1

1

7

2

7

10

3

2

767

1

1

1

2

1

2

1

1

1

3

5

1

9

14

2

1

2

1

1

1

1

2

3

1

1

4

1

1

1

3

1

1

2

1

68

3

1

1

1

1

45

2

4

1

27

2

2

2

12

3

2

1

16

3

1

1

1

1

2

2

1

2

1

1

1

1

4

1

2

2

10

2

1

1

3

6

10

1

4

1

116

1

6

8

2

2

2

6

1

20

1

1

1

2

1

65

1

1

1

1

2

1

1

1

1

1

1

1

2

2

1

1

1

1

2

5

1

2

2

1

1

3

4

5

1

1

1

2

1

1

1

5

1

1

2

1

2

1

1

1

5

1

7

3

7

8

1

1

2

1

11

1

1

1

1

6

1

5

2

1

2

1

1

2

4

11

3

5

4

1

1

3

2

1

1

3

7

1

2

2

5

6

1

2

3

32

1

7

2

1

2

4

1

14

27

24

3

2

2

6

5

1

3

3

76

5

1

1

1

1

4

10

1

1

24

25

1

1

15

1

2

2

1

2

7

37

1

5

1

5

1

9

1

1

1

1

1

4

1

1

1

2

1

13

1

12

3

2

1

93

1

2

14

2

8

1

2

1

1

3

3

1

1

2

1

13

16

8

1

1

11

36

1

1

14

2

1

3

1

5

1

4

2

1

6

1

5

139

16

7

1

35

76

1

2

1

2

2

37

1

1

2

2

4

6

3

18
