# Supplementary material for: Advancing Yeast Identification Using High‐Throughput DNA Barcode Data From a Curated Culture Collection
Source: Mol Ecol Resour. 2025 Nov 26;26(1):e70082. doi: 10.1111/1755-0998.70082 (PMC12649295; doi:10.1111/1755-0998.70082)
Supplement: Supplementary file 13 — Appendix S13: men70082‐sup‐0013‐AppendixS13.html. [file MEN-26-e70082-s004.html]

Javascript must be enabled to view this page.

magnitude
magnitudeUnassigned

yeastLSU.classification.krona

7912

7912

5187

4

4

4

4

3

1

1

1

1

1

1

88

88

88

71

30

1

1

1

1

2

1

1

1

1

2

2

5

1

1

1

2

2

1

1

1

6

1

1

4

6

1

1

4

9

1

1

1

1

1

1

2

1

1

1

1

1

32

32

32

15

6

9

4

4

11

1

1

4

5

2

1

1

1

1

1

1

1

1829

1280

1

1

1

1

1

2

2

1269

3

3

12

1

3

1

2

3

1

1

22

6

5

4

7

22

1

1

10

2

5

1

2

96

28

22

1

2

1

3

5

1

9

1

23

27

3

5

1

2

1

1

2

1

9

2

123

2

1

1

2

116

1

9

1

8

74

43

11

1

6

2

1

1

9

17

2

4

1

6

1

2

1

19

14

1

1

3

10

1

1

6

1

1

2

2

11

1

1

3

1

3

1

1

28

3

4

2

2

6

6

4

1

109

3

4

23

8

5

63

1

1

1

5

1

4

9

7

1

1

10

8

2

180

2

4

7

85

27

18

3

28

6

2

2

3

3

476

4

10

4

28

4

3

1

4

169

3

19

1

2

198

2

5

19

6

2

1

1

4

3

1

1

1

1

131

131

120

2

8

13

8

1

2

10

36

1

3

10

6

4

10

2

4

9

9

2

1

1

356

129

129

1

1

8

1

4

3

1

1

1

5

1

5

2

1

1

3

3

2

1

8

1

1

5

1

2

6

1

59

227

49

3

4

1

1

24

2

7

3

4

24

2

9

5

2

3

3

6

1

3

1

1

148

4

6

1

1

1

17

1

5

3

8

13

2

3

1

4

6

2

19

3

1

5

3

10

3

10

1

11

2

1

1

62

62

62

5

1

2

3

6

1

1

2

1

1

2

2

2

8

4

21

491

491

288

20

1

6

9

2

1

1

73

1

2

1

1

11

1

3

15

1

1

1

1

3

1

1

4

1

1

2

1

1

1

2

3

1

1

6

2

1

2

21

2

6

7

3

3

8

1

4

1

2

82

1

1

1

11

13

2

2

1

1

1

1

5

2

1

1

2

1

1

1

1

2

1

1

1

1

1

3

8

1

1

1

1

1

3

1

1

2

2

41

1

2

3

1

3

1

1

1

3

1

1

1

2

1

1

1

6

1

2

1

7

39

2

1

3

2

1

1

1

3

2

5

1

1

1

8

1

1

5

4

2

2

20

3

3

11

3

103

12

1

1

1

4

2

1

2

2

1

1

11

2

1

4

1

2

1

24

1

1

1

19

1

1

54

1

5

1

31

7

9

80

1

3

2

2

2

2

4

19

34

2

5

4

2677

49

49

9

6

3

3

3

37

5

4

10

2

1

2

1

1

1

3

2

3

2

2056

2

2

1747

3

2

1

41

2

2

2

4

18

7

1

5

794

13

1

47

1

1

3

4

1

1

3

2

2

5

70

2

2

2

7

2

2

3

1

5

3

16

1

3

1

2

1

2

1

3

1

1

3

1

1

1

2

3

4

1

1

1

1

5

1

2

2

5

2

1

1

1

10

1

3

2

1

1

96

5

1

1

1

2

2

2

1

5

1

1

3

2

1

3

3

1

1

1

1

3

2

1

12

3

30

2

1

1

1

1

2

1

1

8

2

2

4

1

2

8

1

2

2

10

1

1

2

21

9

1

1

3

2

2

2

2

1

3

8

1

1

2

3

7

1

2

1

1

1

2

1

1

1

1

1

2

5

1

4

1

3

12

1

2

2

1

4

1

1

65

1

2

1

1

4

1

8

1

1

3

1

6

2

1

11

2

1

1

3

1

4

2

1

6

1

6

9

2

2

1

1

1

3

2

1

99

1

2

1

1

9

2

9

5

1

2

3

14

10

1

2

2

1

8

2

3

1

1

1

17

63

1

1

1

1

4

1

2

1

7

1

1

21

2

3

1

6

1

1

1

1

2

2

1

25

2

2

3

2

2

10

4

44

1

1

10

1

1

1

1

1

9

1

1

4

9

1

2

6

1

5

248

1

2

2

4

37

2

2

1

8

10

2

11

1

2

4

1

158

2

2

2

2

5

5

2

1

1

75

9

4

15

1

8

8

18

5

2

1

4

66

1

20

2

2

1

4

1

4

1

24

5

1

8

4

1

3

37

13

10

1

1

8

2

2

41

2

1

1

2

13

6

3

1

1

1

2

1

1

1

1

1

1

1

1

33

29

4

2

2

139

16

2

6

33

80

1

1

12

11

1

307

49

1

1

6

2

5

1

3

13

1

15

1

1

1

3

1

2

7

2

1

4

36

1

3

2

1

1

1

1

2

17

1

3

1

2

6

2

4

13

1

6

5

1

2

2

2

2

147

1

2

1

2

2

1

1

3

1

2

2

1

5

1

2

2

2

1

2

1

1

1

2

15

1

2

1

7

6

1

1

3

3

2

13

1

2

3

3

2

2

1

2

4

2

1

1

1

1

12

1

2

3

3

1

2

1

2

2

1

1

1

1

29

16

1

1

7

1

1

2

1

1

8

1

1

1

1

1

1

1

1

572

572

18

11

5

2

25

6

3

4

1

1

2

2

1

1

2

1

1

26

1

1

4

1

3

2

1

3

4

1

1

3

1

103

1

2

1

1

3

9

3

1

1

2

2

2

2

2

3

1

2

2

1

1

1

2

1

5

3

1

1

1

2

2

2

9

2

2

1

6

1

3

2

14

4

2

2

49

30

19

1

1

272

2

4

8

5

1

5

11

2

9

16

46

3

4

35

5

1

1

1

1

18

1

1

1

21

6

2

1

1

1

45

1

1

11

1

13

2

1

10

14

1

1

2

1

9

23

1

1

14

1

2

4

24

1

7

12

1

1

1

1

55

55

55

55

6

40

9

9

9

9

9

2

2

5

2725

91

60

58

50

1

15

2

4

1

4

3

1

5

1

1

9

2

1

8

2

1

2

3

1

1

1

1

11

9

5

1

1

1

2

4

2

1

1

2

2

5

5

5

1

2

1

1

15

15

15

11

2

1

1

3

3

3

3

1

1

1

42

42

11

9

1

5

1

2

2

1

1

2

1

1

1

1

2

2

1

1

9

1

1

2

1

1

2

1

1

4

1

2

1

18

17

1

1

1

2

1

2

1

2

1

1

1

1

1

1

1

1

1684

970

2

2

13

12

2

2

7

1

1

1

20

5

1

2

1

1

1

1

1

2

3

1

1

39

39

1

7

1

2

5

4

1

1

4

2

1

1

1

2

6

59

30

1

1

1

6

4

1

2

1

1

2

1

1

1

1

1

1

1

1

1

1

29

1

2

12

1

4

1

1

4

3

5

4

1

1

1

1

1

1

33

26

1

2

1

1

1

1

2

1

2

1

1

1

1

1

1

3

1

2

1

1

7

3

1

1

2

5

4

4

1

1

123

2

2

117

1

1

1

112

2

2

2

2

1

1

14

14

1

3

1

2

1

3

3

3

2

2

1

1

119

71

1

9

5

1

22

2

22

1

2

1

1

1

1

2

1

1

21

1

2

1

9

1

1

1

2

1

2

26

1

7

1

1

1

2

7

1

1

1

2

1

1

1

1

22

2

1

1

1

1

14

1

11

1

1

5

2

1

2

480

475

5

2

3

3

1

2

33

3

1

55

1

1

14

2

2

15

2

2

98

1

1

223

1

3

1

5

1

1

1

1

1

32

1

1

1

1

1

3

2

1

2

1

1

1

1

1

1

1

1

2

1

1

1

1

2

1

1

1

213

92

72

42

2

1

1

8

18

20

4

2

10

1

3

121

1

1

70

5

1

3

1

4

3

4

3

10

16

1

1

18

39

6

1

2

1

1

1

12

4

5

1

5

11

1

1

1

6

2

316

312

81

1

2

1

9

2

2

1

9

1

5

30

1

1

6

2

2

1

5

39

1

1

33

2

1

1

7

7

1

1

75

13

1

1

1

9

7

2

2

13

1

3

5

15

2

109

1

3

2

1

3

1

1

1

12

5

1

9

1

13

15

29

7

2

2

4

1

2

1

164

99

33

4

2

1

1

17

1

2

2

2

1

9

8

1

55

5

1

15

7

1

22

3

1

1

1

1

1

45

41

41

4

3

1

20

1

1

11

7

21

14

8

1

3

2

7

7

6

1

12

3

3

3

2

1

9

6

5

1

1

2

1

1

1

3

1

1

1

35

1

1

1

1

34

5

4

1

8

8

2

2

4

21

5

1

1

1

2

9

4

1

1

1

1

1

1

1

2

1

1

2

2

2

1

1

6

6

6

6

1

1

1

1

1

1

167

167

167

167

15

3

3

2

9

3

1

2

2

3

10

39

69

4

2

685

15

15

4

11

3

3

3

1

1

1

94

73

69

2

1

1

21

15

3

6

2

4

6

6

2

2

1

1

1

1

16

16

1

1

3

1

1

1

1

1

4

2

26

18

18

4

9

4

1

8

1

1

2

1

1

4

1

2

1

1

1

527

527

35

1

1

1

5

1

1

11

3

2

1

2

1

5

58

1

3

2

1

2

2

3

1

2

5

1

1

10

11

6

1

1

1

2

2

49

23

26

8

1

5

2

377

187

6

3

1

2

1

8

11

5

6

4

20

2

10

7

56

47

1

2

2

2

1

1
