# Supplementary material for: Advancing Yeast Identification Using High‐Throughput DNA Barcode Data From a Curated Culture Collection
Source: Mol Ecol Resour. 2025 Nov 26;26(1):e70082. doi: 10.1111/1755-0998.70082 (PMC12649295; doi:10.1111/1755-0998.70082)

Agaricostilbales ITS

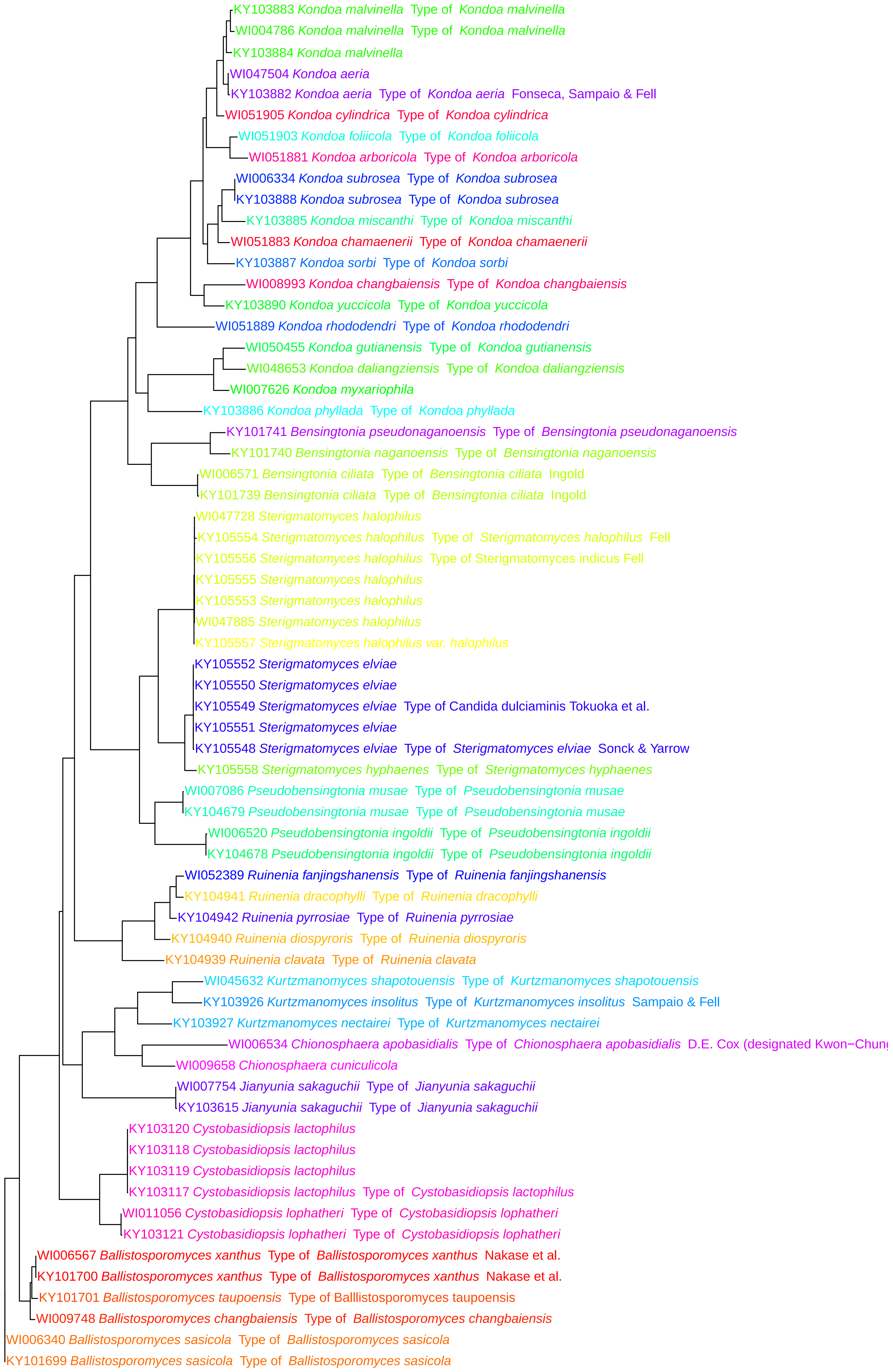

Alaninales ITS

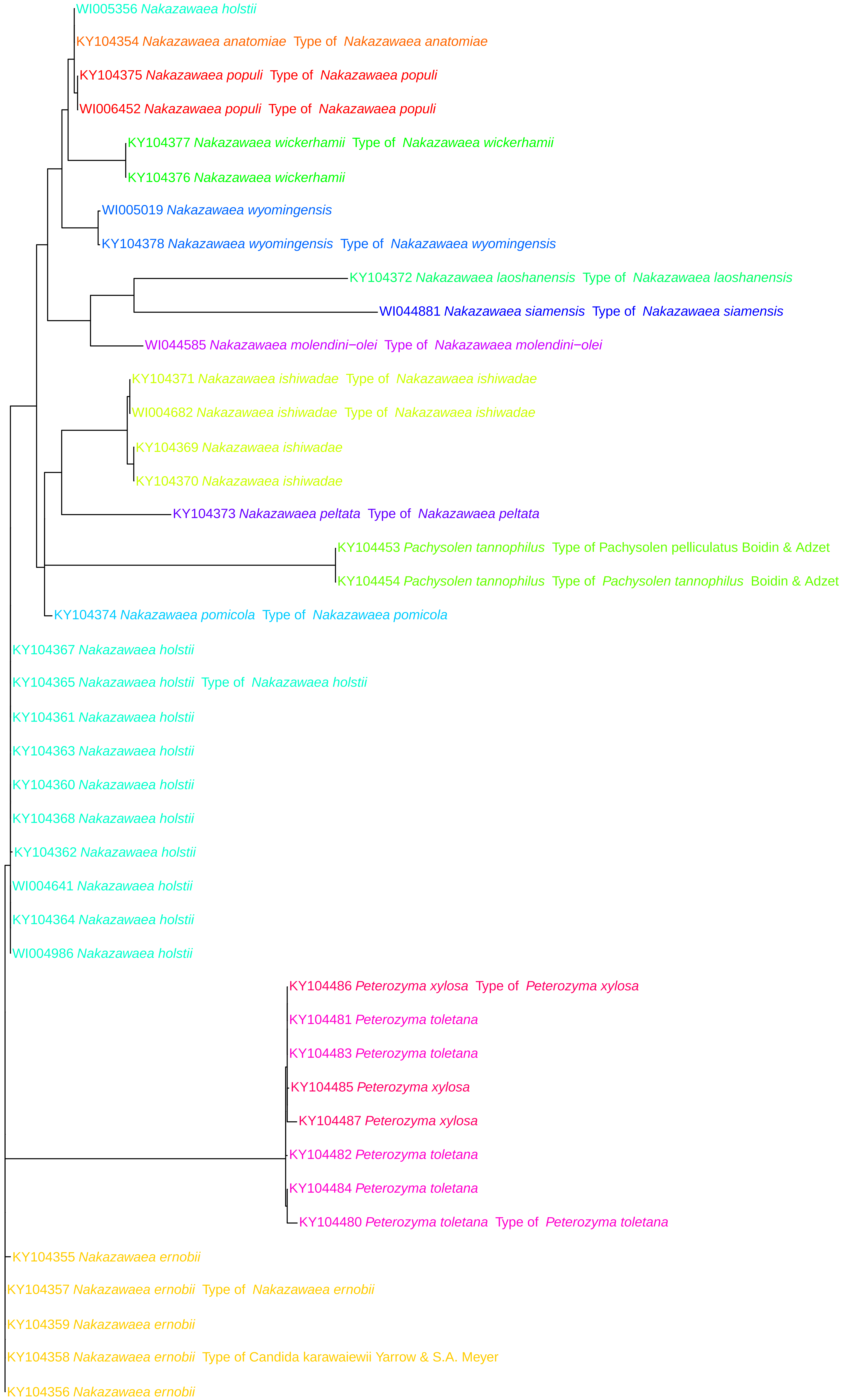

Ascoideales ITS

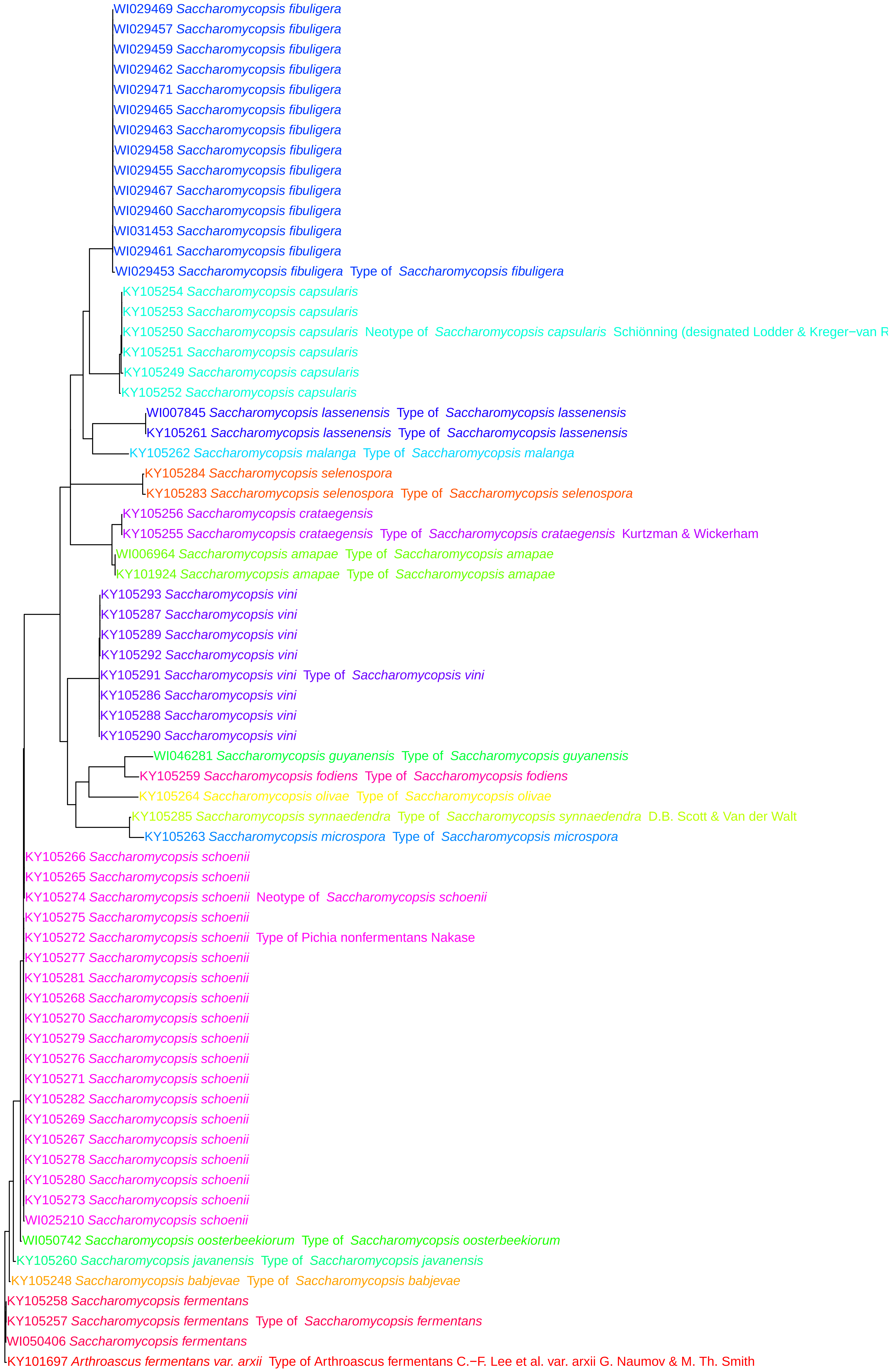

Buckleyzymales ITS

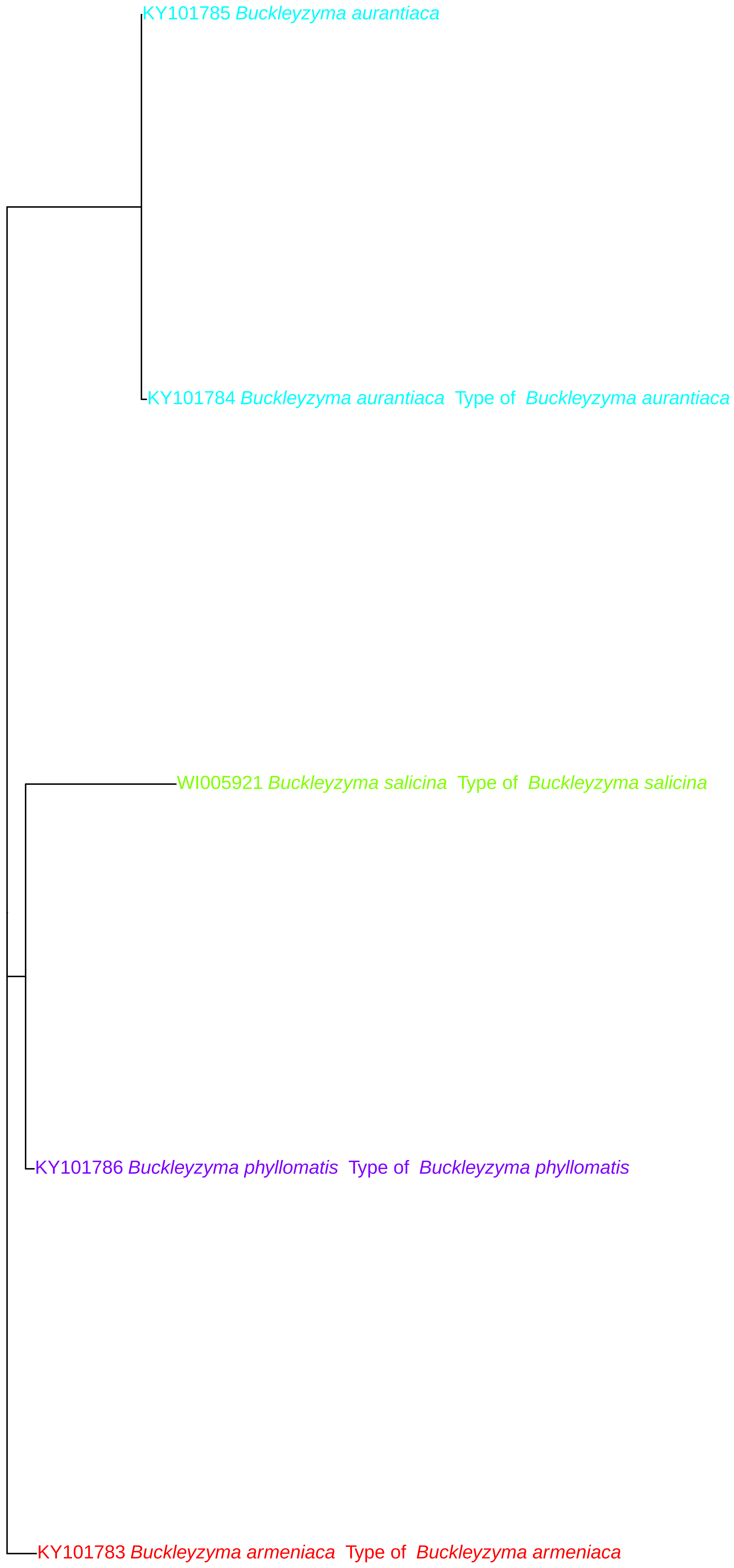

Cystobasidiales ITS

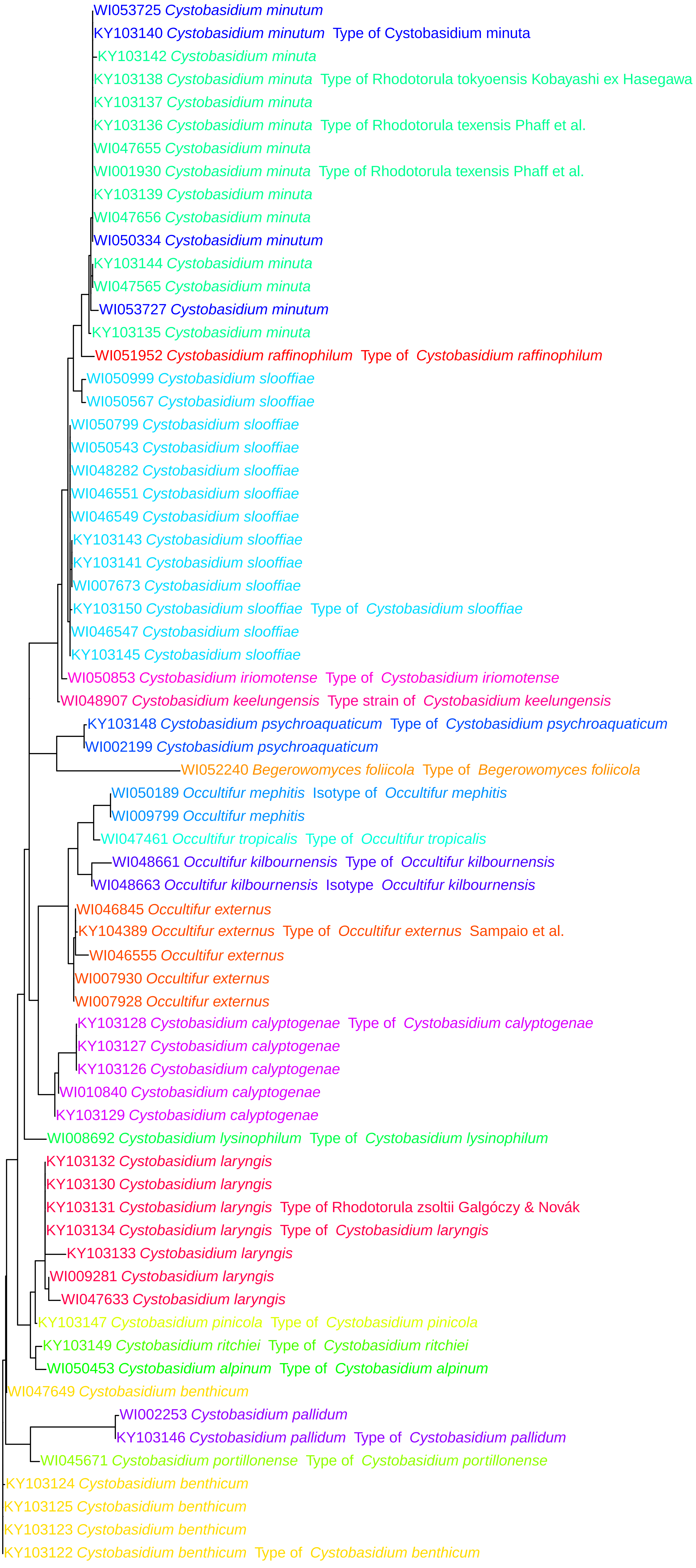

Cystofilobasidiales ITS

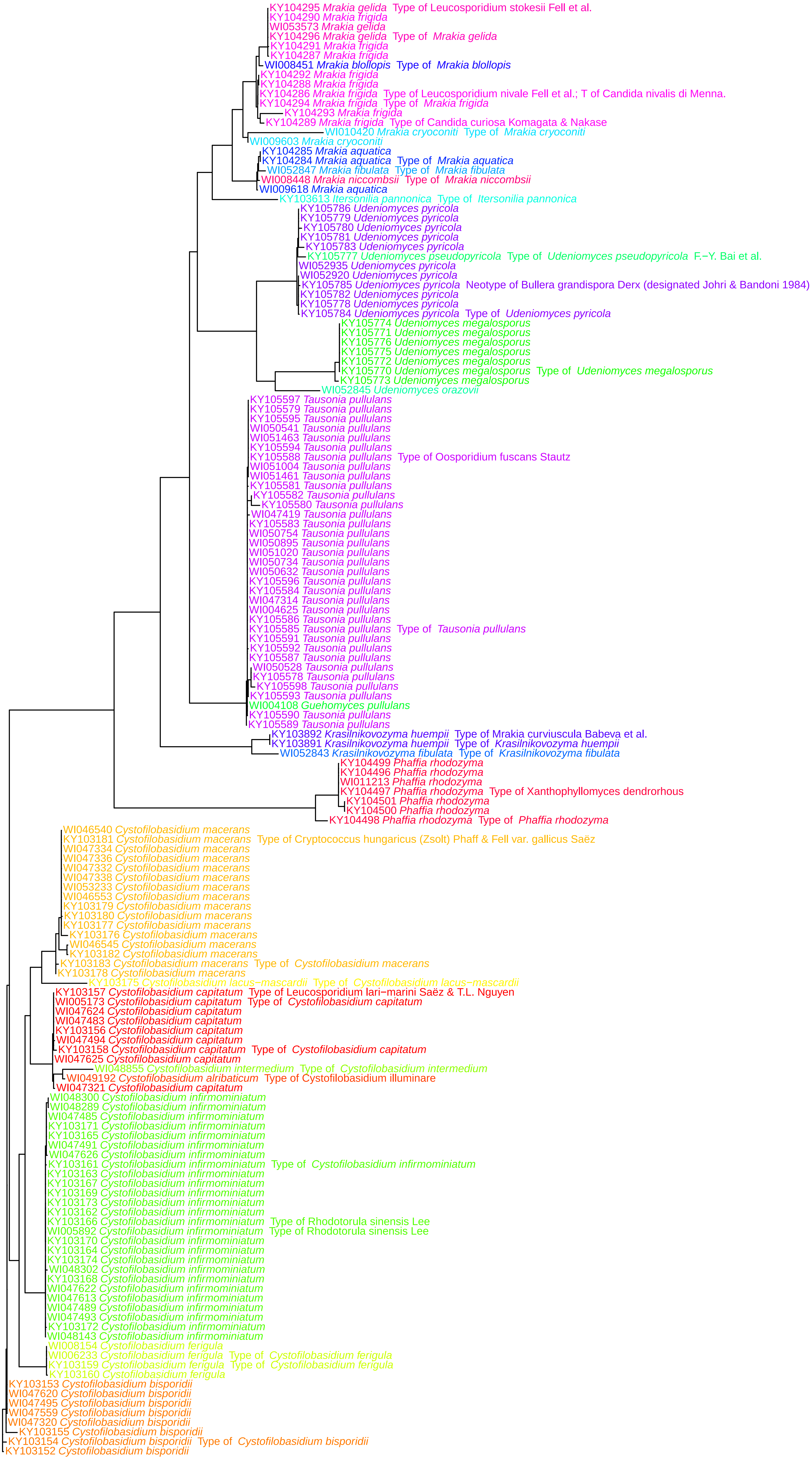

Dipodascales ITS

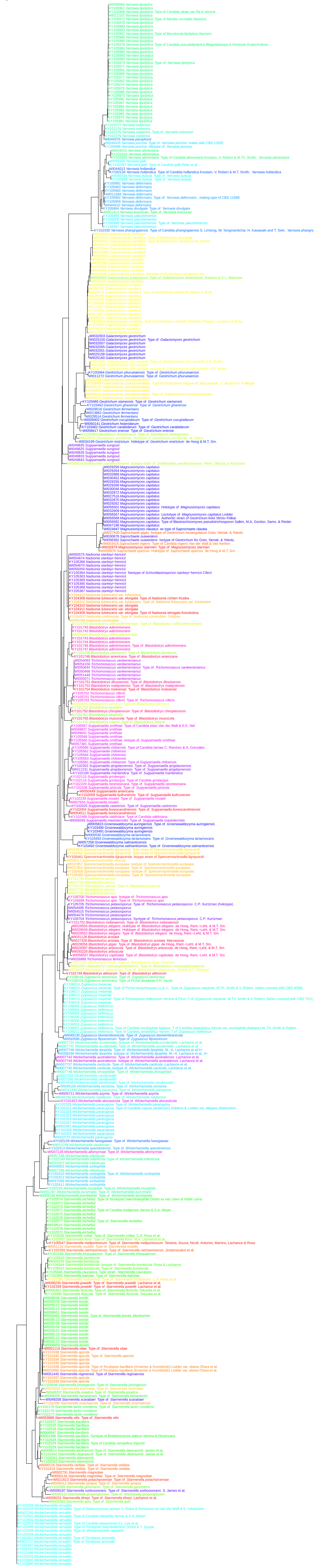

Filobasidiales ITS

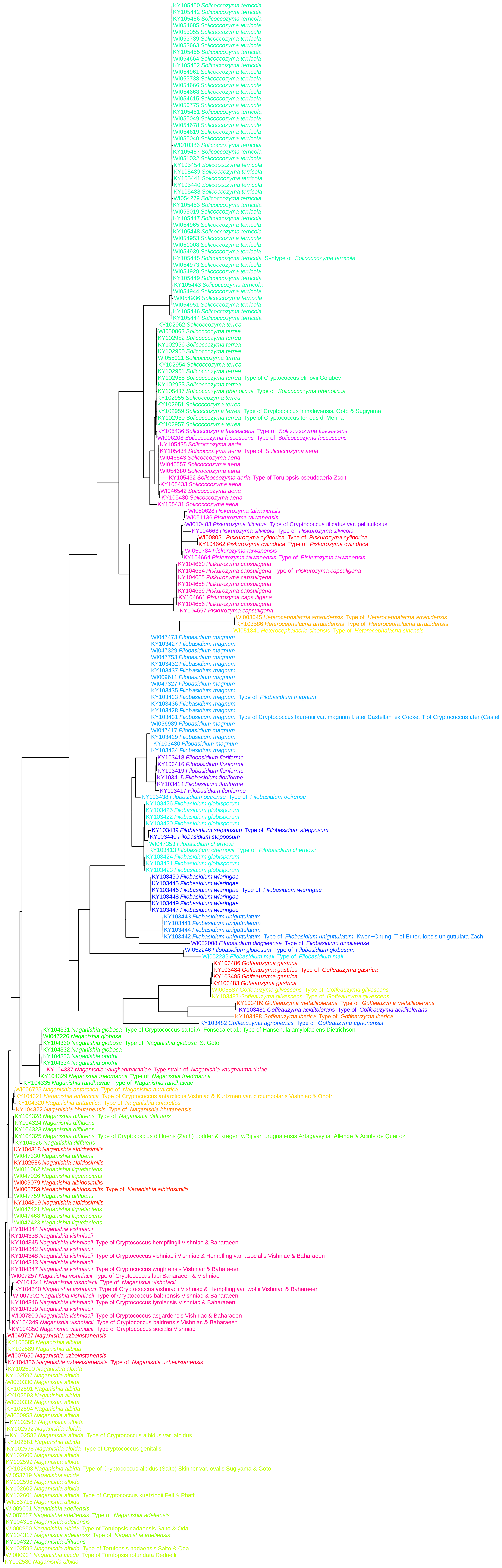

Heitmaniales ITS

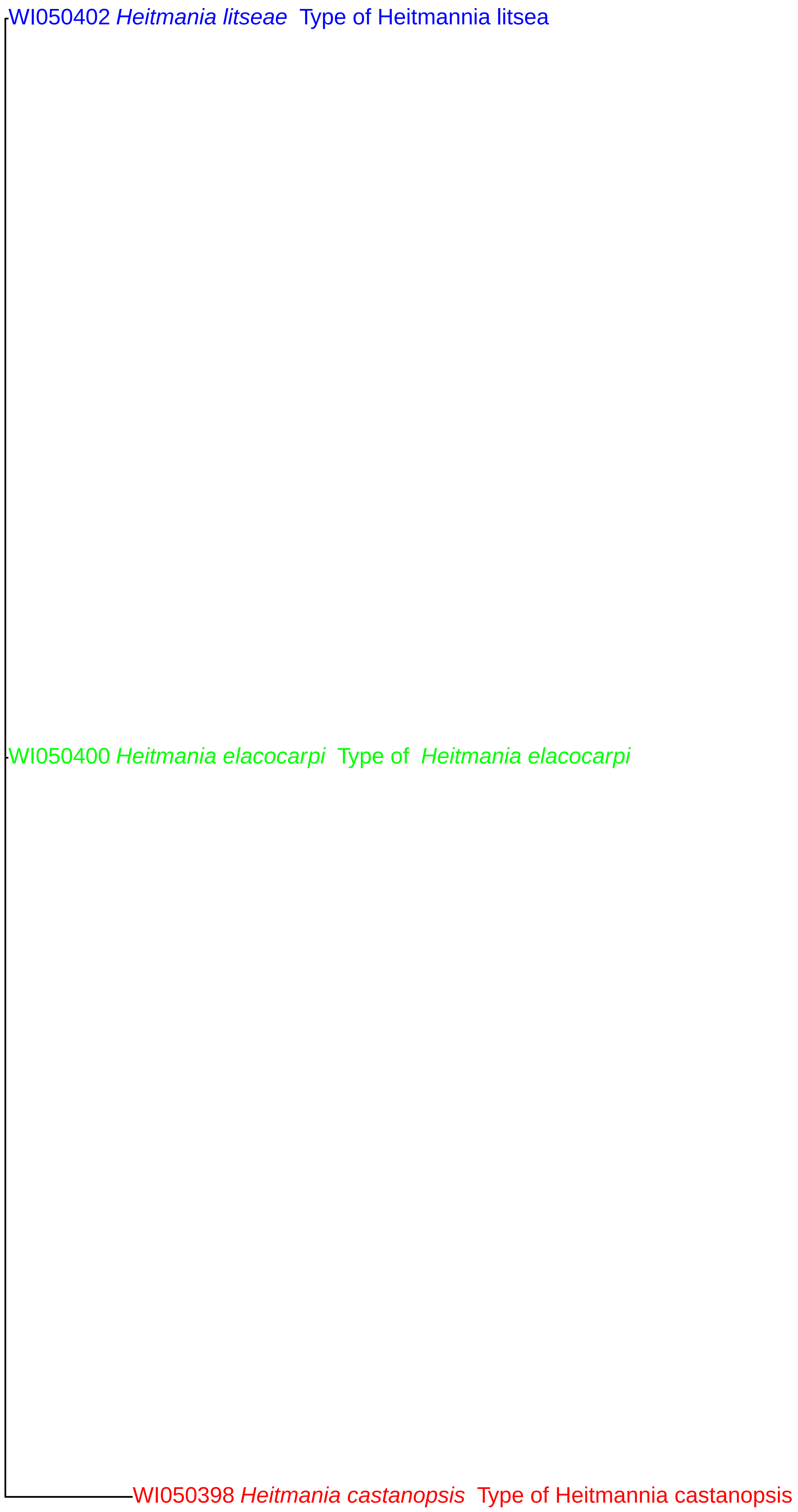

Heterogastridiales ITS

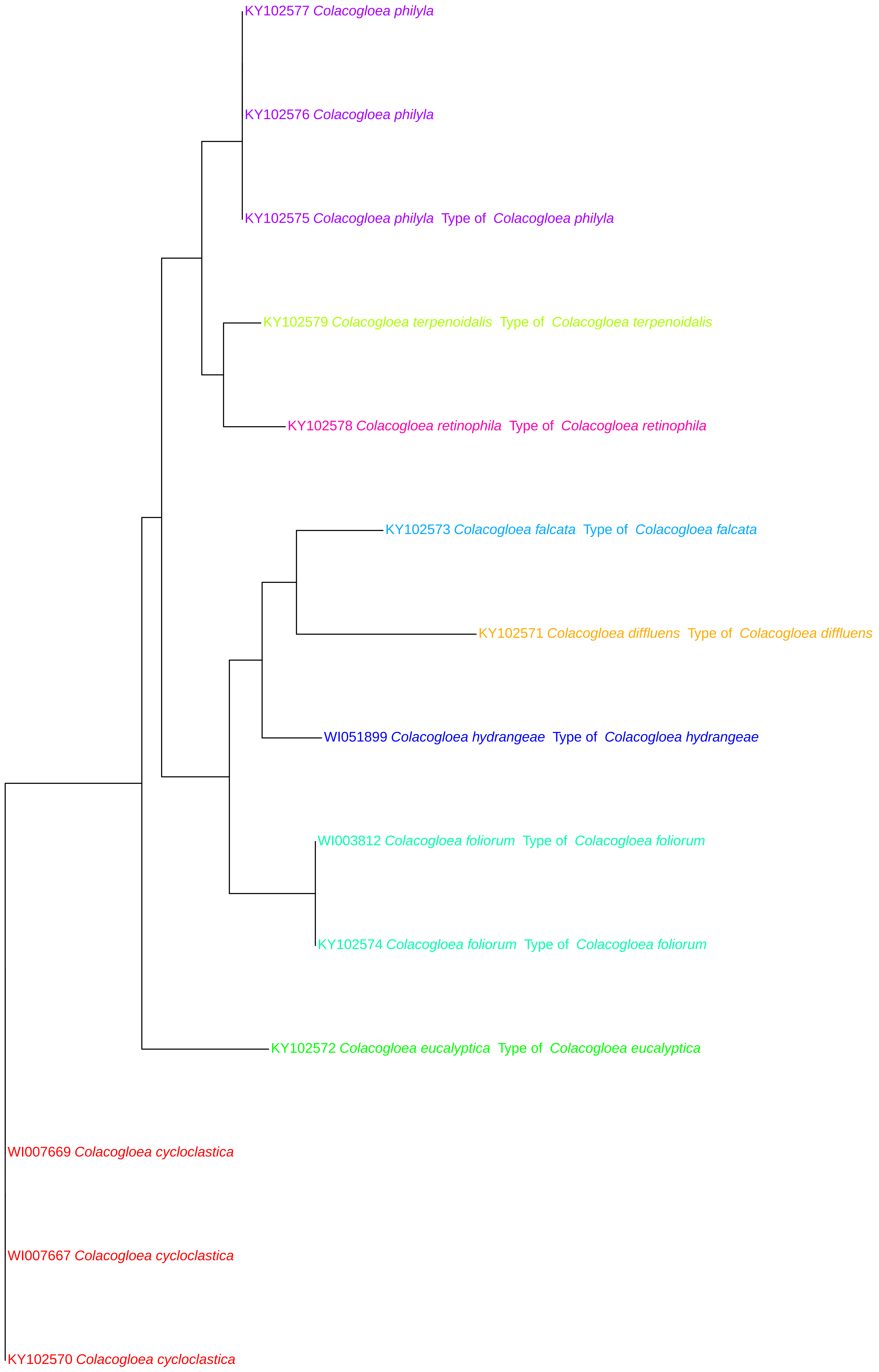

Holtermanniales ITS

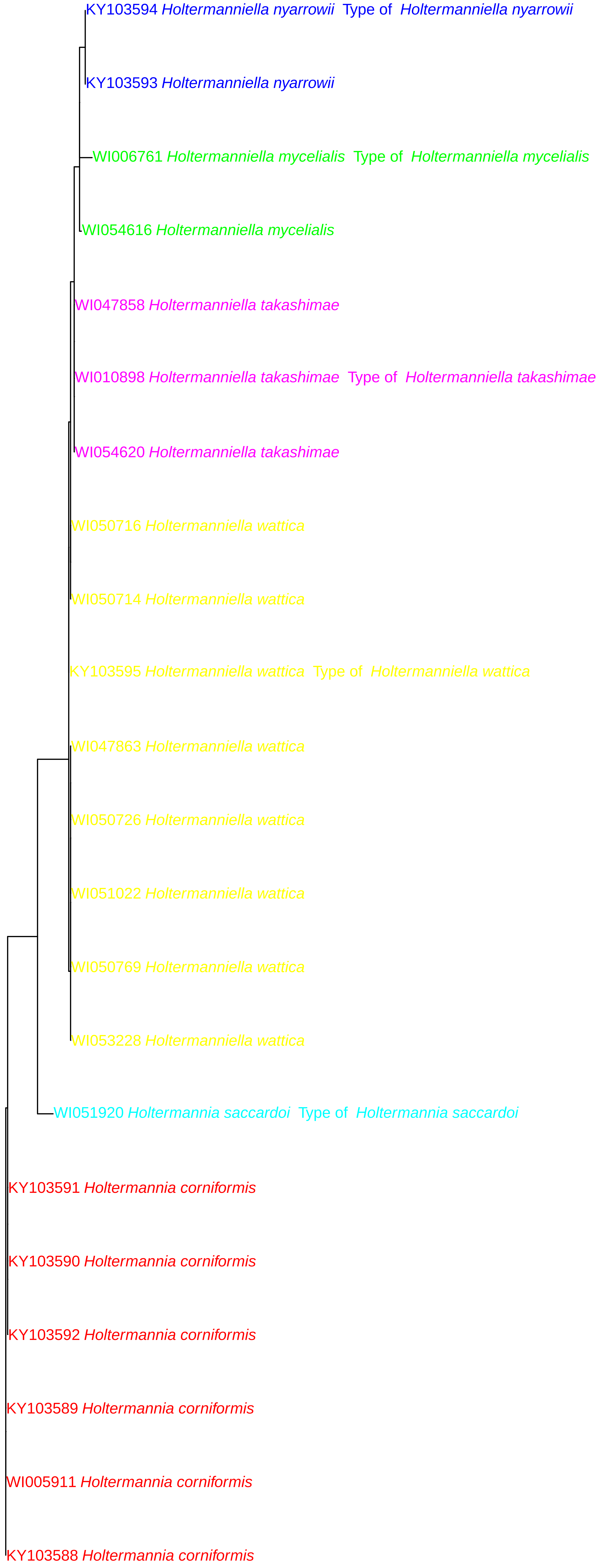

Kriegeriales ITS

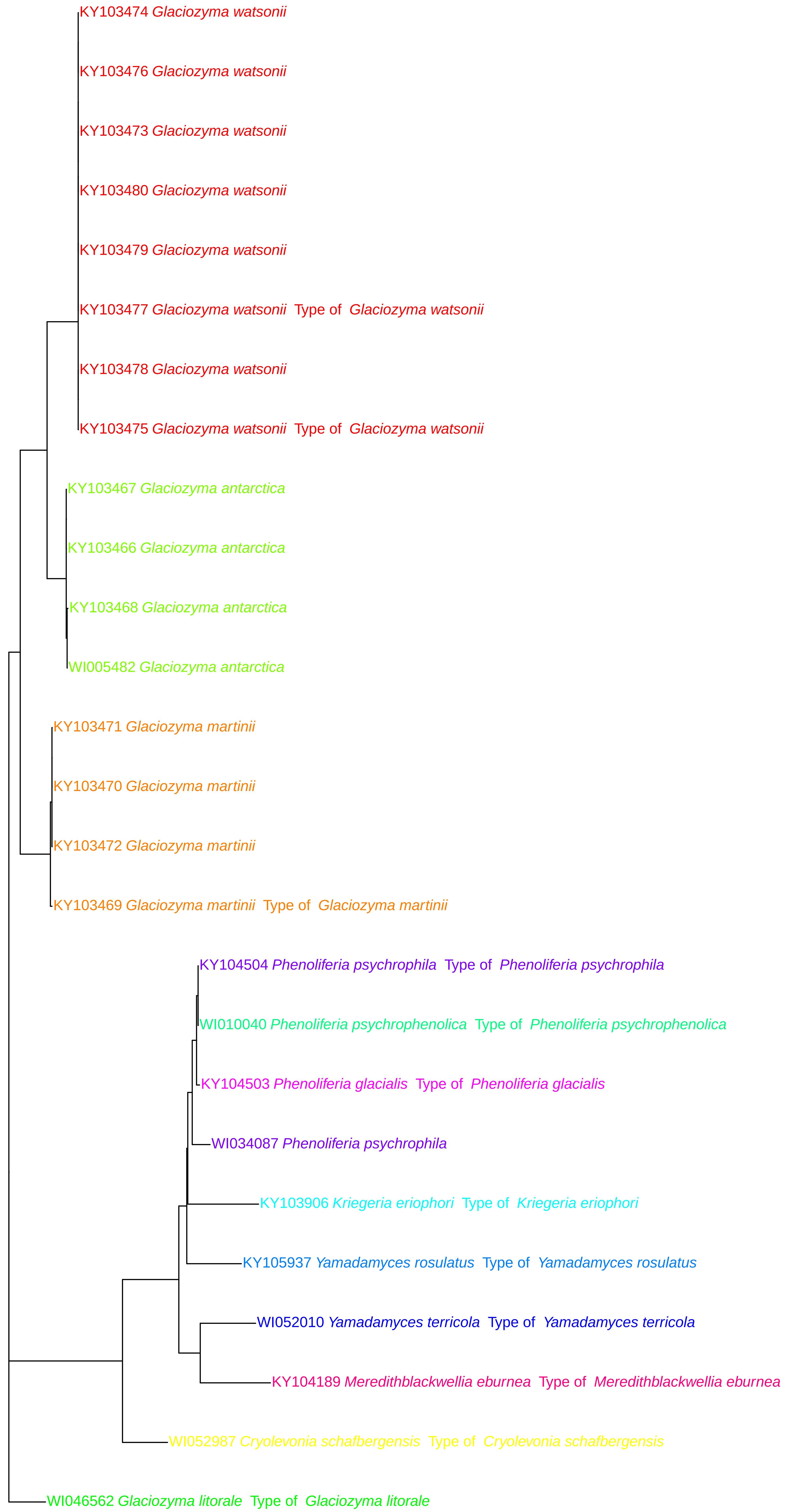

Leucosporidiales ITS

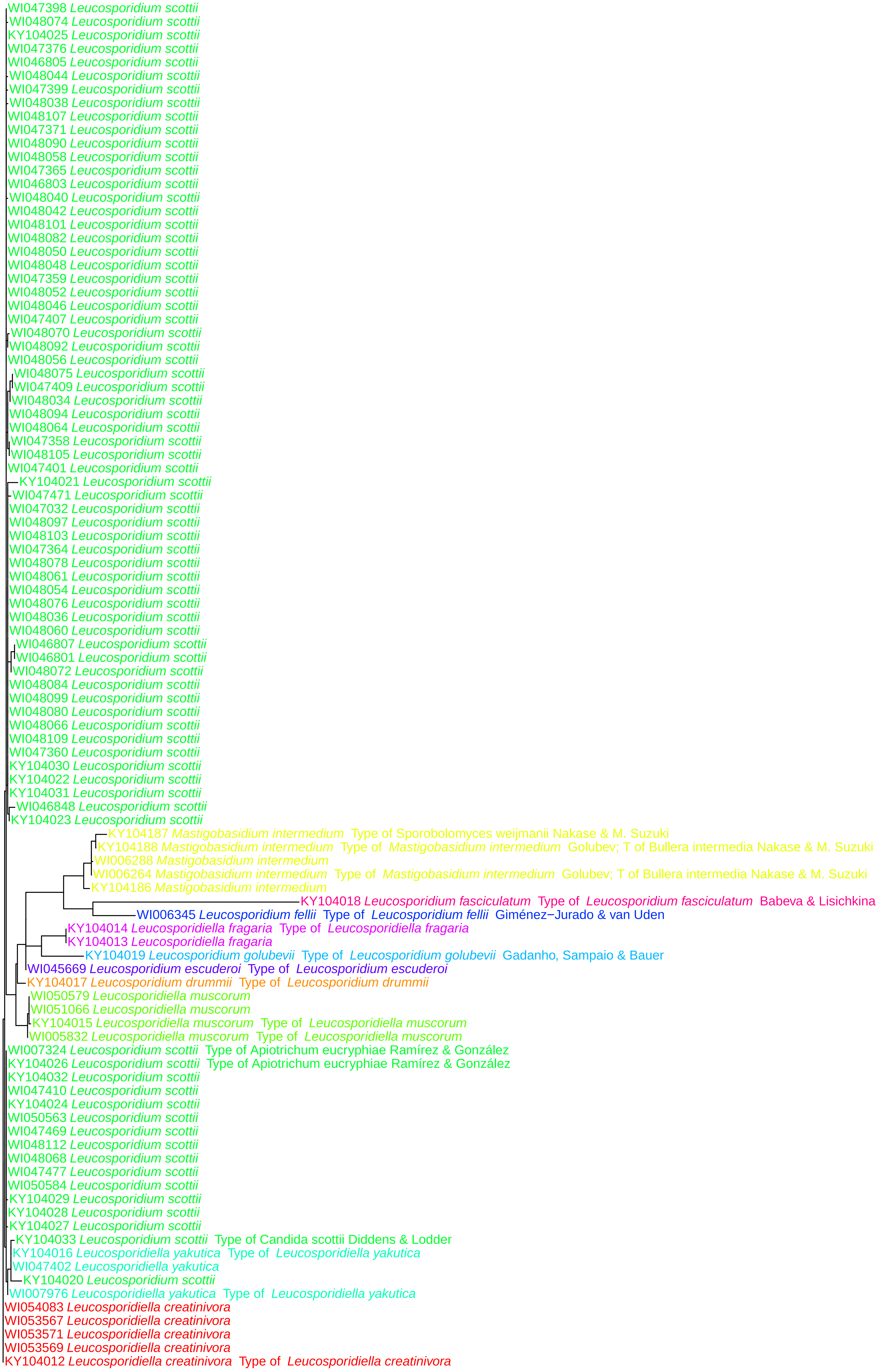

Lipomycetales ITS

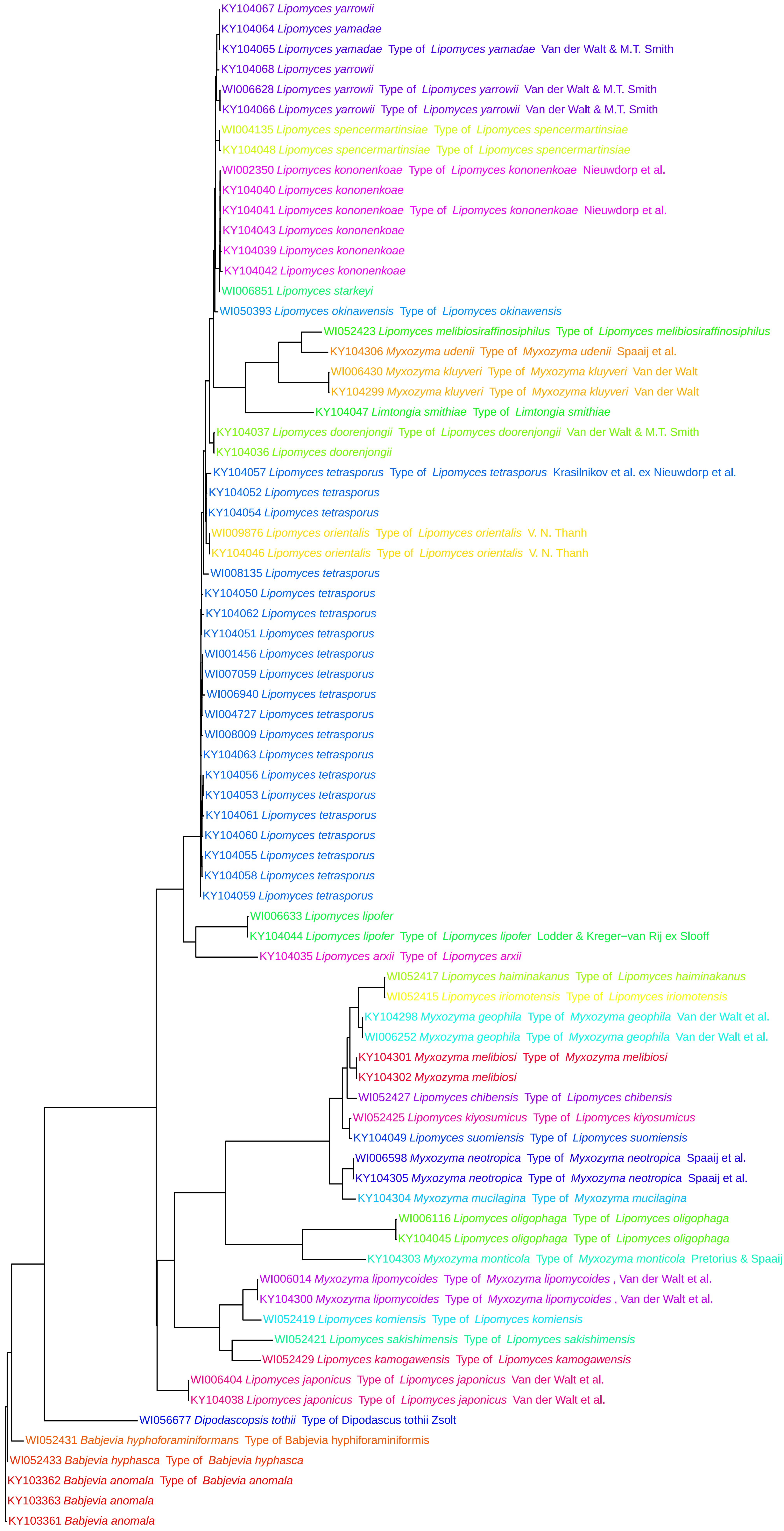

Malasseziales ITS

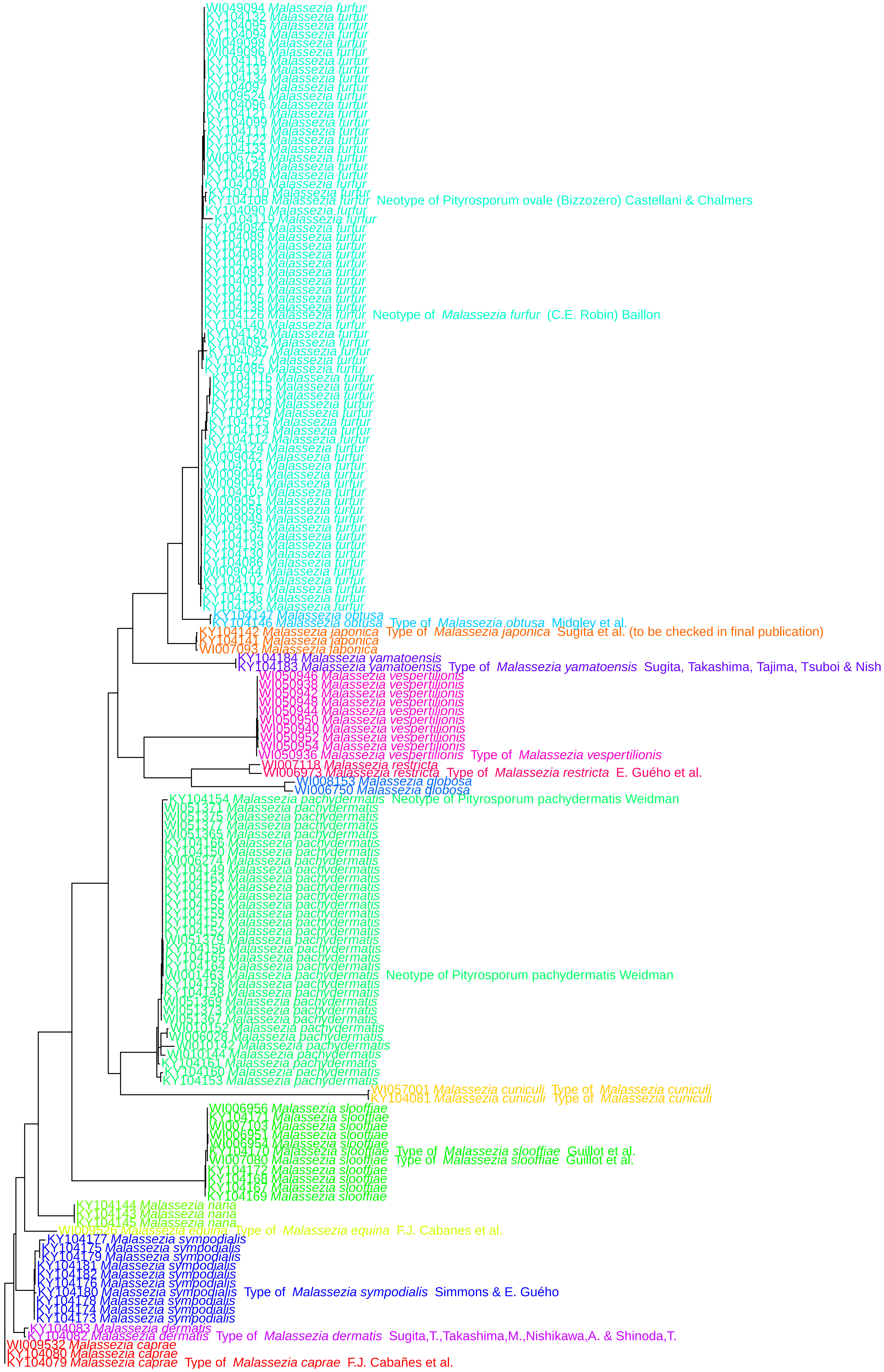

Microbotryales ITS

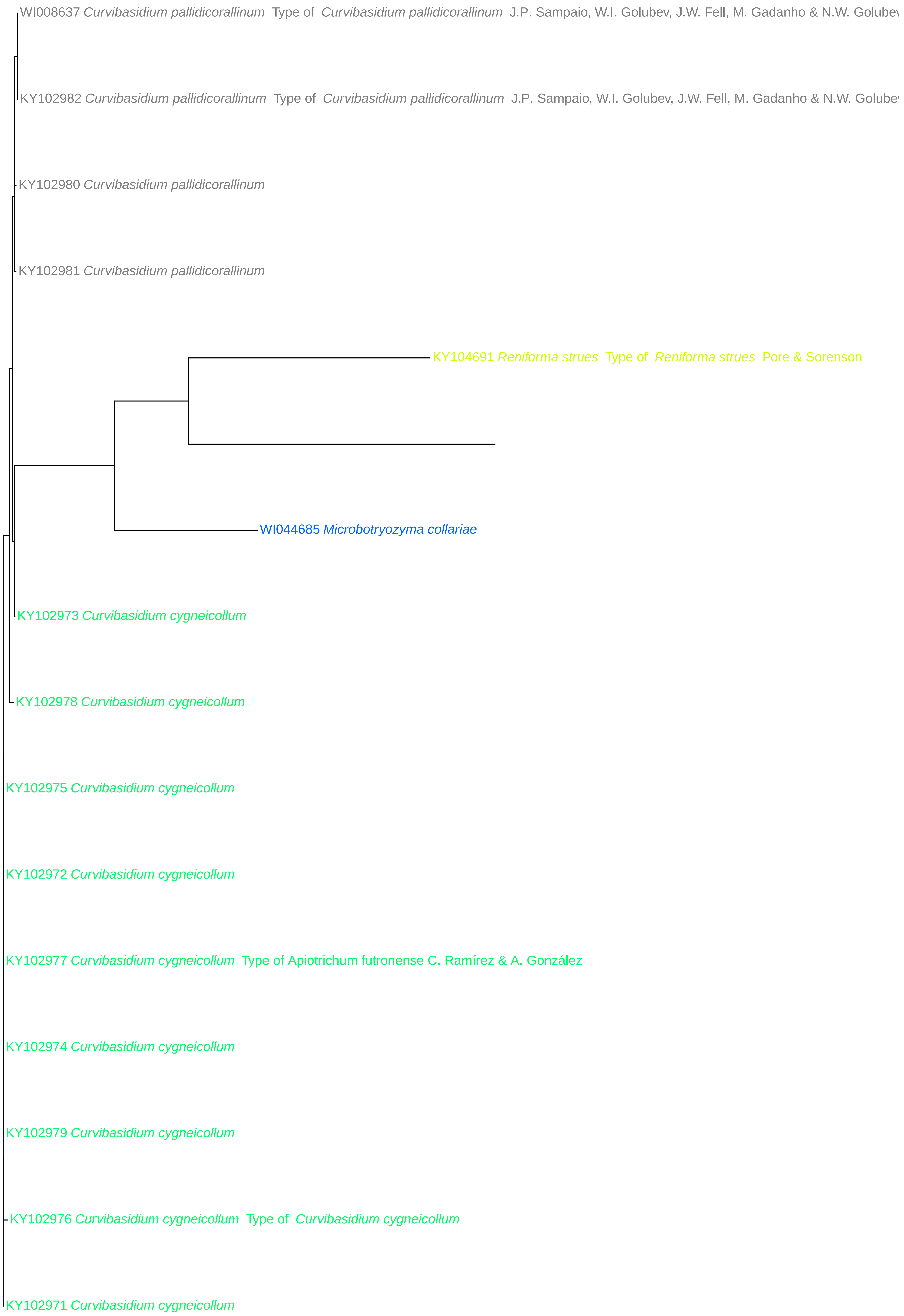

Microstromatales ITS

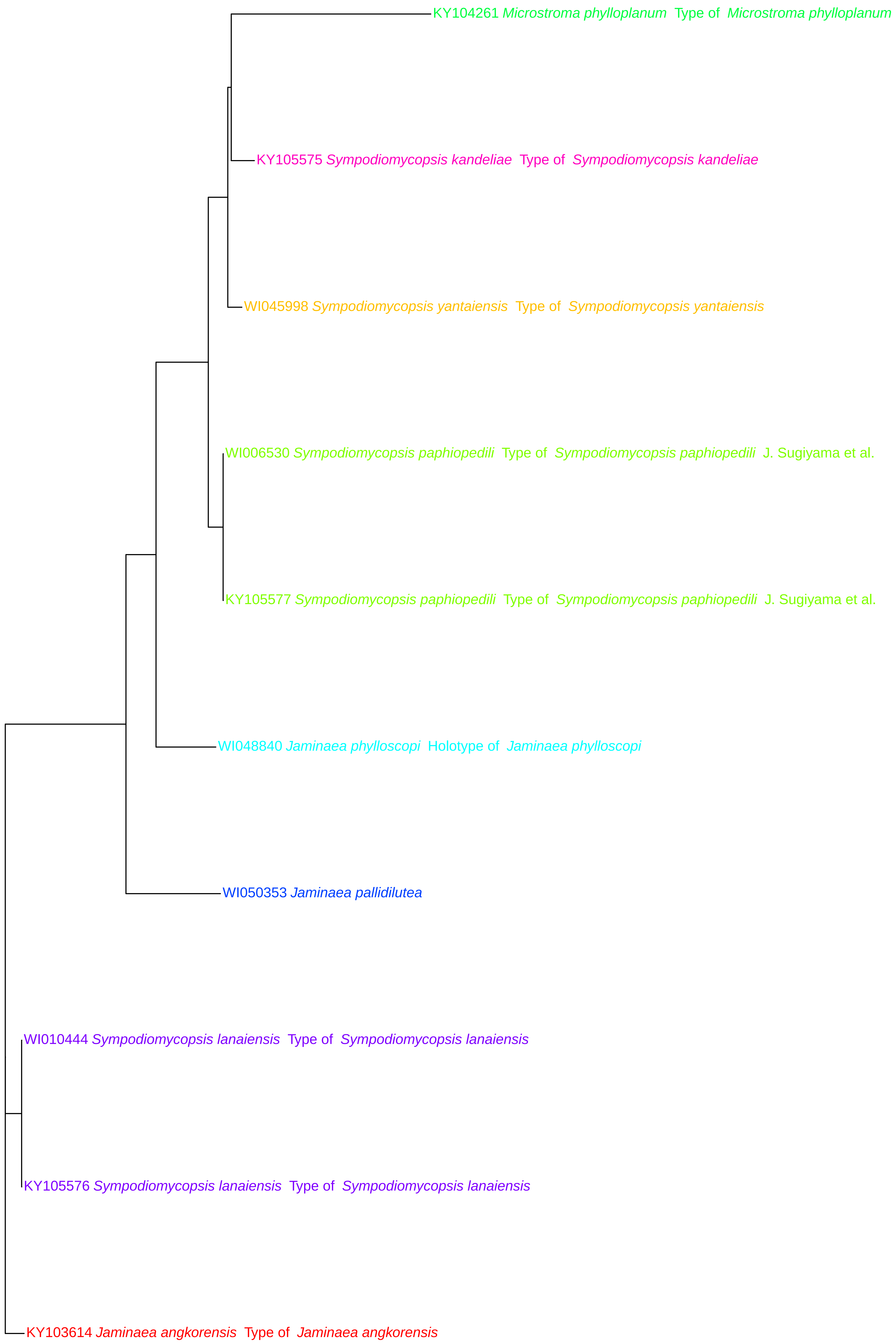

Moniliellales ITS

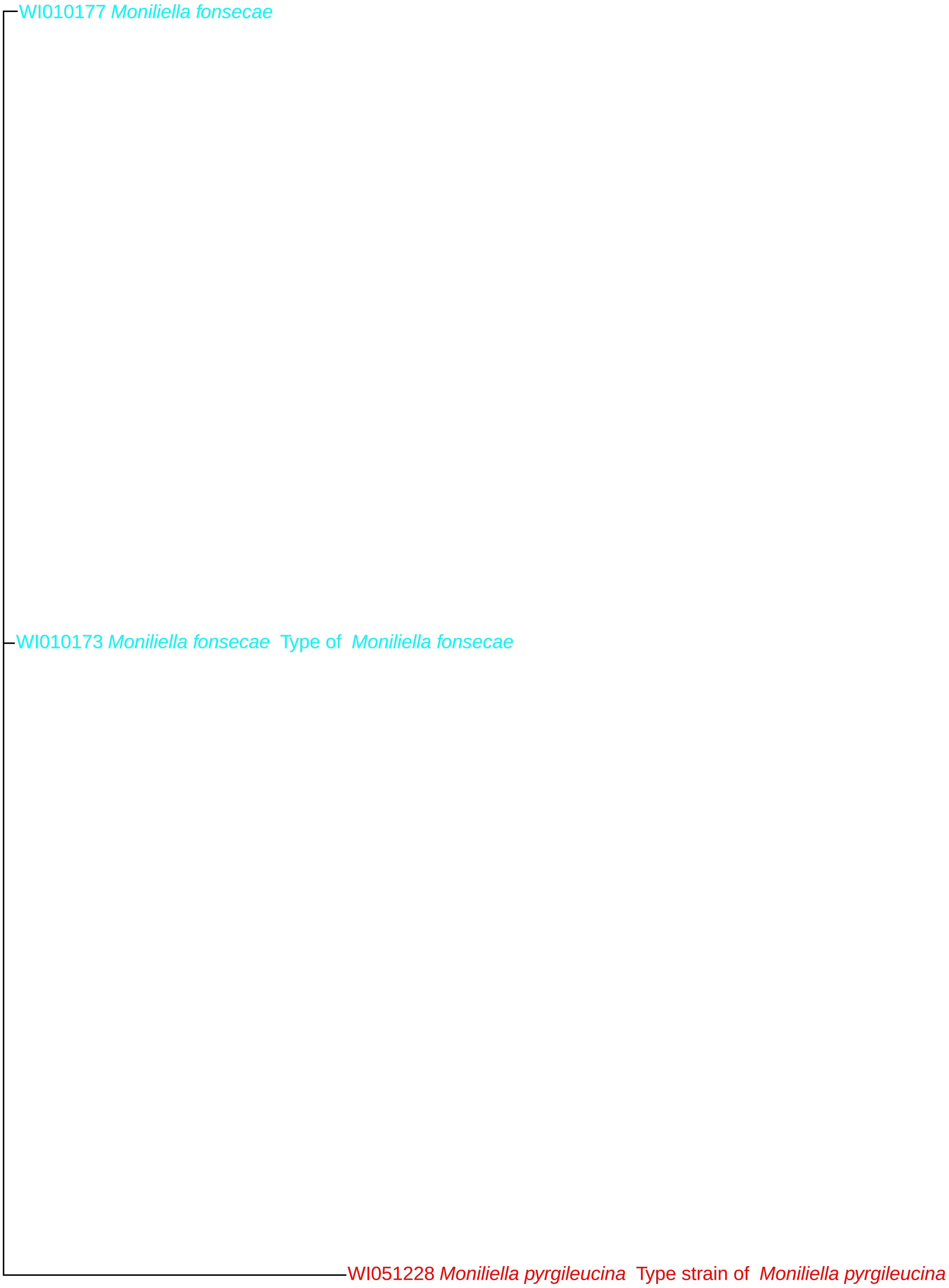

Phaffomycetales ITS

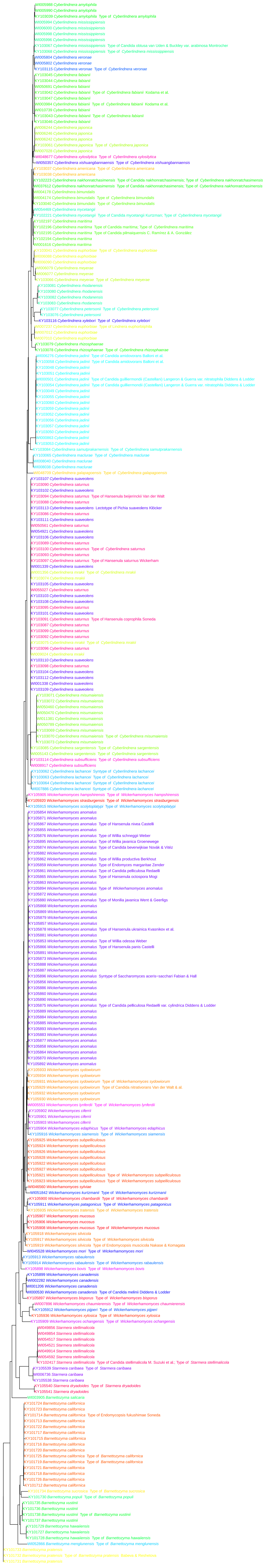

Pichiales ITS

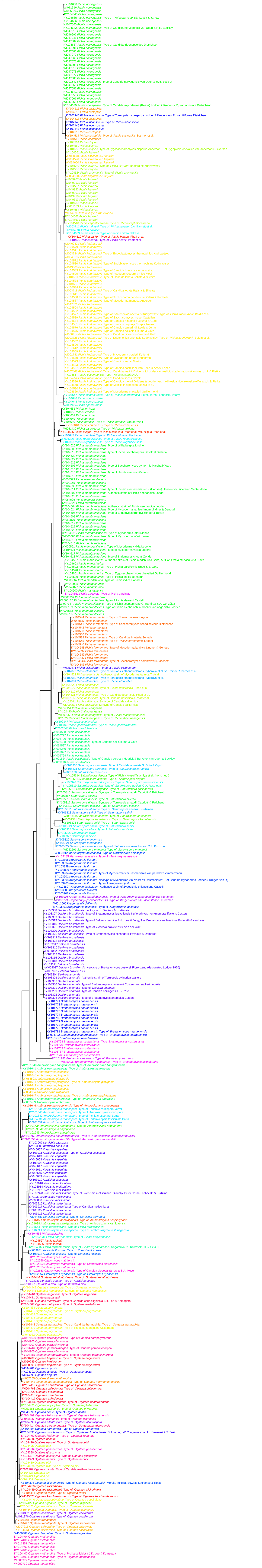

Saccharomycetales ITS

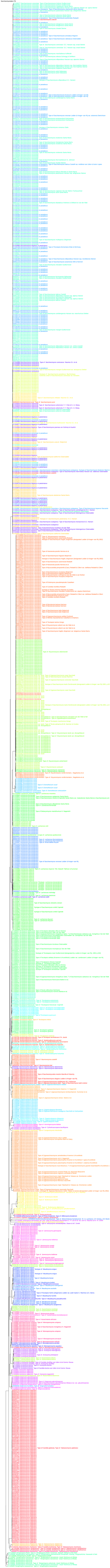

Saccharomycodales ITS

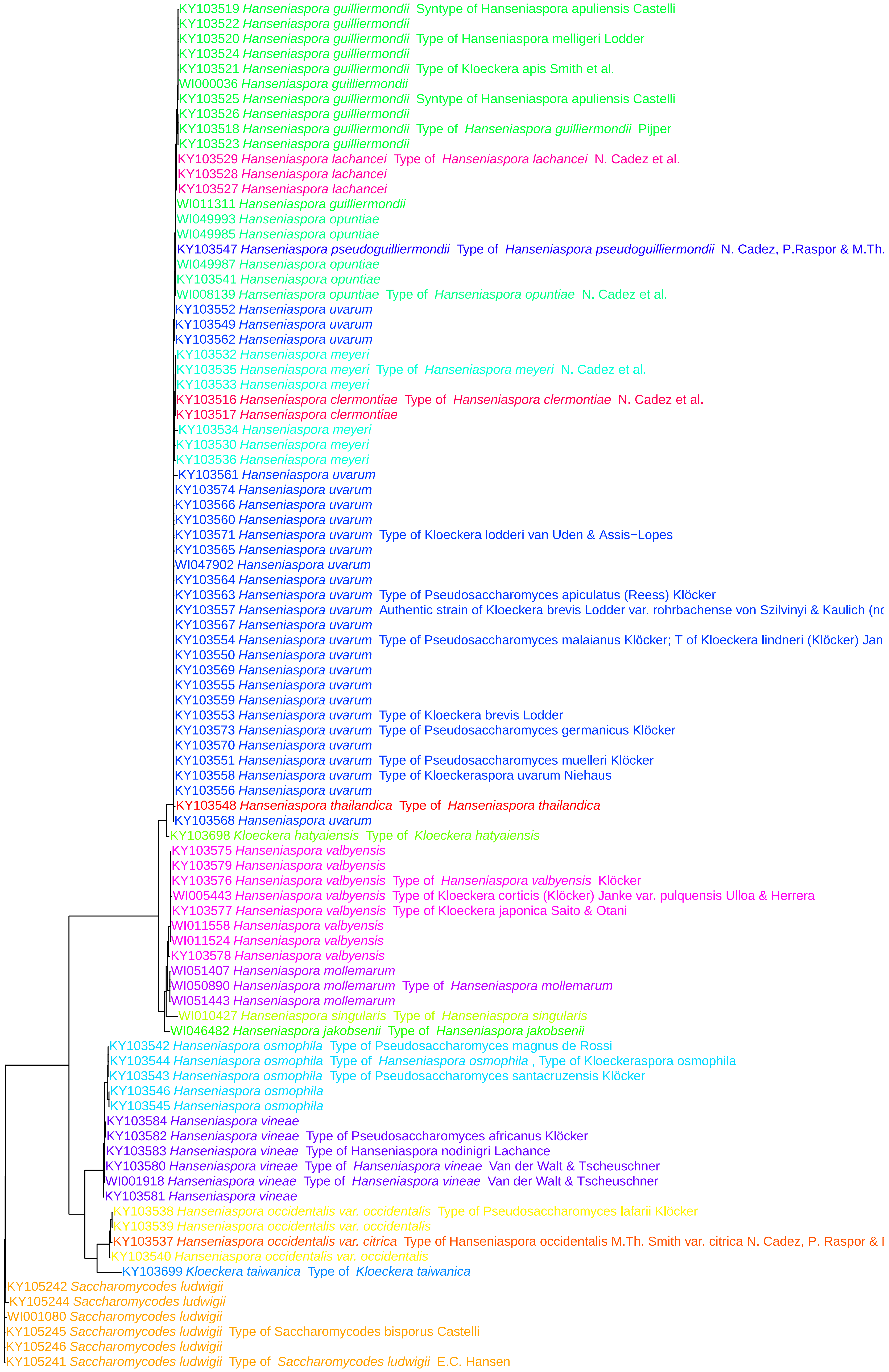

Sakaguchiales ITS

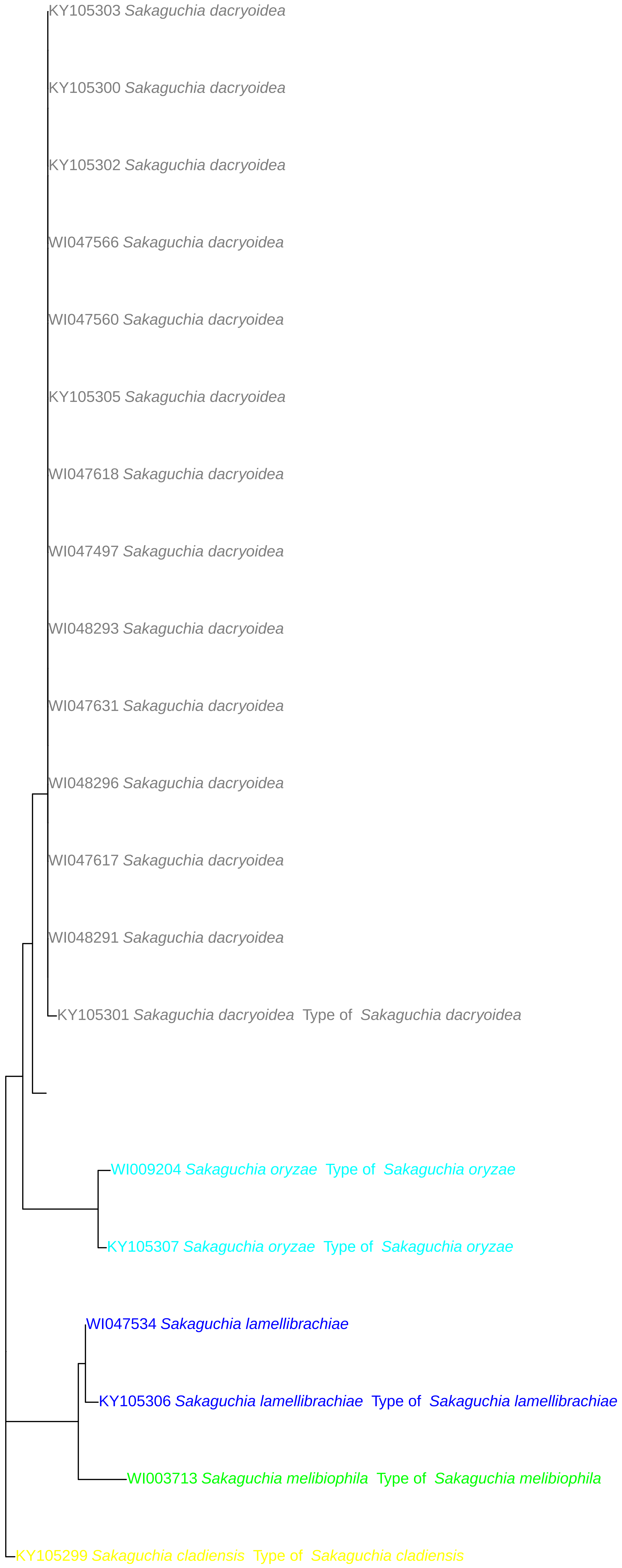

Schizosaccharomycetales ITS

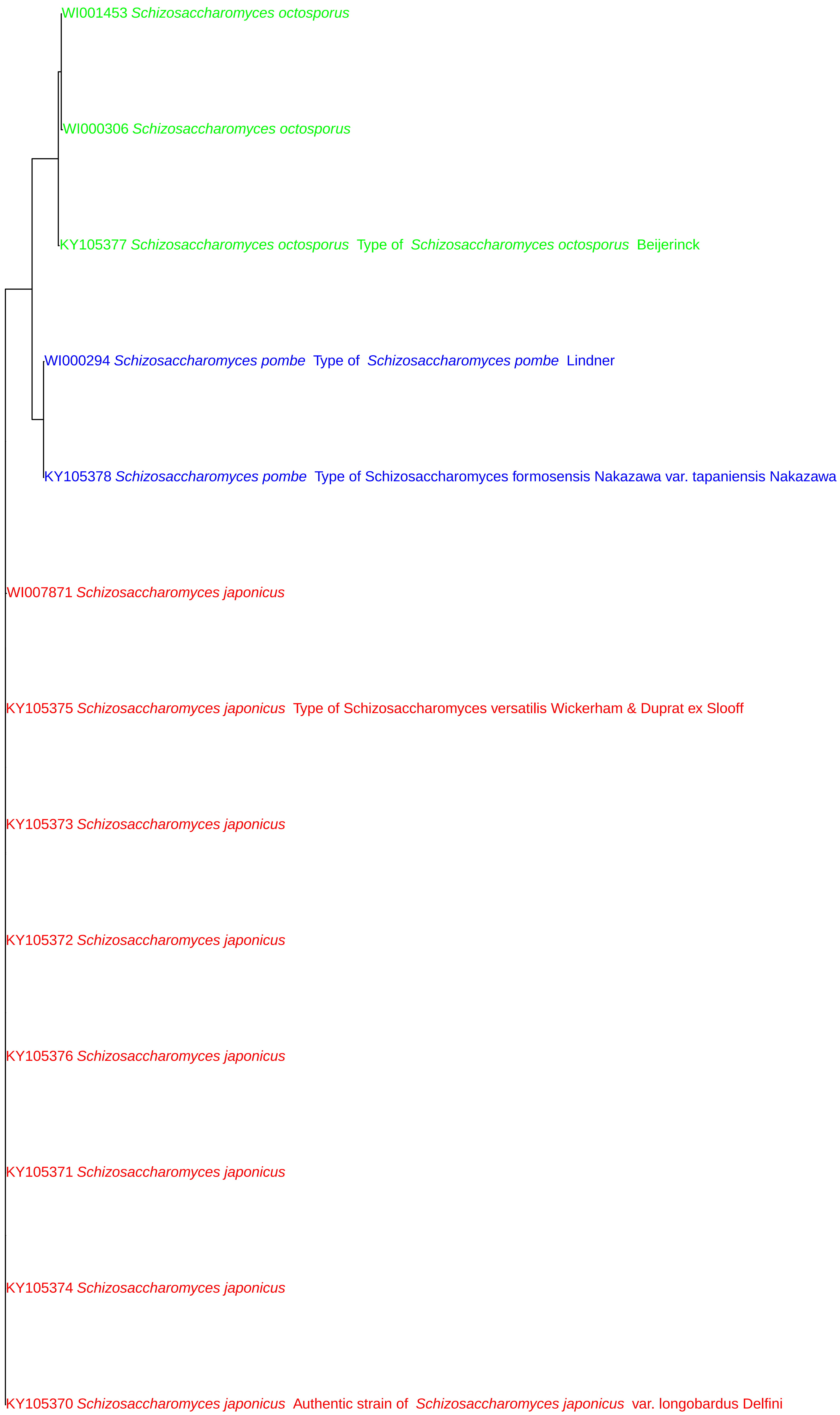

Serinales ITS

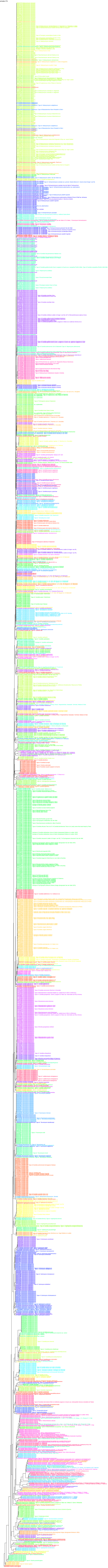

Spiculogloeales ITS

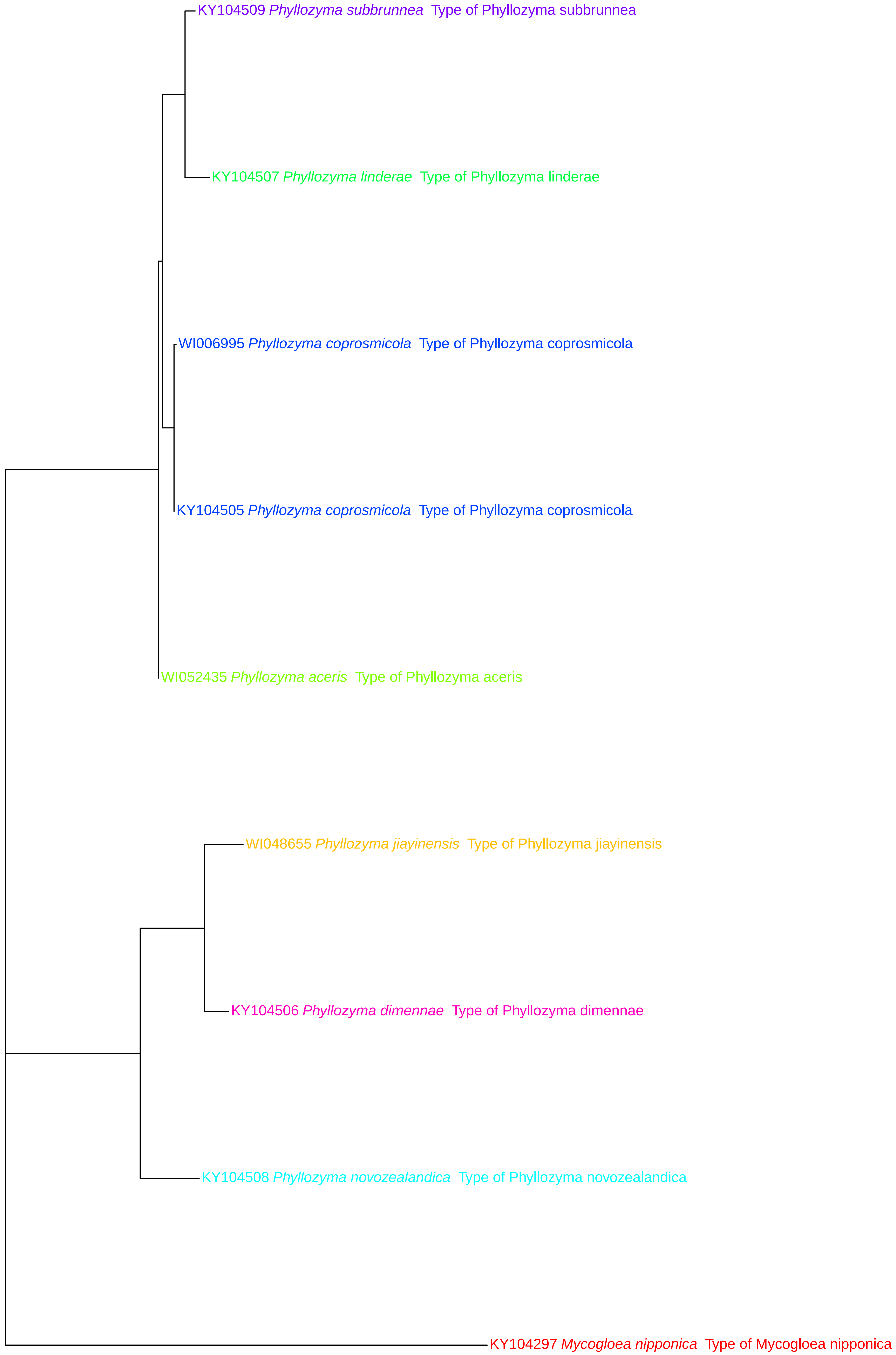

Sporidiobolales ITS

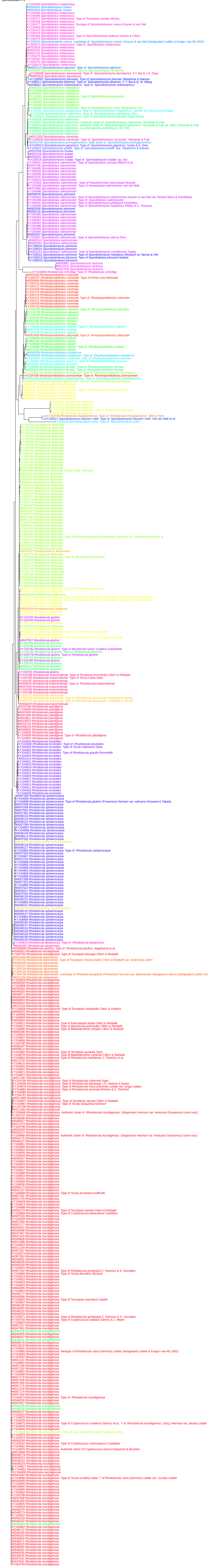

Sporopachydermiales ITS

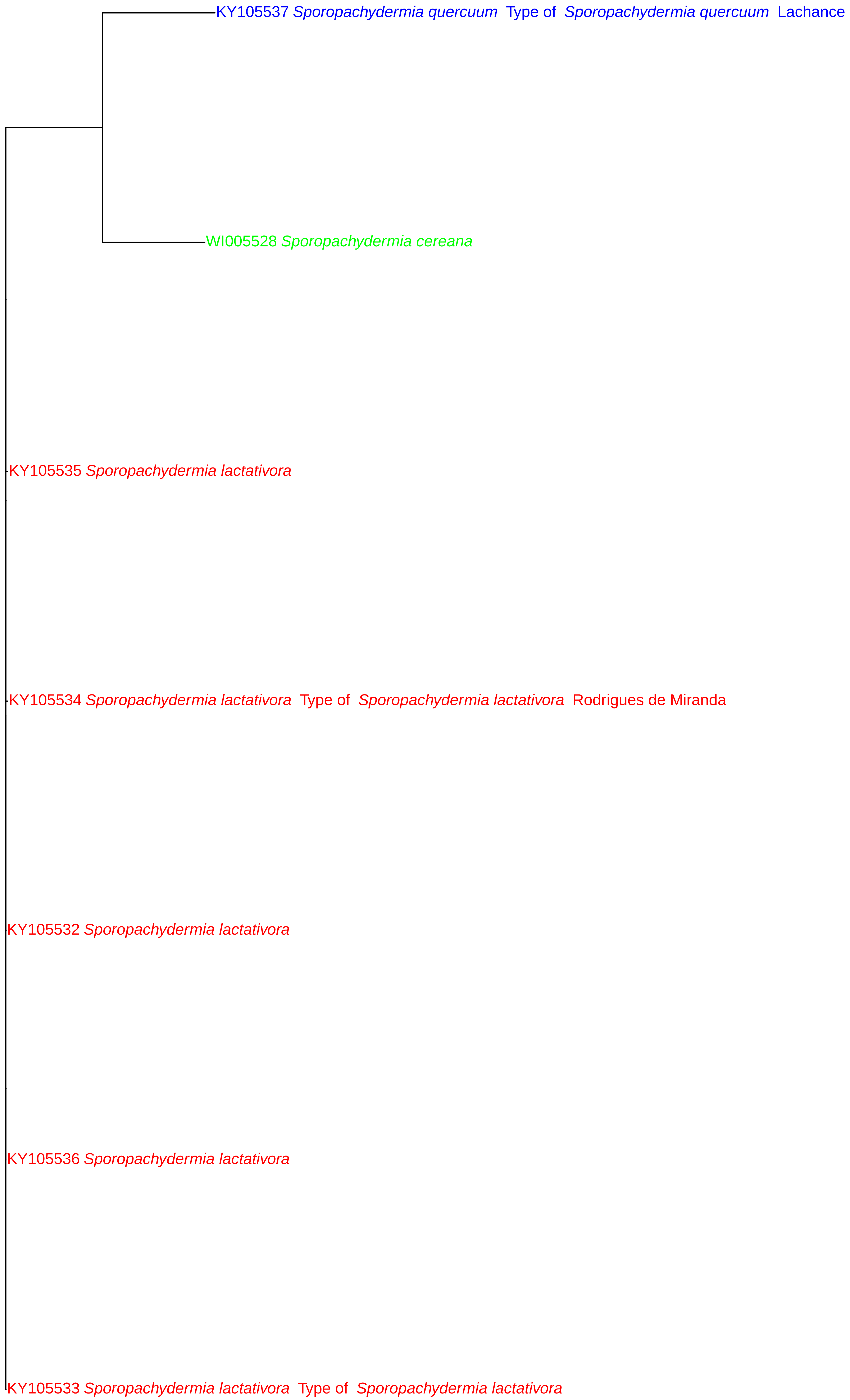

Taphrinales ITS

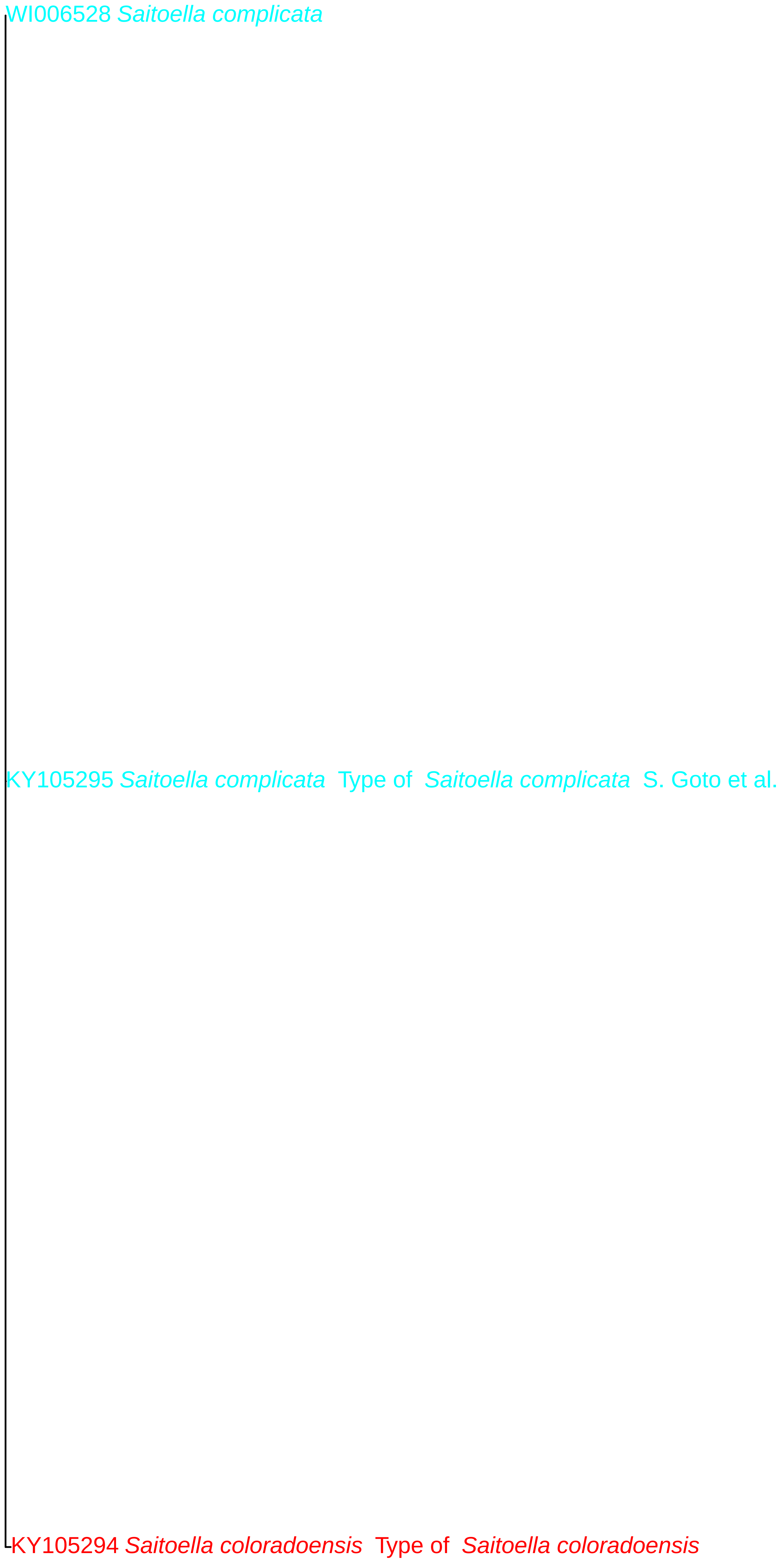

Tremellales ITS

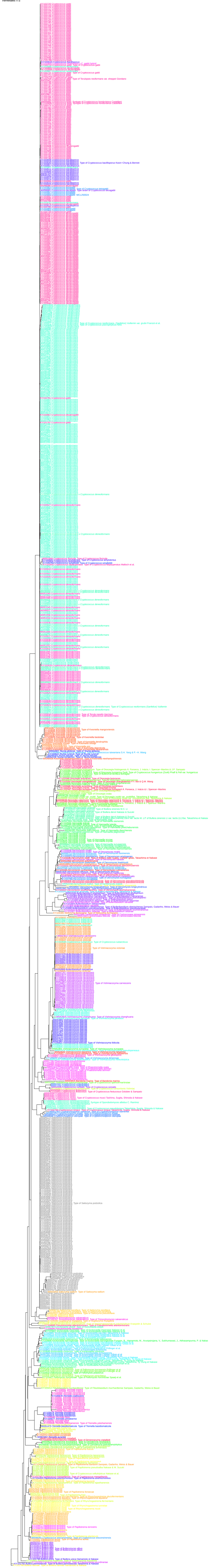

Trichosporonales ITS

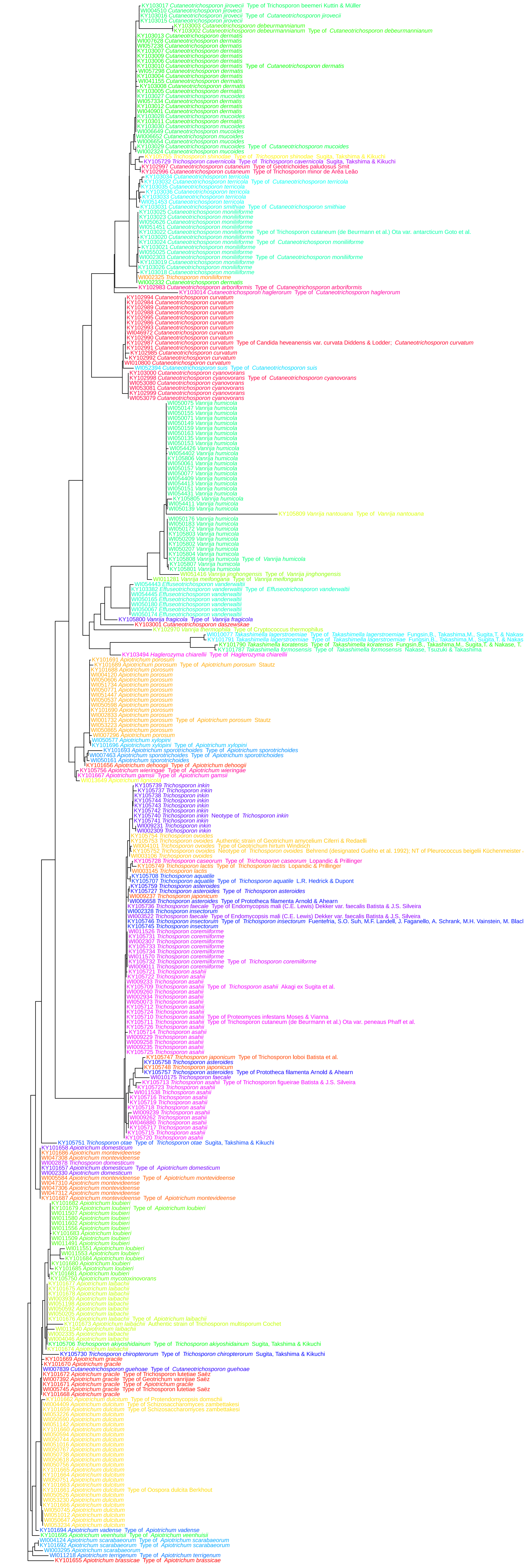

Trigonopsidales ITS

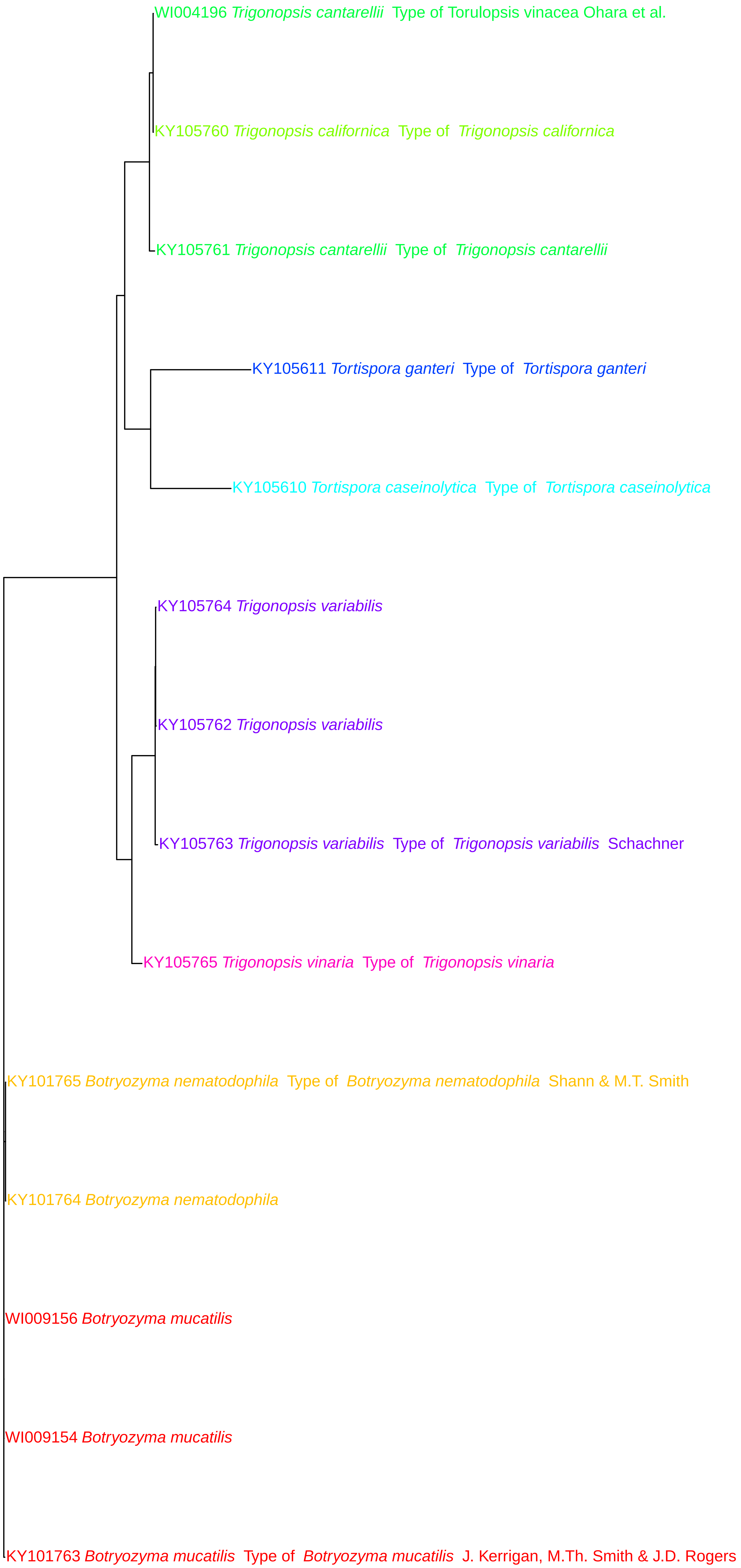

Ustilaginales ITS

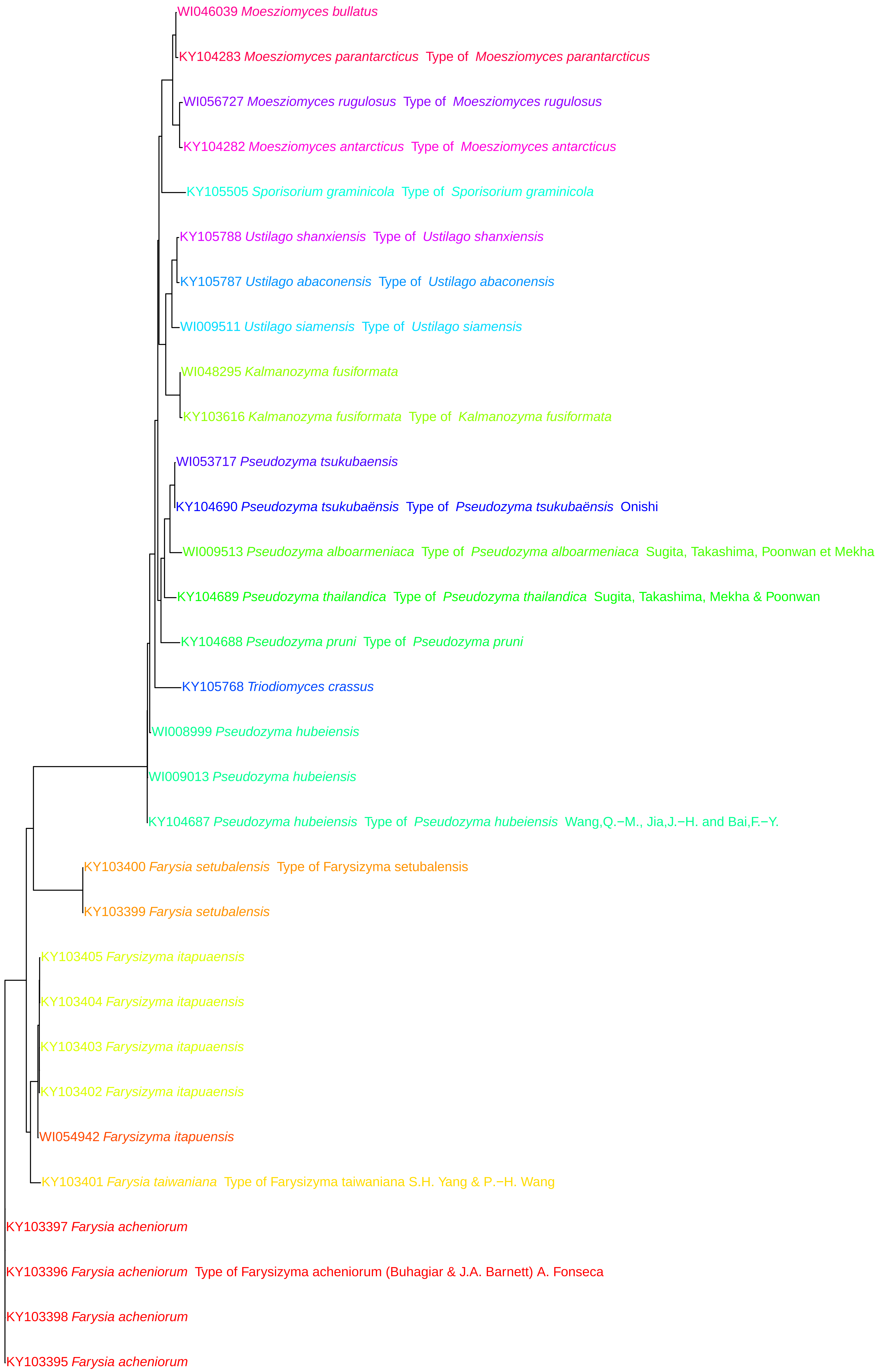

Supplement: Supplementary file 7 — Appendix S7: men70082‐sup‐0007‐AppendixS7.pdf. [file MEN-26-e70082-s009.pdf]
