# Supplementary material for: Advancing Yeast Identification Using High‐Throughput DNA Barcode Data From a Curated Culture Collection
Source: Mol Ecol Resour. 2025 Nov 26;26(1):e70082. doi: 10.1111/1755-0998.70082 (PMC12649295; doi:10.1111/1755-0998.70082)

Agaricostilbales LSU

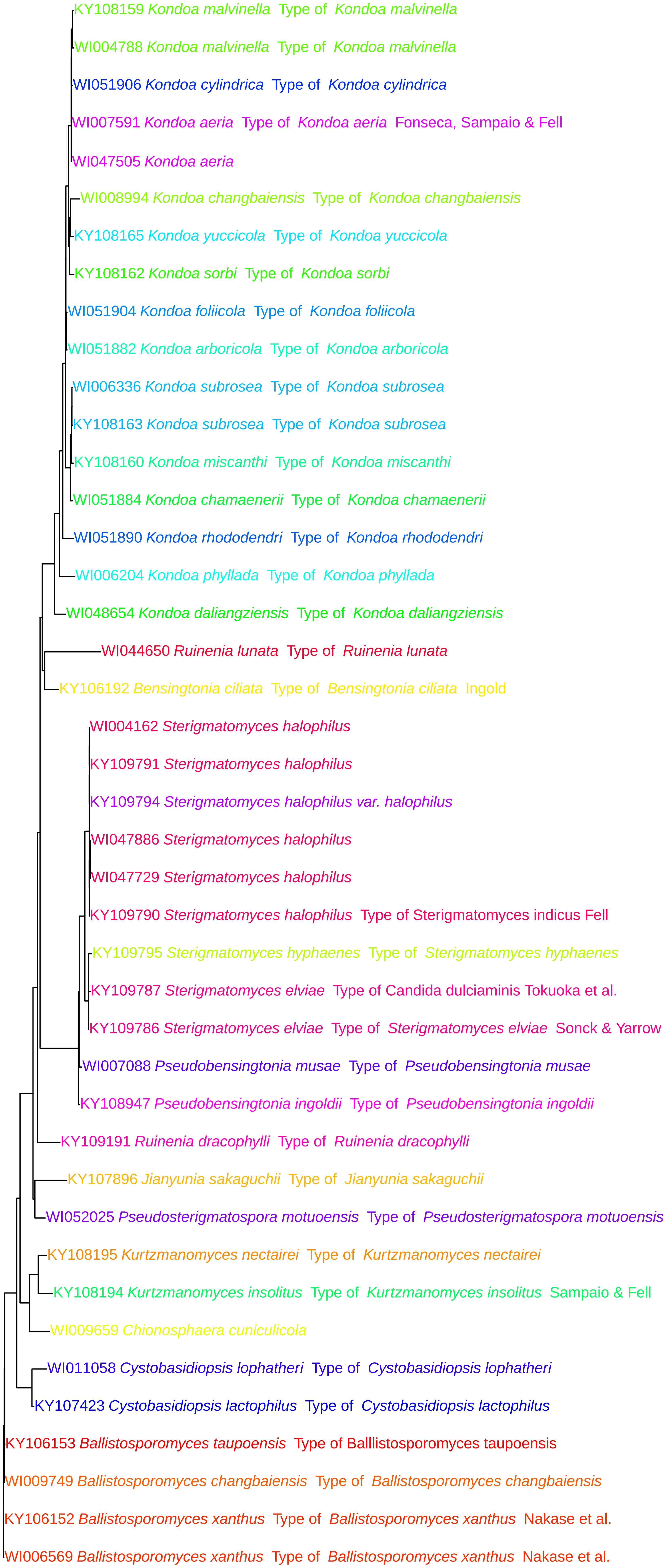

Alaninales LSU

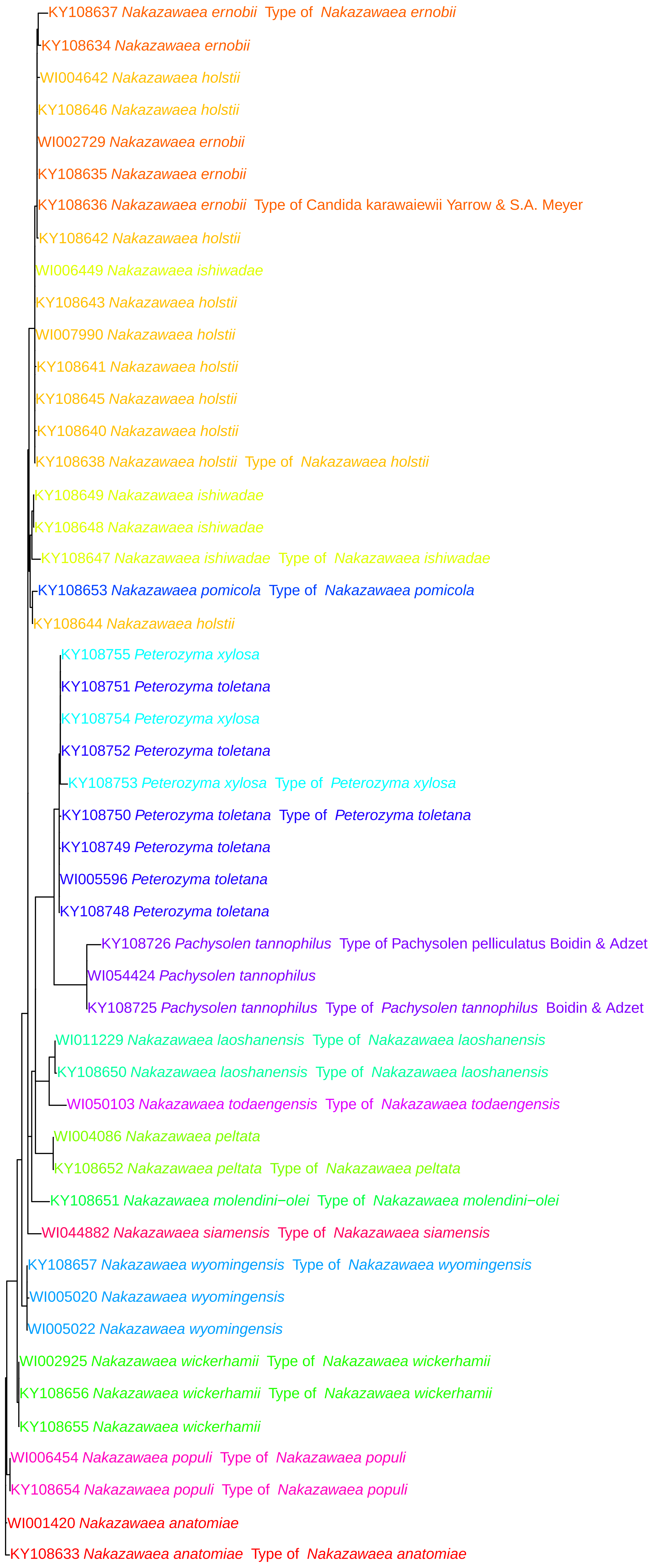

Ascoideales LSU

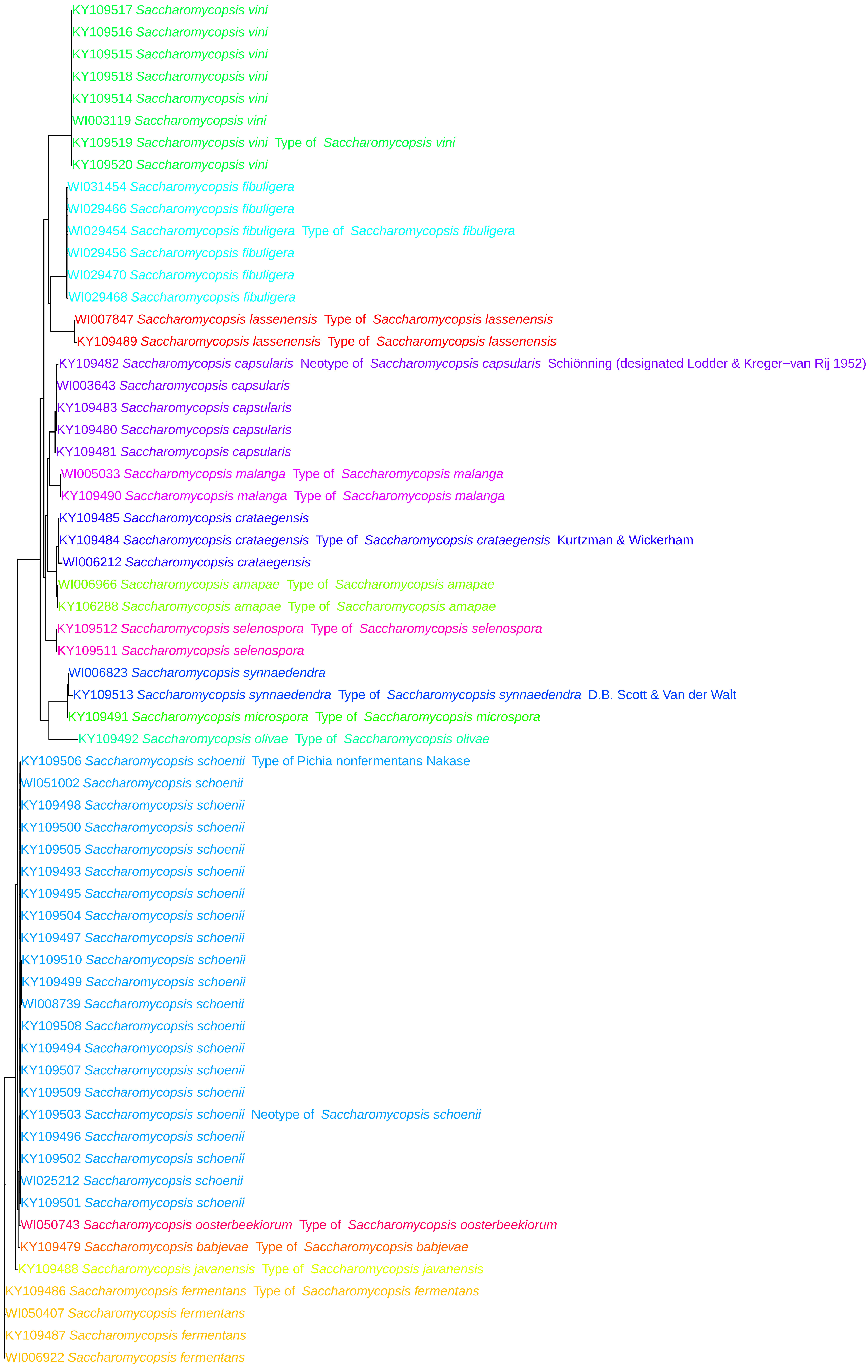

Buckleyzymales LSU

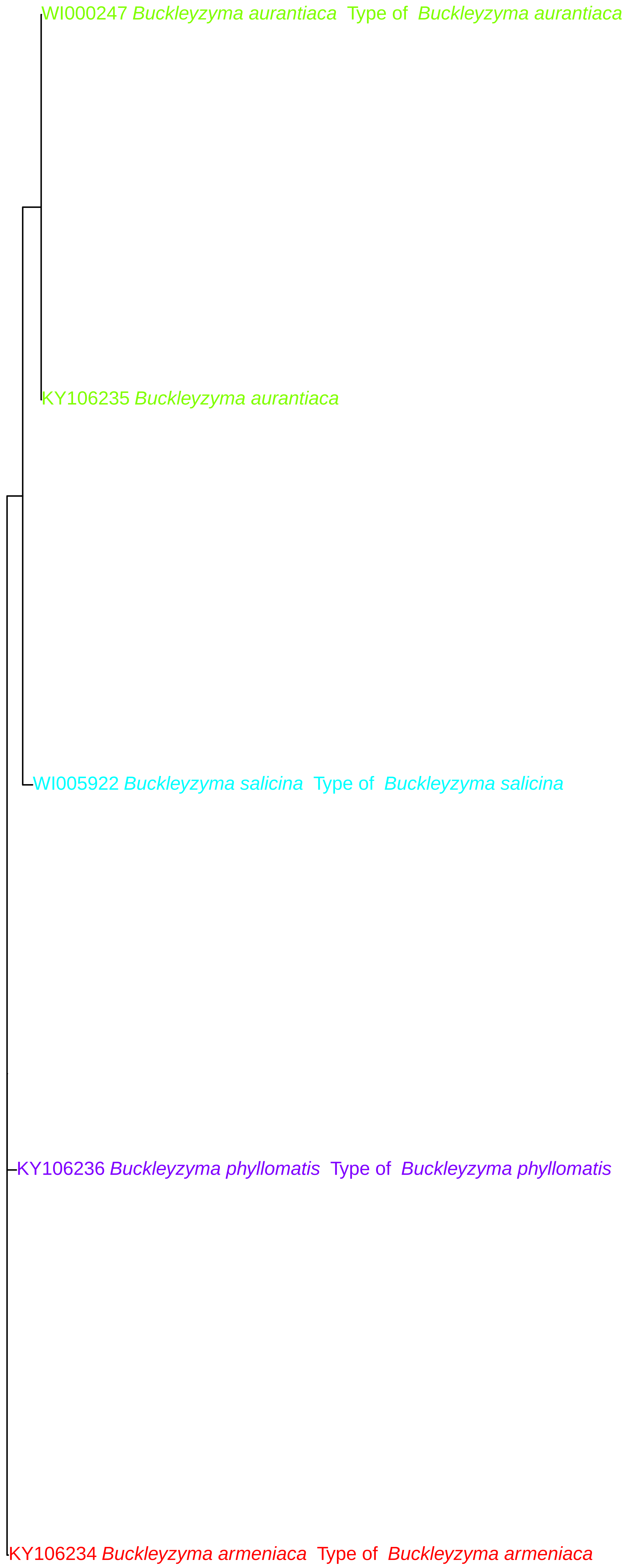

Cystobasidiales LSU

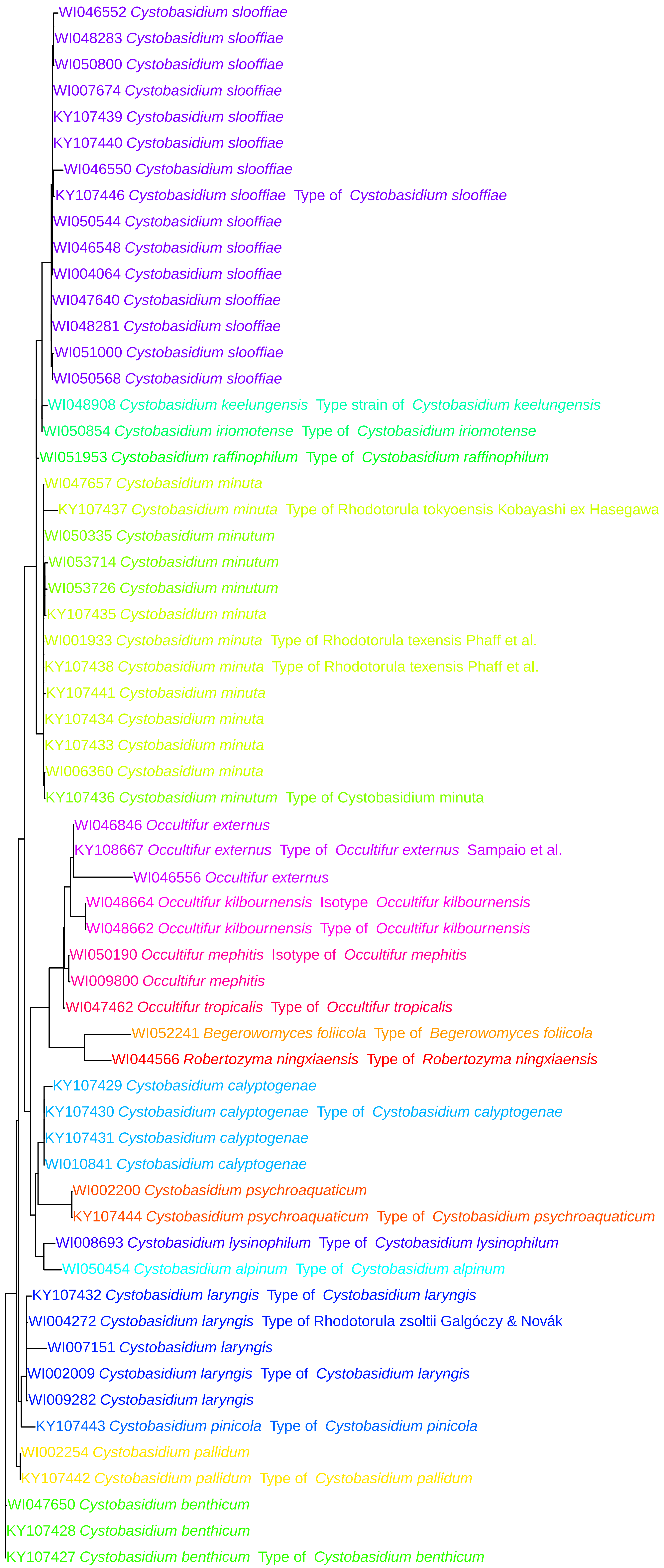

Cystofilobasidiales LSU

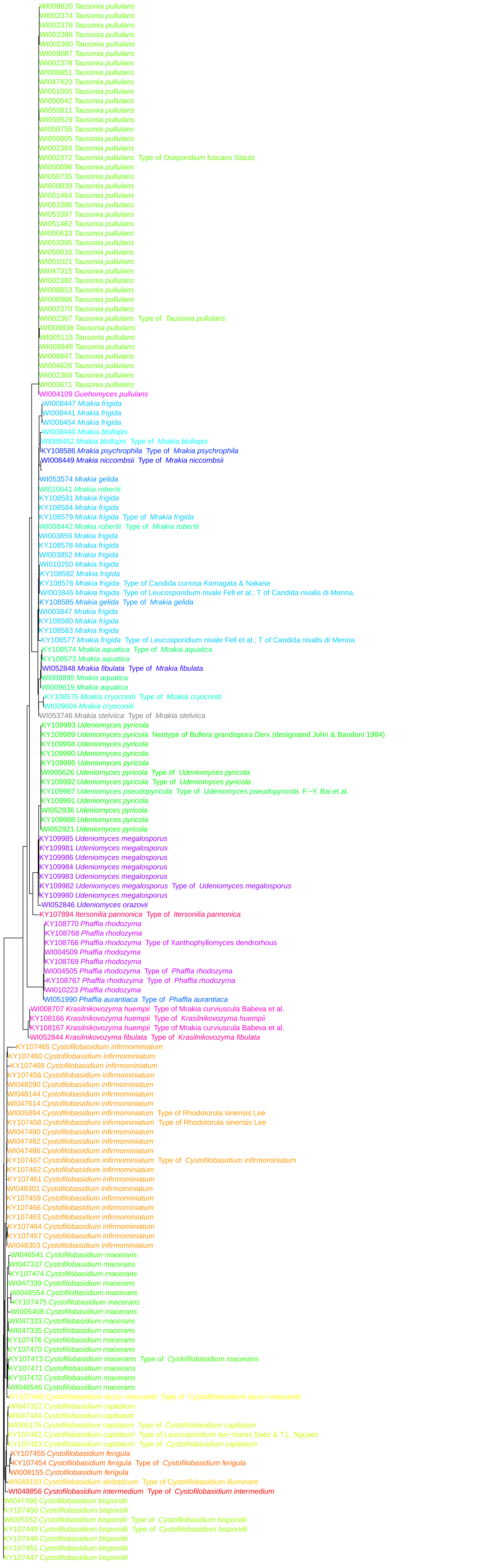

Dipodascales LSU

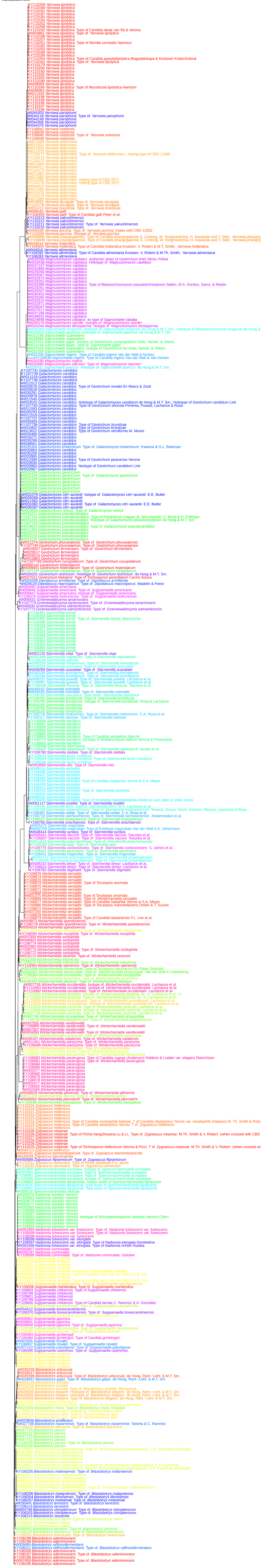

Filobasidiales LSU

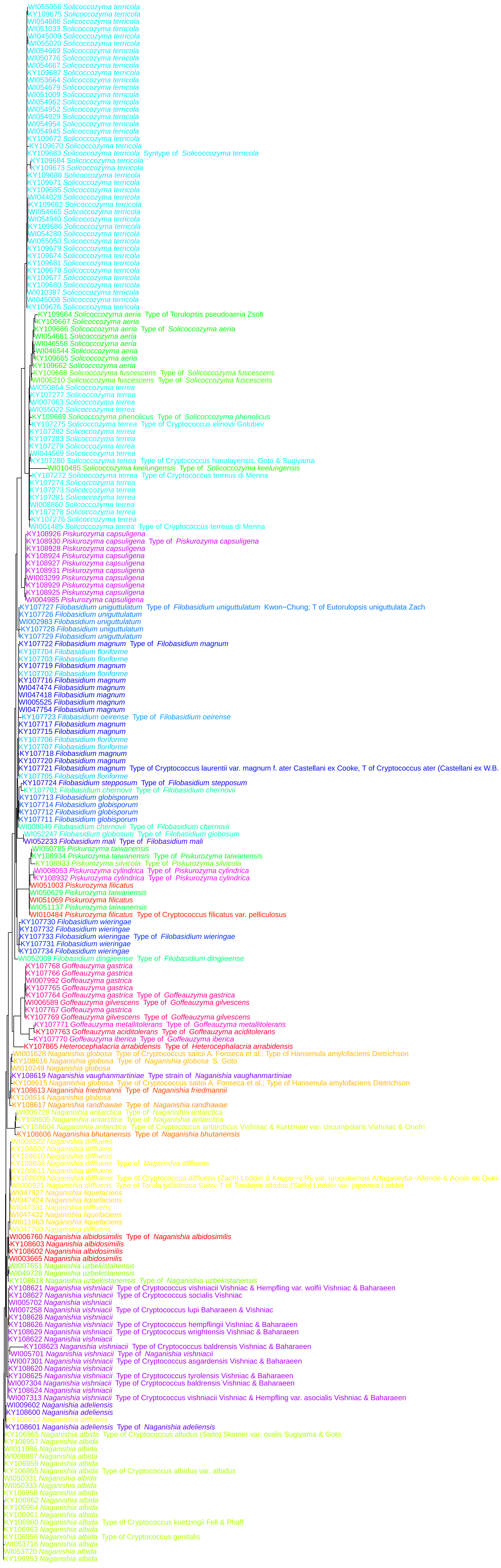

Heitmaniales LSU

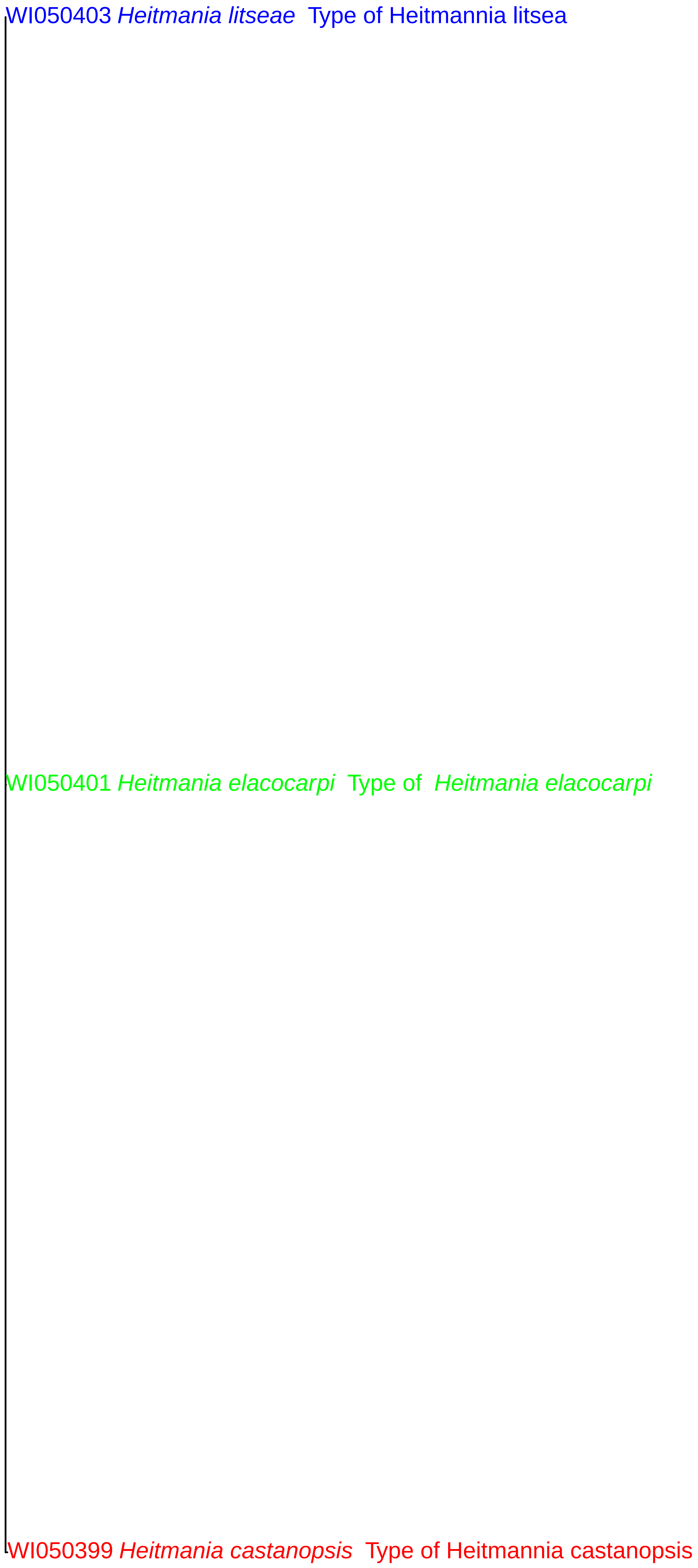

Heterogastridiales LSU

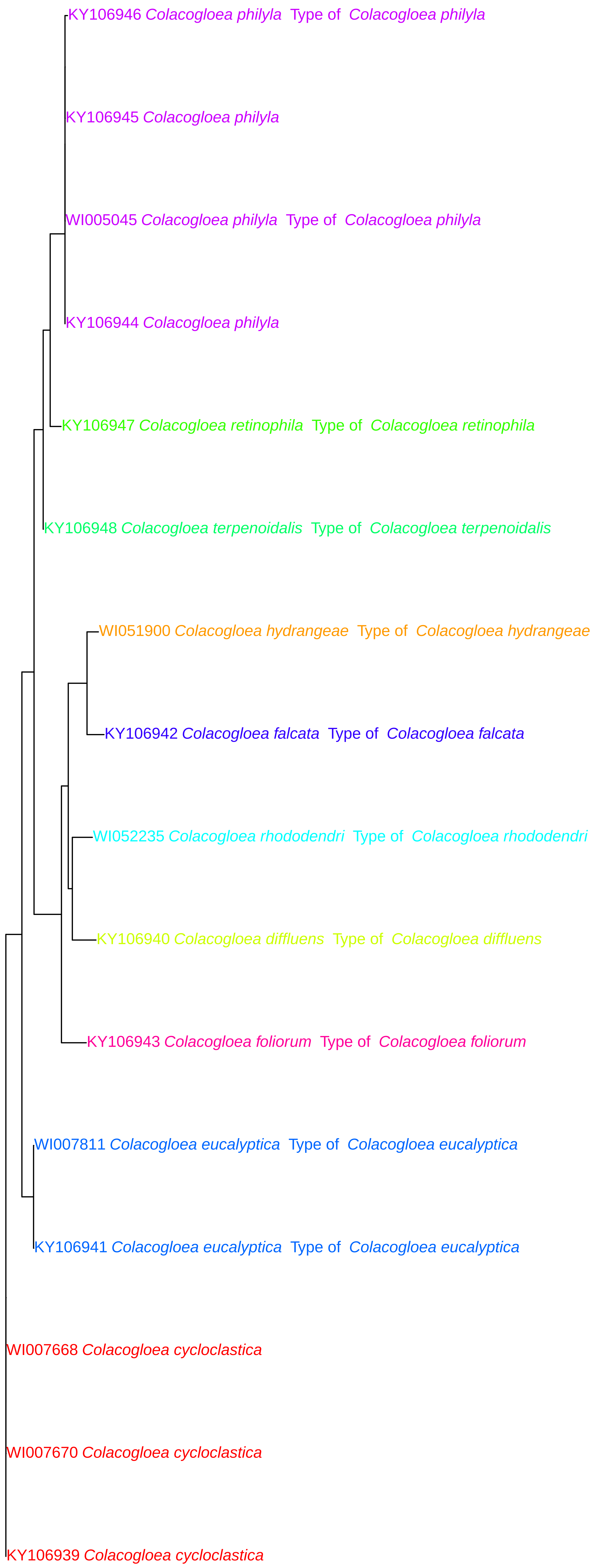

Holtermanniales LSU

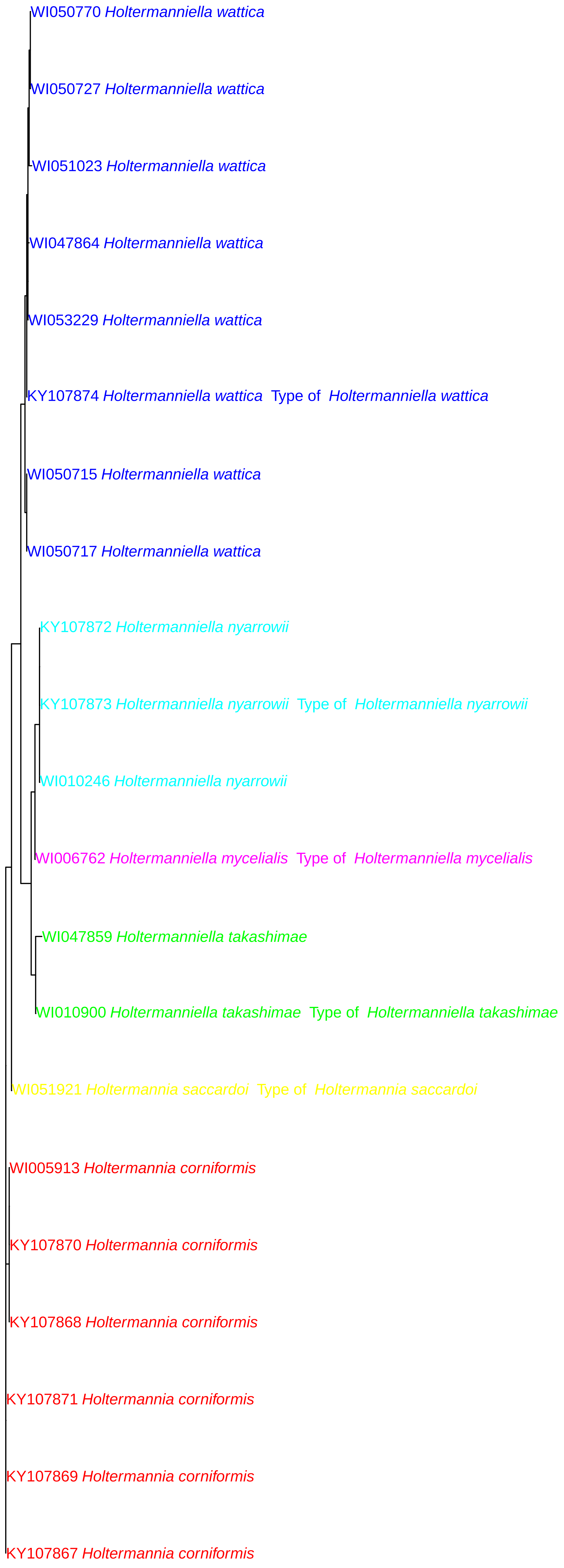

Kriegeriales LSU

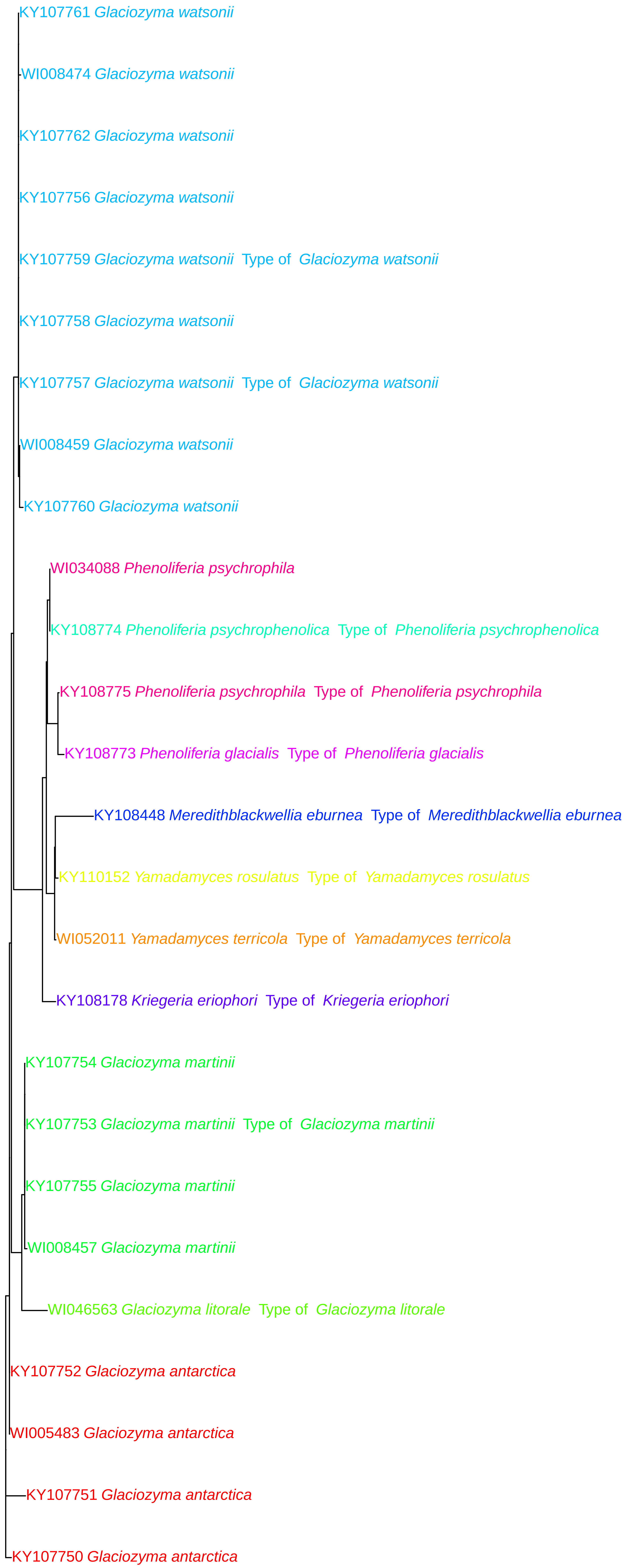

Leucosporidiales LSU

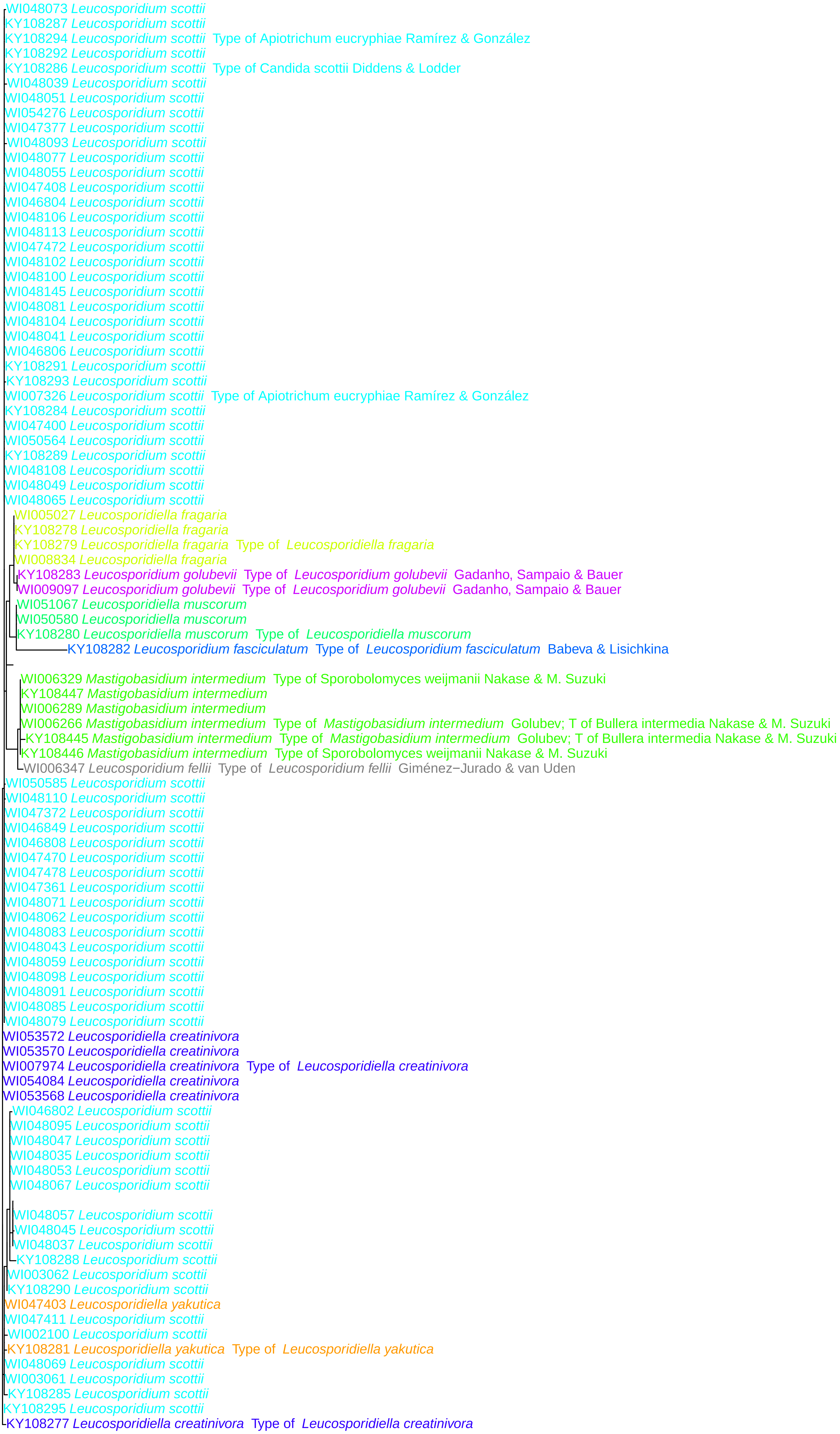

Lipomycetales LSU

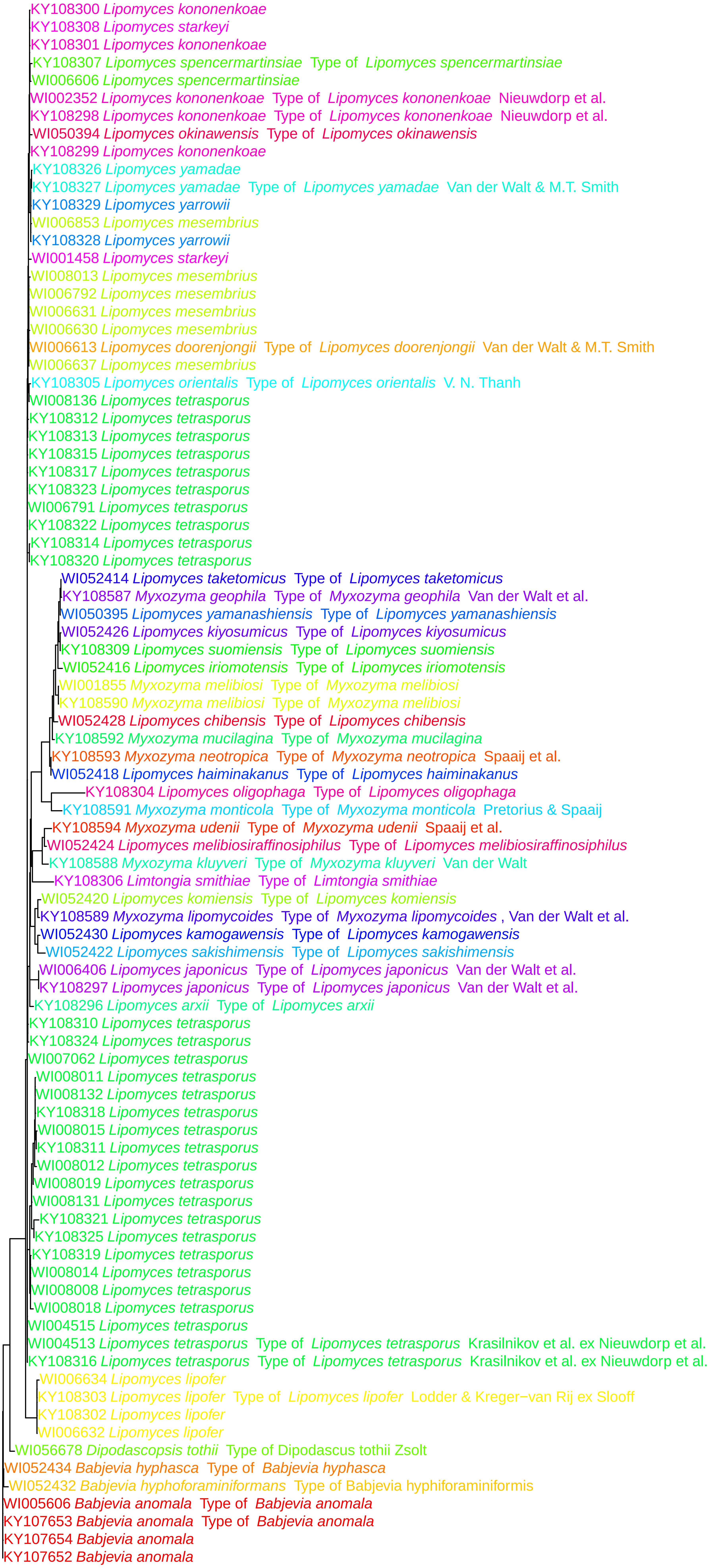

Malasseziales LSU

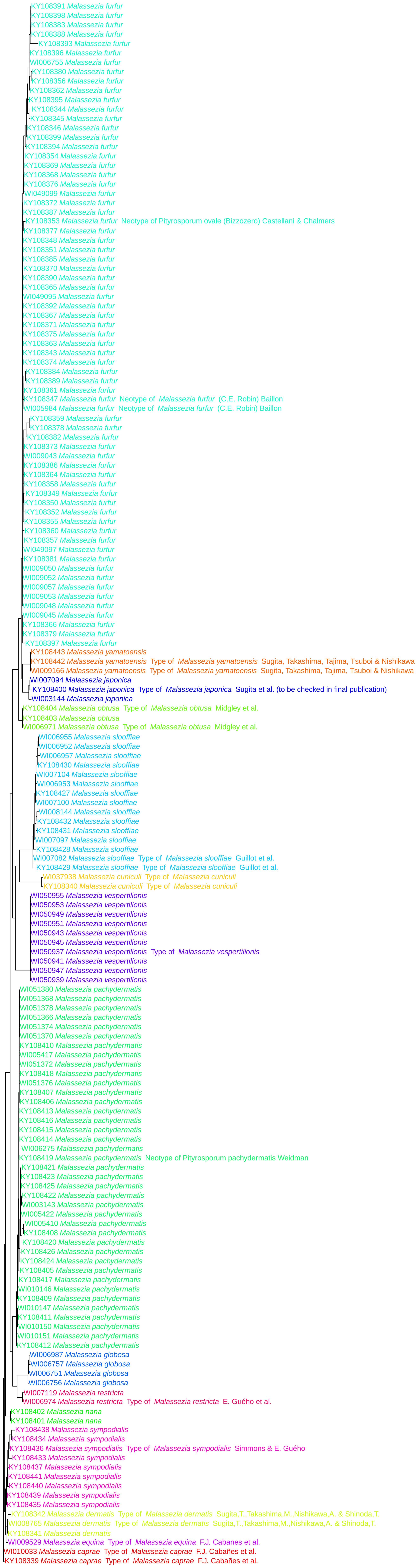

Microbotryales LSU

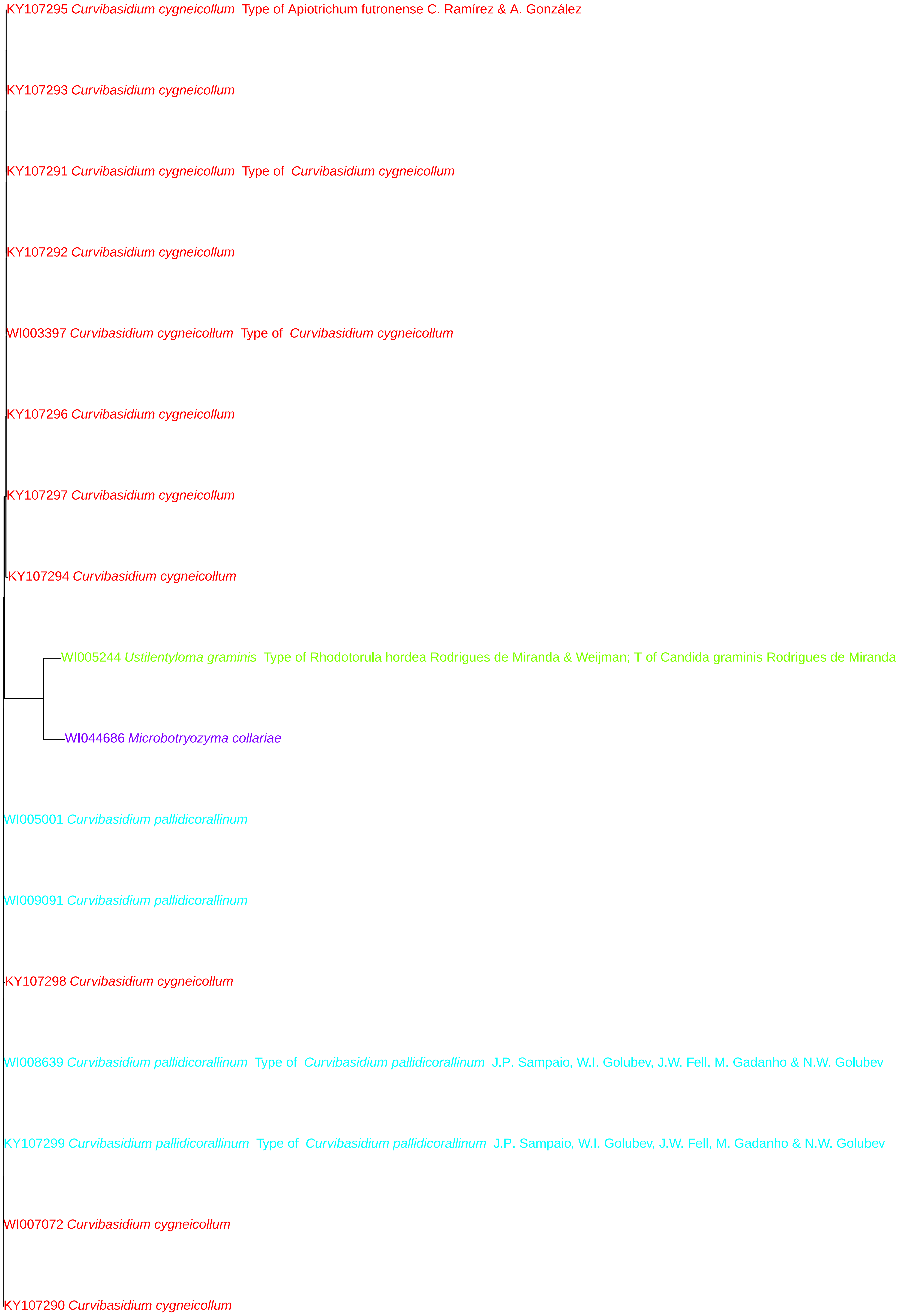

Microstromatales LSU

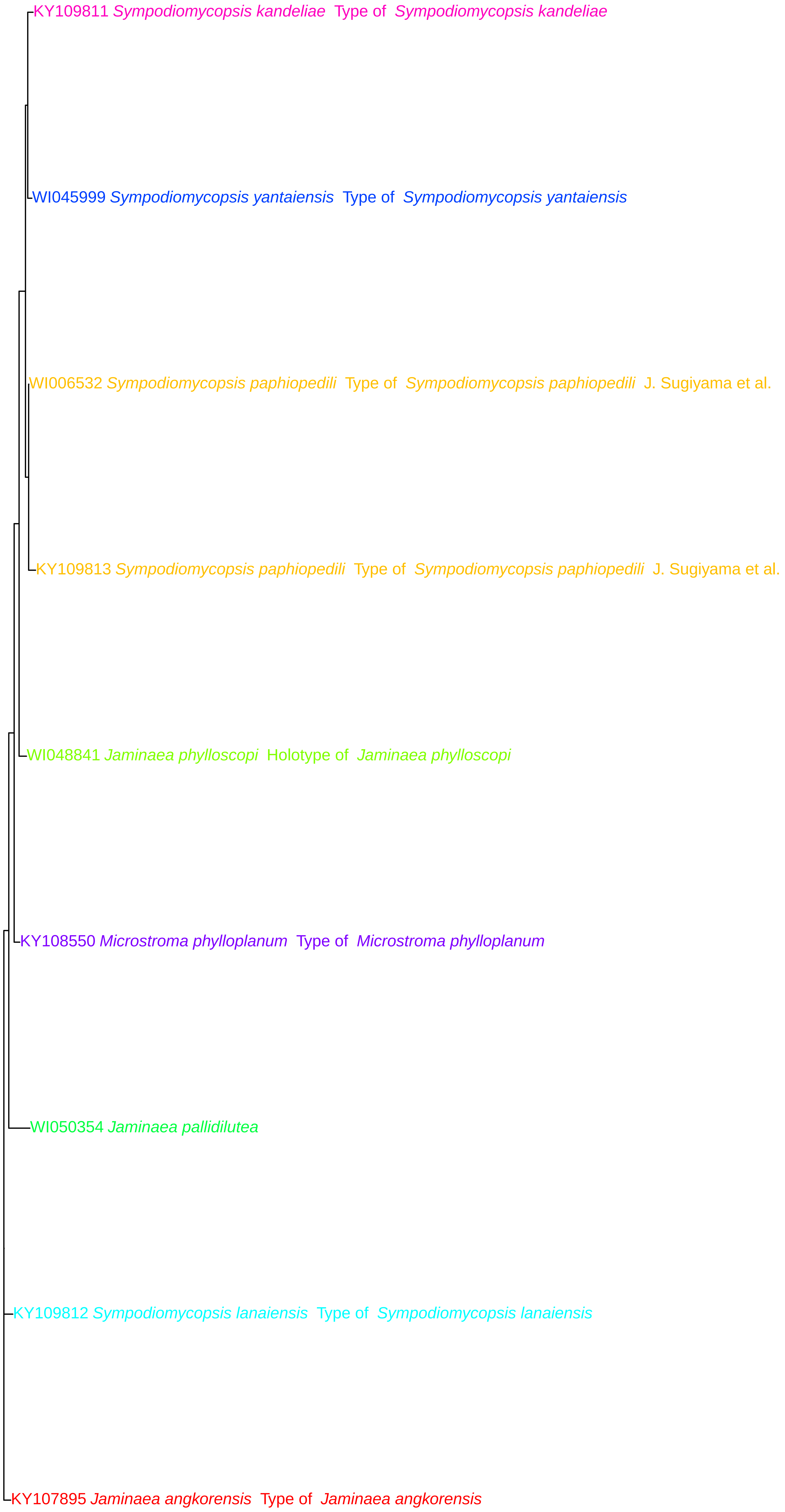

Moniliellales LSU

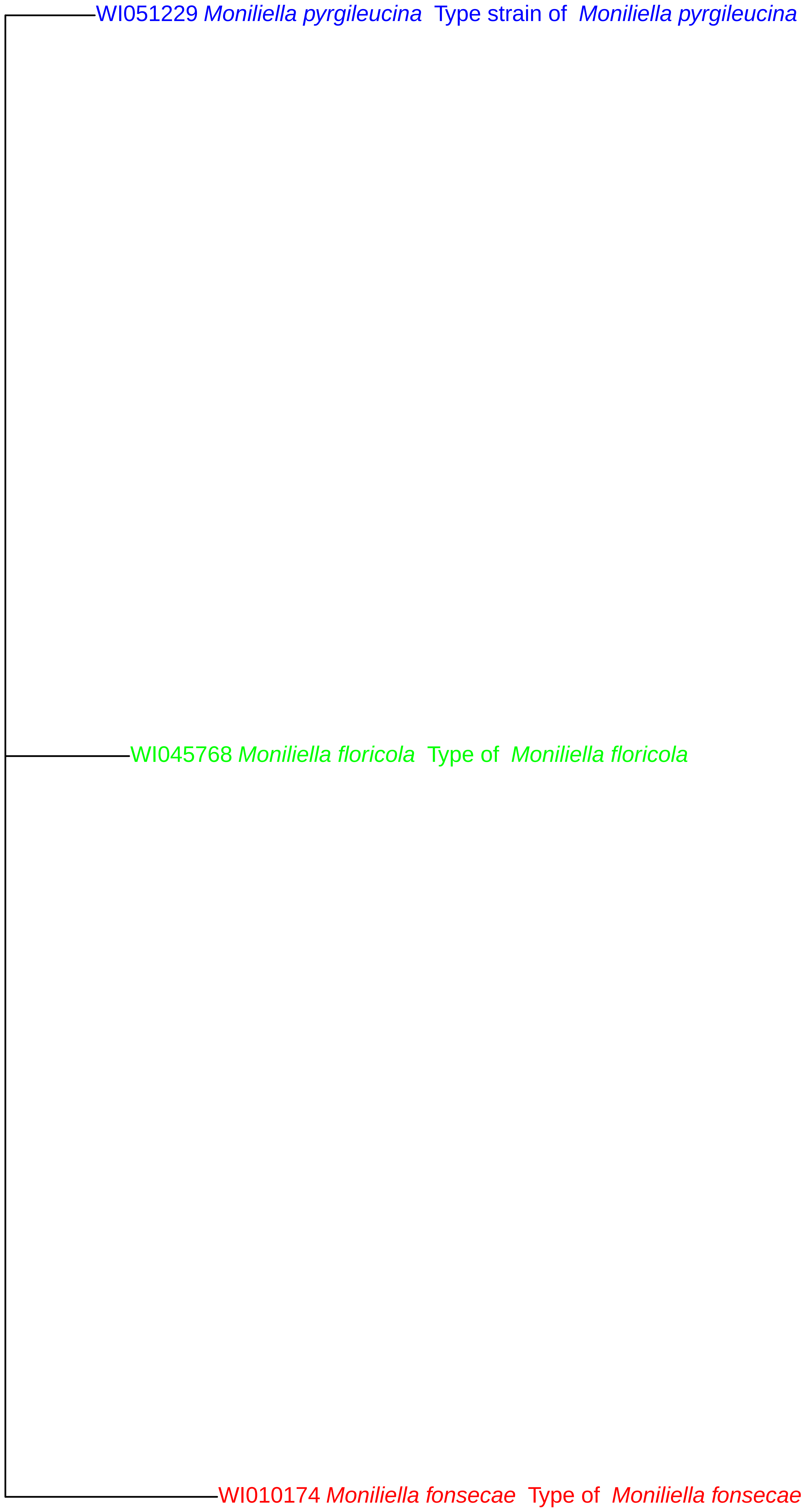

Phaffomycetales LSU

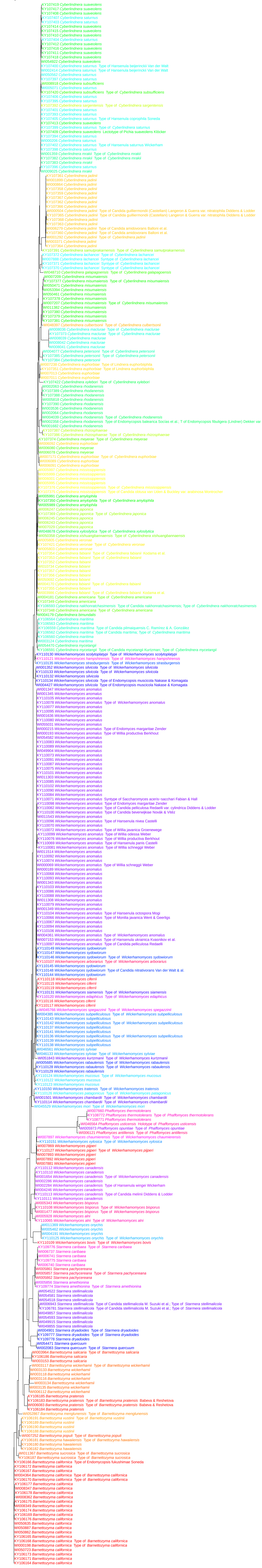

Pichiales LSU

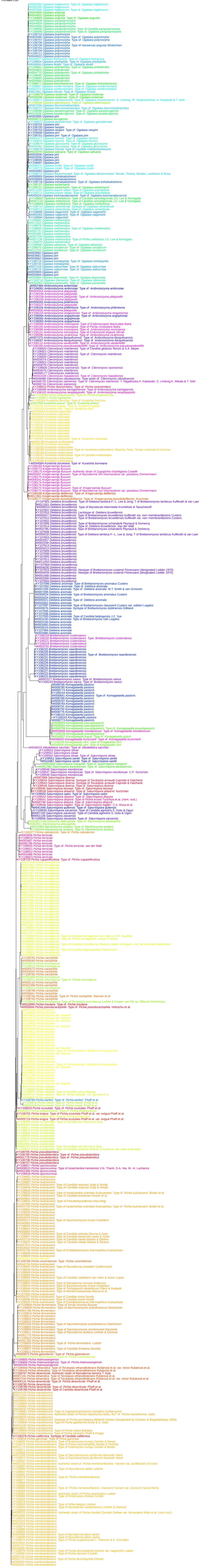

Saccharomycetales LSU

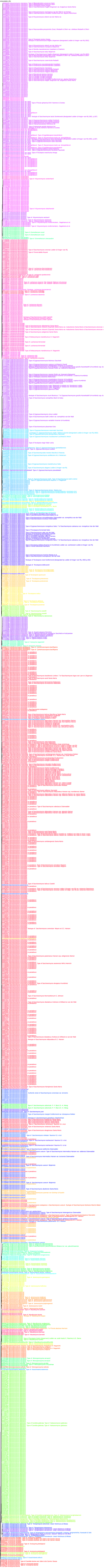

Saccharomycodales LSU

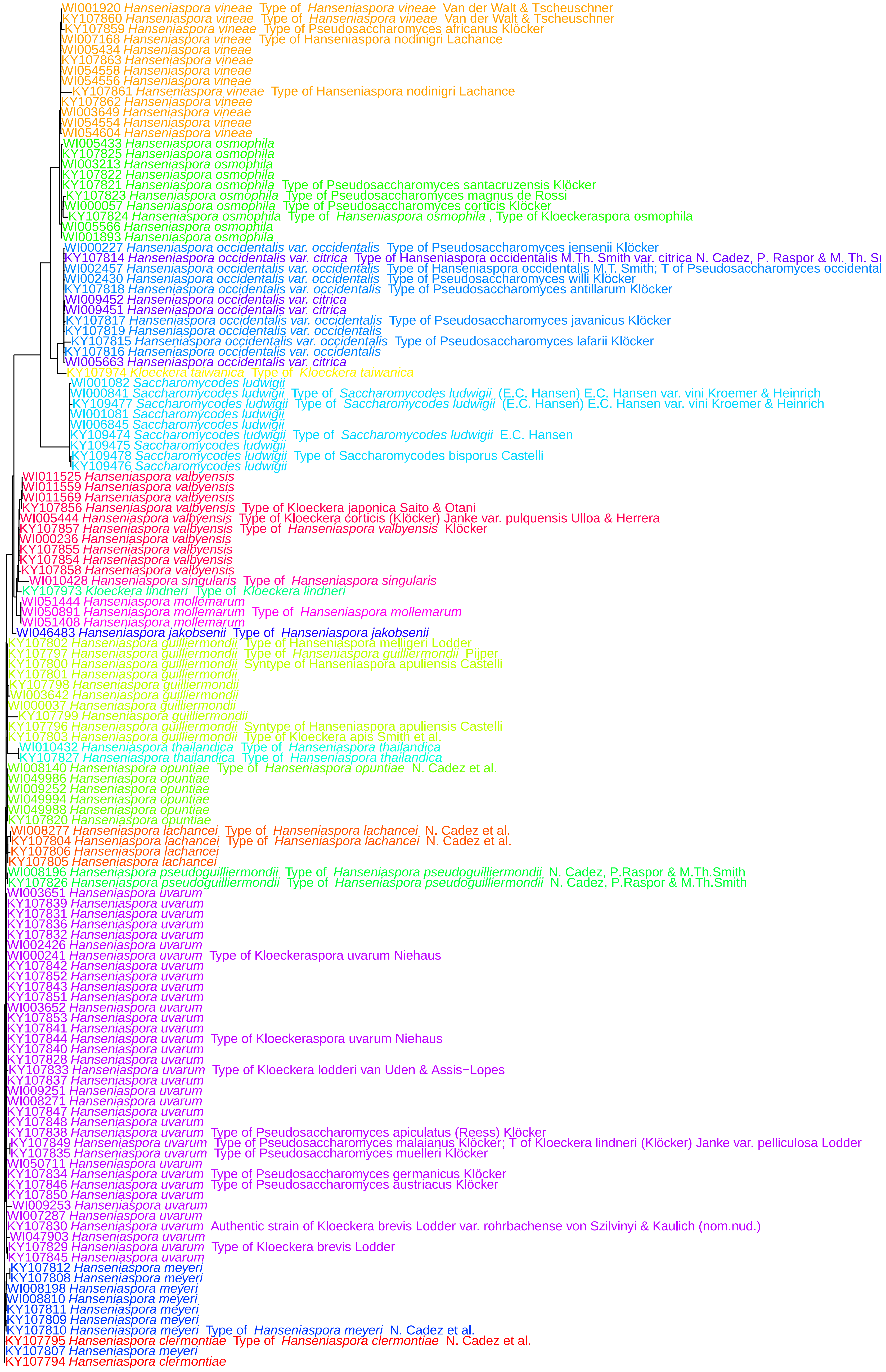

Sakaguchiales LSU

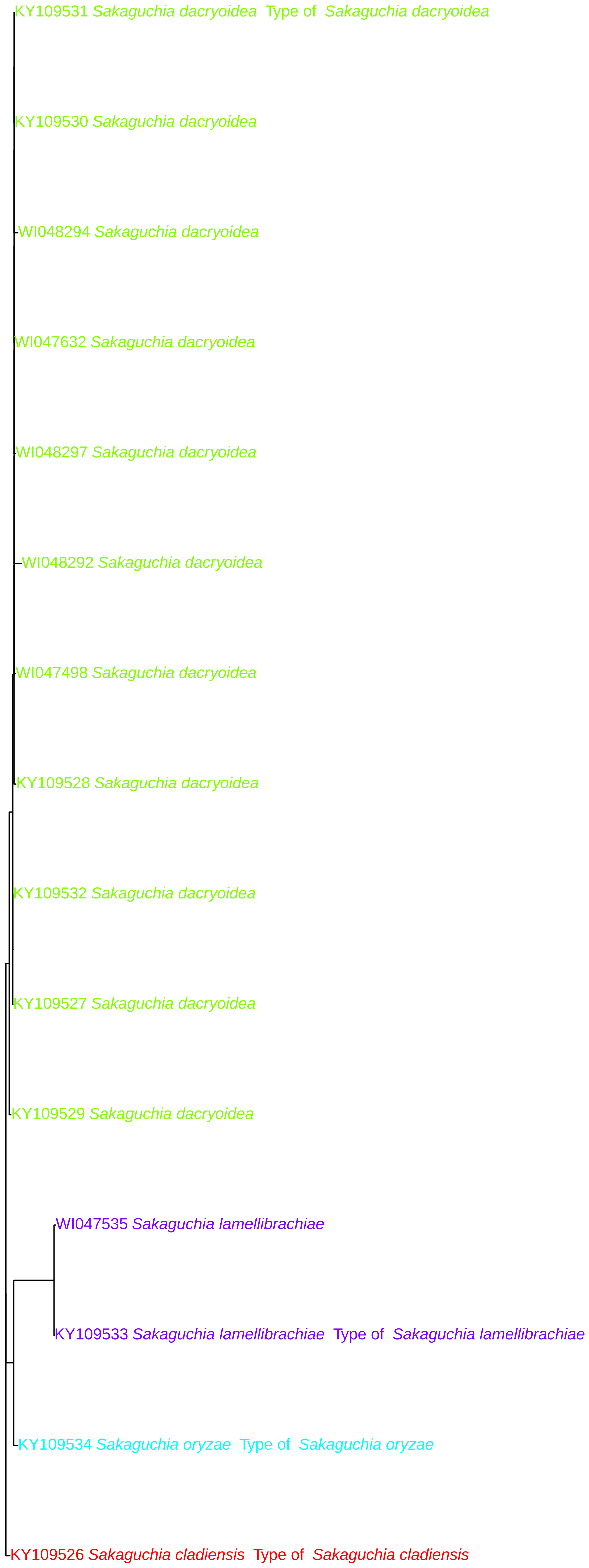

Schizosaccharomycetales LSU

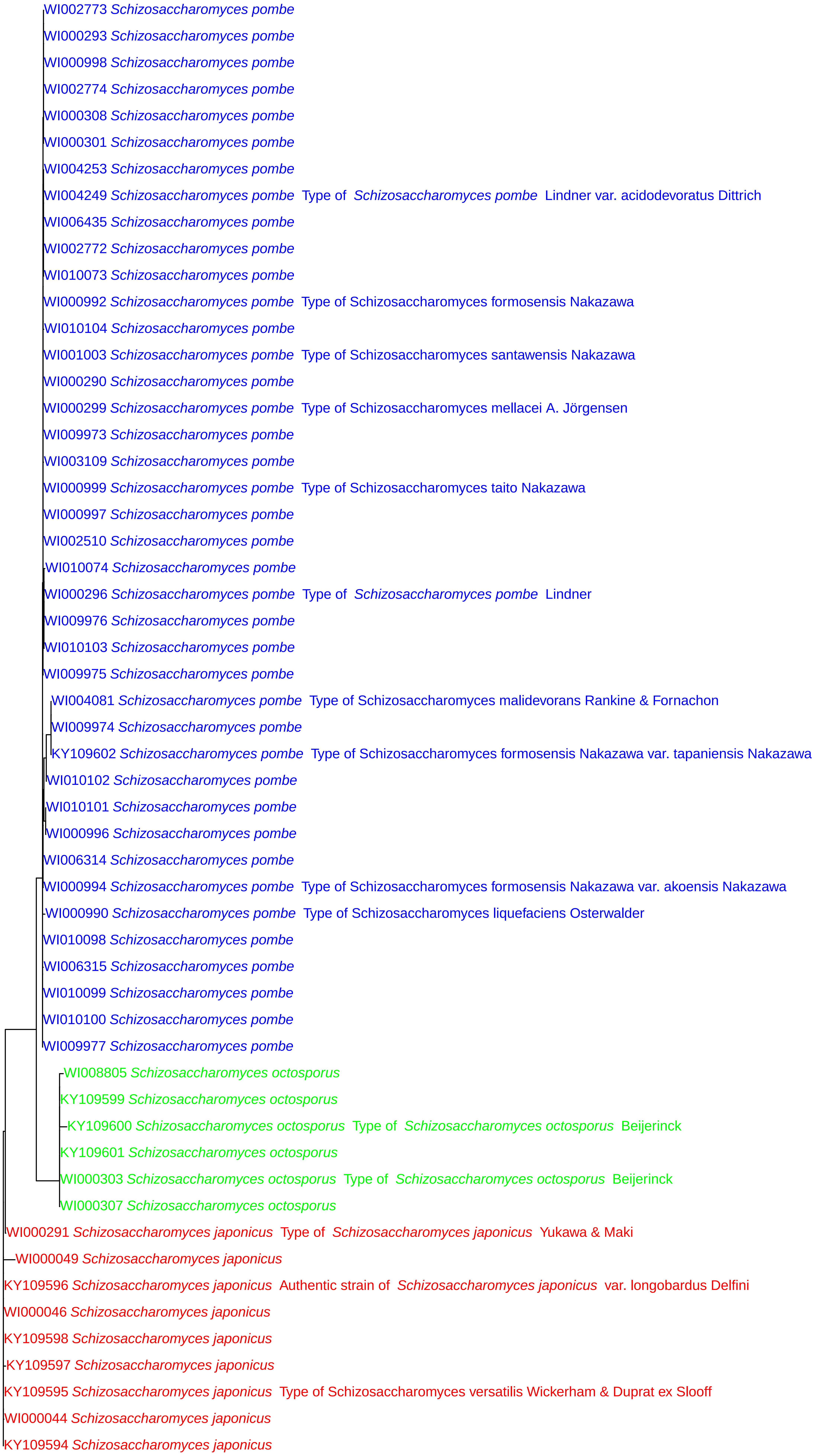

Serinales LSU

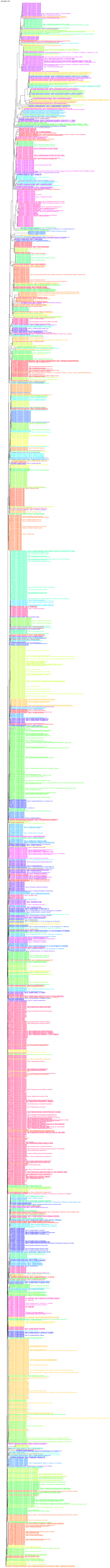

Spiculogloeales LSU

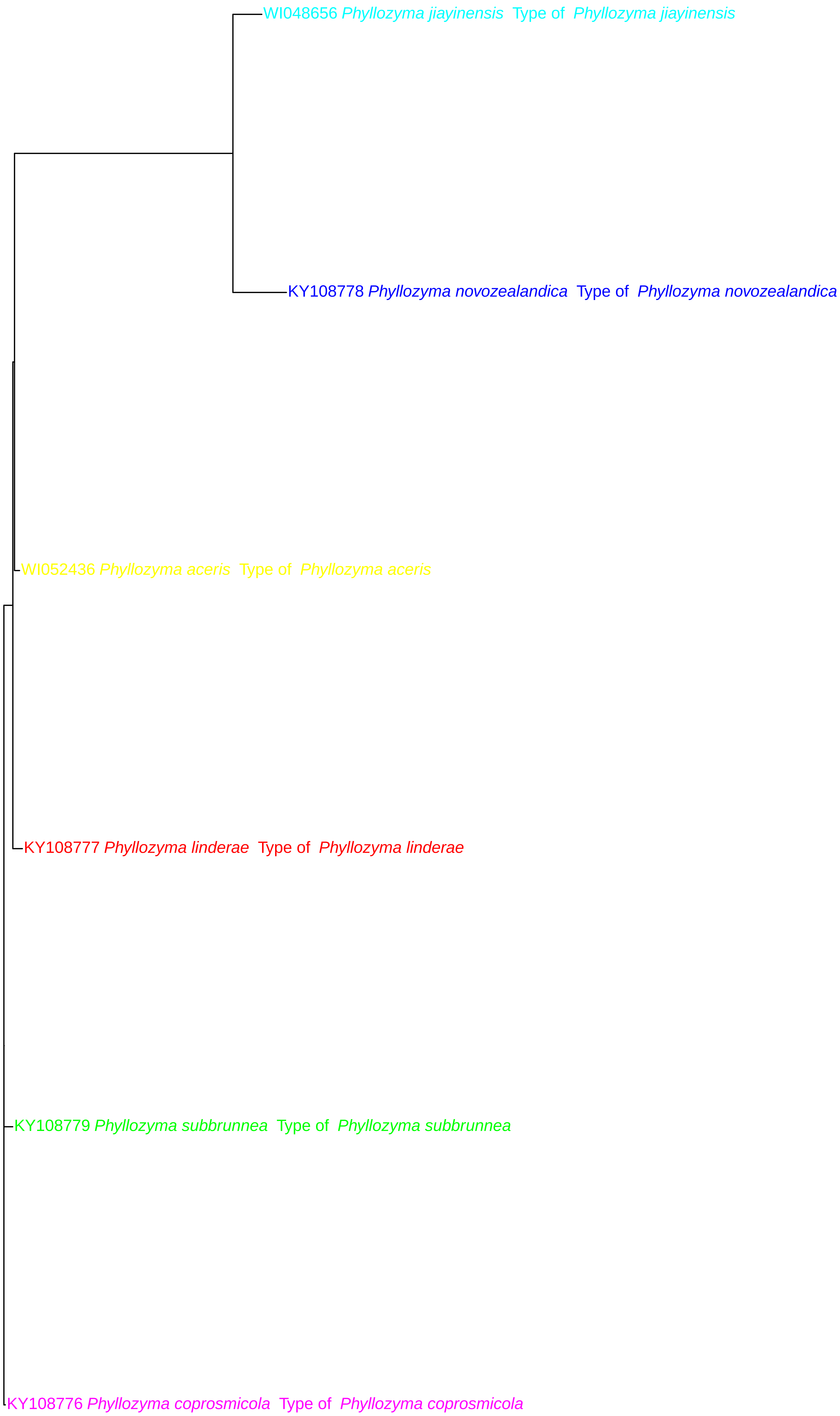

Sporidiobolales LSU

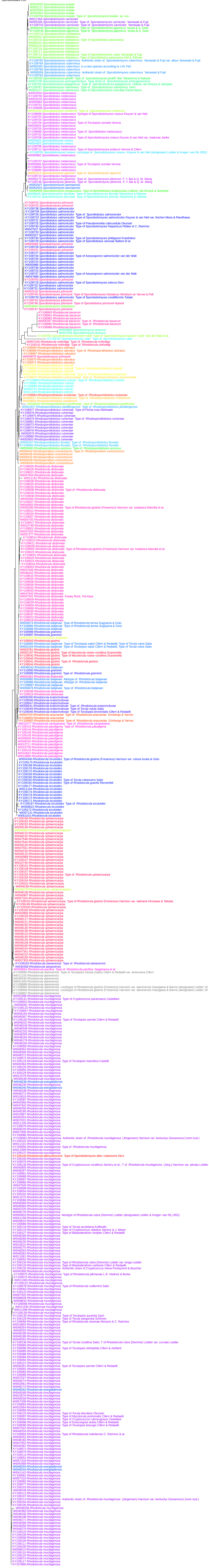

Sporopachydermiales LSU

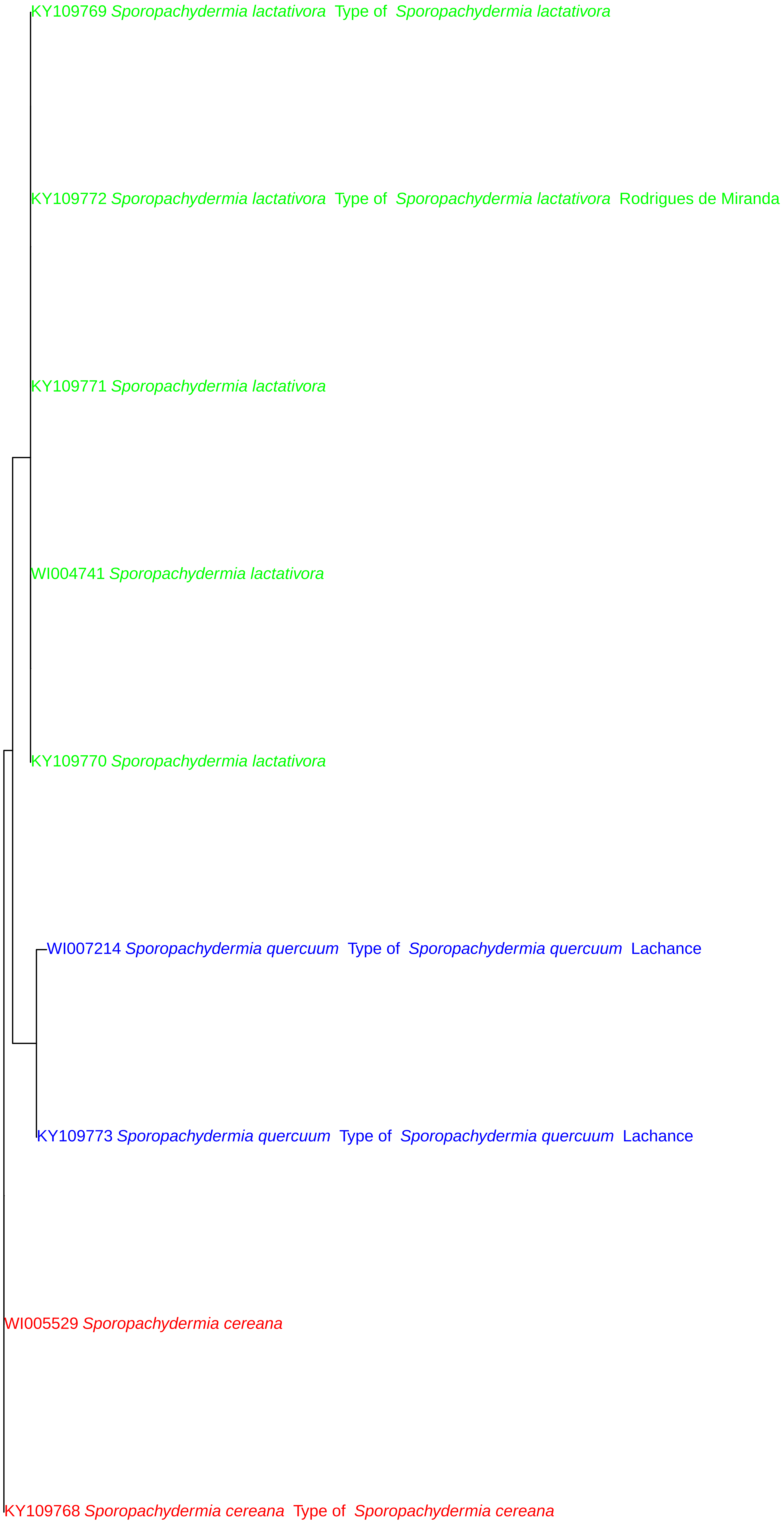

Taphrinales LSU

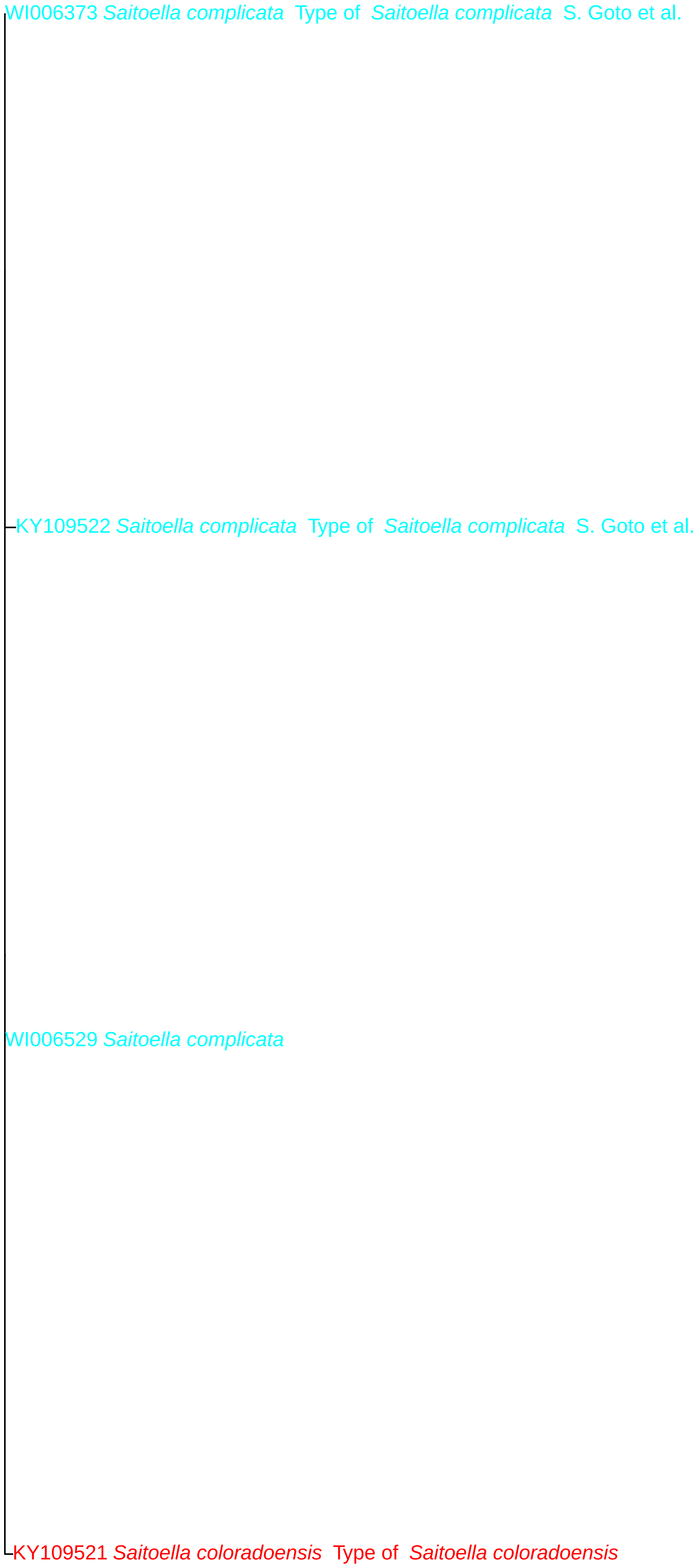

Tremellales LSU

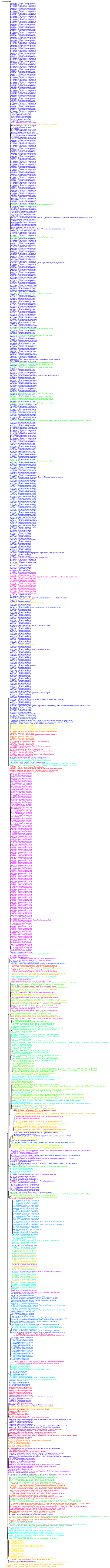

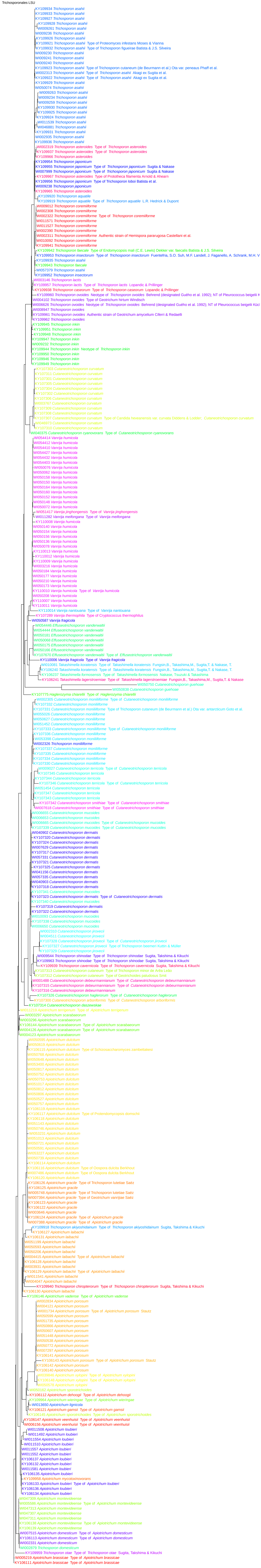
Trichosporonales LSU

Trigonopsidales LSU

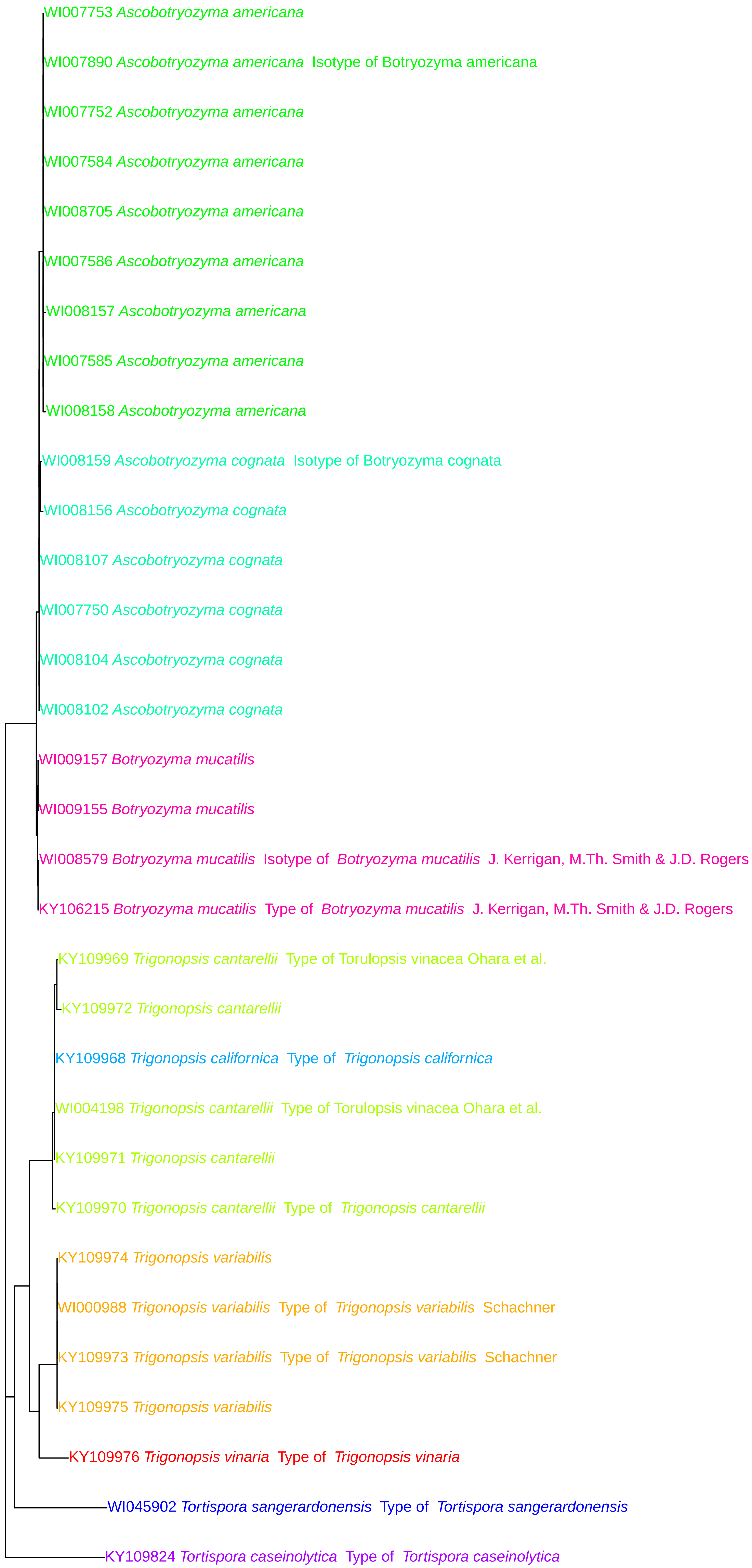

Ustilaginales LSU

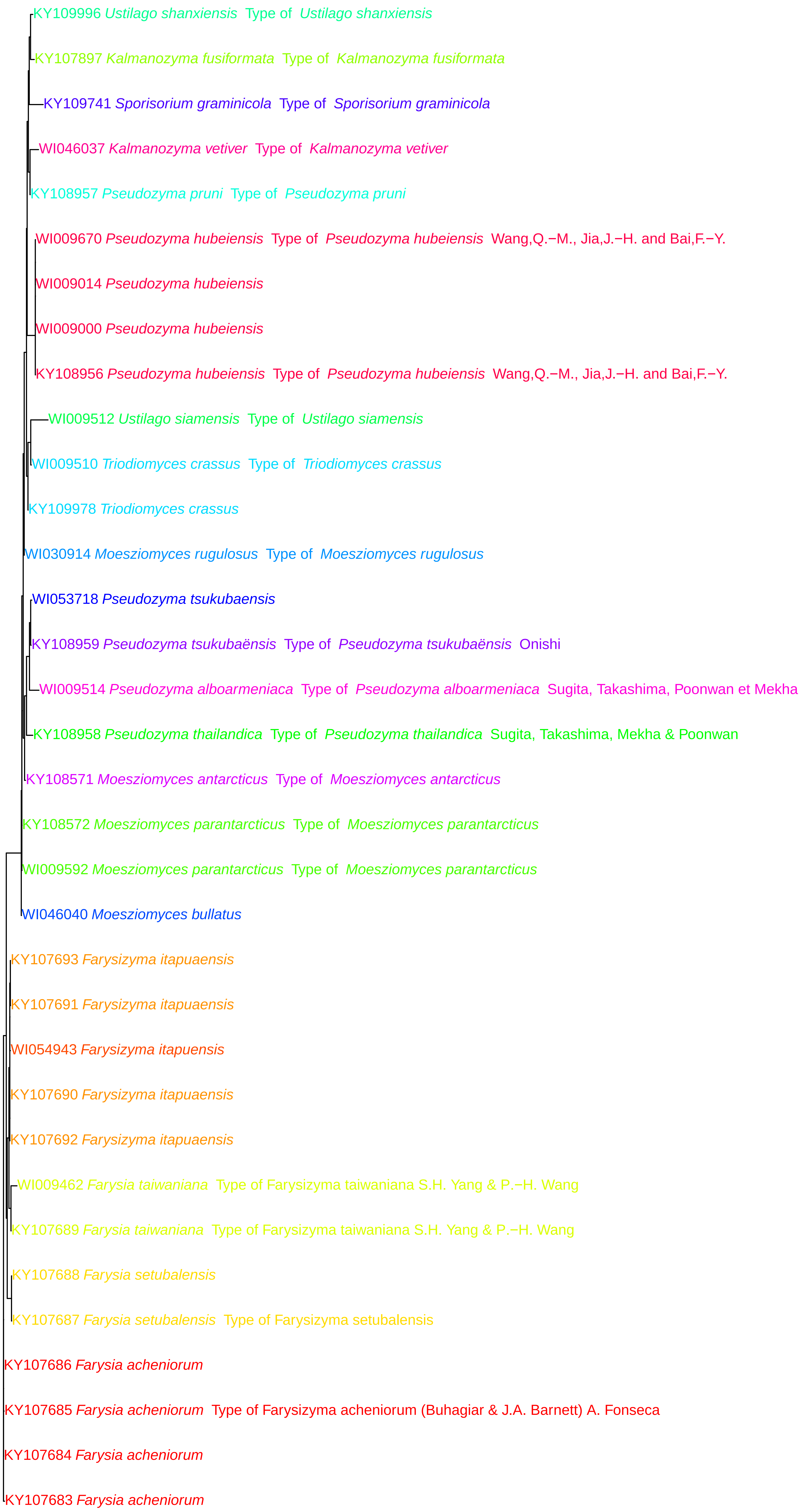

Supplement: Supplementary file 8 — Appendix S8: men70082‐sup‐0008‐AppendixS8.pdf. [file MEN-26-e70082-s008.pdf]
